# Clone-By-Clone Therapy Guidance in Luminal Breast Cancer *Via* Spatial Proteomics and Patient-Derived Tumoroid

**DOI:** 10.1016/j.mcpro.2026.101639

**Published:** 2026-08-12

**Authors:** Laurine Lagache, Antonella Raffo-Romero, Marie Duhamel, Yanis Zirem, Maire Vanseymortier, Isabelle Fournier, Nawale Hajjaji, Michel Salzet

**Affiliations:** 1 Univ. Lille, Inserm, CHU Lille, U1192, Protéomique Réponse Inflammatoire Spectrométrie de Masse (PRISM), Lille, France; 2 Breast Cancer Unit, Oscar Lambret Center, Lille, France; 3 Institut Universitaire de France, Ministère de l’Enseignement Supérieur, de la Recherche et de l’Innovation, Paris, France; 4 Equipe Labellisée Ligue Contre le Cancer, Lille, France

**Keywords:** breast cancer, mass spectrometry imaging, patient-derived tumoroids, drug discovery, precision therapy

## Abstract

Breast cancer (BC) remains the leading cause of cancer-related death among women worldwide, with 2.26 million new cases and ∼685,000 deaths reported in 2020 (Globocan 2020). A major challenge in treating BC, particularly luminal subtypes, is the pronounced molecular heterogeneity both between patients and within individual tumors. This intratumoral diversity underlies many cases of therapy resistance and relapse. Here, we present a theranostic approach that integrates spatial proteomics and patient-derived tumoroids (PDTs) to guide personalized treatment in luminal BC. Formalin-fixed, paraffin-embedded tumor tissues from three patients were analyzed by MALDI mass spectrometry imaging and spatially resolved microproteomics, mapping distinct clonal subpopulations and their protein expression profiles. Proteomic pathway analysis revealed subclone-specific vulnerabilities that are not apparent from standard histopathology. We exploited these insights to design alternative combination therapies tailored to each tumor’s molecular makeup. The efficacy of proteomics-guided regimens was then evaluated *in vitro* using PDTs established from the same patients, in direct comparison to conventional chemotherapy. Proteomic-informed treatments demonstrated significantly enhanced antitumor activity in the PDT models, yielding lower IC_50_ values and greater cell death than standard treatments. In several cases, PDTs exhibited resistance to conventional therapy, which was explained by the presence of proteomic resistance markers (such as EDIL3, CA12, PGK1, and CapG) in the corresponding tumor clones. These results underscore the potential of spatial proteomic profiling to expose actionable intratumoral heterogeneity and to inform more effective, clone-specific therapies. Our study highlights an innovative pipeline for luminal BC therapy guidance, combining MALDI mass spectrometry imaging and PDT drug testing, and advocates for the integration of spatial proteomics into precision oncology to improve treatment outcomes.

## Highlights

• Spatial proteomics reveals clonal-level intratumoral heterogeneity in luminal BC.
• MALDI MSI plus spatial proteomics reveals clone-specific vulnerabilities.
• Proteomics-guided combos outperform chemo in patient-derived tumoroids.
• Clone-specific markers (EDIL3, CA12, PGK1, CapG) explain treatment resistance.
• Theranostic pipeline combines imaging and assays for precision oncology.

## Graphical Abstract

**Figure:**
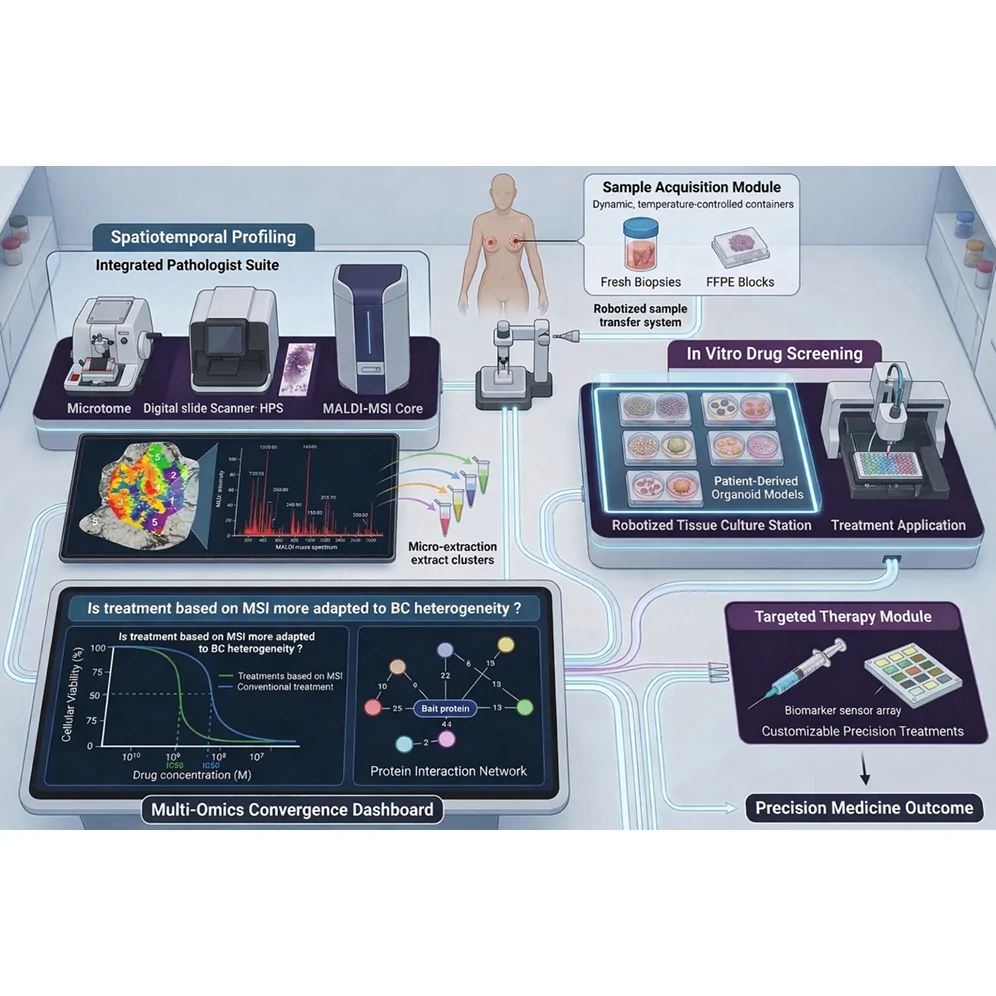

## In Brief

Intratumoral heterogeneity remains a major barrier to effective treatment in luminal breast cancer, driving therapeutic resistance and relapse. In this study, we combine MALDI mass spectrometry imaging–based spatial proteomics with patient-derived tumoroids to develop a theranostic workflow for personalized therapy selection. Spatially resolved proteomic profiling of tumor tissues reveals distinct clonal subpopulations with unique molecular signatures and pathway dependencies. Leveraging these data, we design clone-specific combination therapies, which are subsequently evaluated in matched tumoroid models. Proteomics-guided treatments demonstrate superior efficacy compared to standard chemotherapy and provide mechanistic insight into observed drug resistance through the identification of clone-associated protein markers. This integrated approach highlights the potential of spatial proteomics to inform precision oncology by enabling functionally validated, patient-specific therapeutic strategies.

Breast cancer (BC) is among the most prevalent and deadly malignancies in women, in part due to the difficulty of selecting optimal treatments for biologically complex tumors (1). Recent advances in genomic sequencing have revealed far greater intratumor heterogeneity than previously appreciated (2). Within a single breast tumor, numerous genetic mutations can give rise to divergent clonal expansions (3, 4). These clones are further shaped by nongenetic factors such as the tumor microenvironment, patient-specific conditions, and treatment pressures (3). Transcriptional, translational, and metabolic adaptations contribute additional layers of heterogeneity, yielding a mosaic of genetically related but functionally distinct subpopulations in each patient’s tumor (3). Such heterogeneity, both between patients and within an individual tumor, is a major obstacle for therapy (5). Standard treatment strategies, even when informed by broad subtype classification (*e.g*., “luminal A” *versus* “luminal B”), often fail to eradicate all clonal variants. Resistant subclones can survive and drive relapse (observed in ∼30% of BC cases), underscoring the need for more effective drug discovery strategies that account for tumor heterogeneity (6, 7).

Mass spectrometry–based spatial proteomics offers a powerful means to dissect tumor heterogeneity at the molecular level (8, 9). MALDI mass spectrometry imaging (MALDI MSI) and subsequent microproteomic analyses on breast tumors can resolve spatial distributions of proteins and identify distinct molecular subpopulations within a tissue (10). This approach enables *in situ* mapping of heterogeneous cell clusters and the discovery of region-specific drug targets, providing a rational basis for clone-specific therapy development (10, 11). At the same time, recent breakthroughs in three-dimensional culture have led to patient-derived tumoroids (PDTs) tumoroid, which faithfully recapitulate the histopathology and molecular features of the original tumor (12, 13, 14, 15, 16). PDTs serve as personalized *in vitro* models that preserve intratumoral diversity, overcoming the limitations of traditional 2D cell lines in drug screening. Given their genetic and phenotypic fidelity to patient tumors, PDTs are a promising platform to functionally test therapeutics in a patient-specific context (15).

Here, we combine spatial proteomics with PDT-based assays to demonstrate that accounting for intratumoral heterogeneity can improve therapy selection for luminal BC. In this study, three early-stage luminal breast tumors were analyzed using peptide MALDI MSI and spatially resolved proteomics to identify distinct clones and their activated pathways. Based on these proteomic profiles, we formulated alternative combination treatments targeting the specific vulnerabilities of each tumor’s subclones. We then evaluated the efficacy of these proteomics-guided therapies *versus* conventional chemotherapy in matched PDTs derived from the same patients. Our results show that treatments informed by spatial proteomics achieved more potent antitumor effects in PDTs than standard regimens. Notably, in cases where PDTs exhibited resistance to standard chemotherapy, the proteomic data provided an explanatory mechanism (*e.g*., upregulation of epithelial-mesenchymal transition (EMT) and drug efflux proteins) and a path to overcome this resistance with tailored agents. Taken together, this work establishes a robust workflow for personalized drug discovery in luminal BC, from tissue imaging and proteomic analysis to *ex vivo* drug testing and highlights the value of integrating spatial proteomics and tumoroids models to guide therapy for heterogeneous tumors.

## Experimental Procedures

### Experimental Design and Statistical Rationale

For MALDI imaging and proteomics on tissues, n = 3 breast cancer tissues formalin-fixed, paraffin-embedded (FFPE) bloc were use, among them three tissue sections were cut for each bloc. Tumor specimens were obtained from three patients diagnosed with early stage luminal breast cancer (tumor 1: 100%ER+/100% PR+, tumor 2: 100% ER+/80%PR+, and tumor 3: 95% ER+/95%PR+). All patients were adult women with no prior cancer treatment and no known genetic or comorbid conditions that could influence tumor biology. No other covariate characteristics were considered. Written informed consent was obtained from each patient, and the study protocol was approved by the institutional research ethics committee (Lille Tumor Bank, approval ID RCB 2014-A00185-42) in accordance with the Declaration of Helsinki and Good Clinical Practice guidelines. FFPE tissue blocks from surgical resection or biopsy were used for proteomic analyses. In addition, this study employed PDTs generated from fresh tissue biopsies obtained from the same patients with written informed consent, under approval from the medical ethics committee of the Centre Oscar Lambret (NCT05007379) in accordance with the Declaration of Helsinki and Good Clinical Practice guidelines, after approval by a French ethics committee (CPP Ile de France II, Research Project n°21.04.21.68809 RIPH2 HPS). Tumoroid relevant clinicopathological data (*e.g.,* hormone receptor status and histological grade) were recorded, and all samples were de-identified prior to analysis.

#### Tissue Sectioning

FFPE tumor tissues were sectioned on a microtome (Leica Microsystems). For each tumor, 5 μm section were led on a polylysine-coated glass slide for hematoxylin-phloxine-saffron (HPS) histological staining and for pathological review. For MALDI MSI, an 8 μm section on an indium tin oxide-coated conductive slide (LaserBio Labs) and for spatial proteomics (on-tissue digestion and peptide extraction) a 20 μm section on a polylysine slide. All slides were dried and stored at room temperature until use.

#### Histological Staining (HPS)

The 5 μm sections were stained with HPS using an automated stainer. Briefly, slides were deparaffinized in xylene (2 × 5 min), rehydrated through graded ethanol (100% and 95%) to water, and then sequentially stained: hematoxylin for nuclear staining, brief differentiation in 0.8% HCl, water rinse, cytoplasmic counterstain with phloxine, and collagen-specific stain with saffron. After staining, slides were dehydrated in ethanol, cleared in xylene, and coverslipped. Histopathological analysis was performed by a certified pathologist (N.H) to identify regions of interest and to correlate with proteomic cluster findings. The HPS images provided an anatomical reference for MALDI MSI segmentation maps.

#### Statistical Significance of the Results

For MSI experiments, each tissue section was analyzed once. MSI data processing was performed on19193 pixels and 25,100 molecular features for tumor 1, 99,049 pixels and 25,100 features for tumor 2, and 98,272 pixels and 25,100 features for tumor 3.

Bulk proteomic analyses of tumoroids and spatial proteomic extractions were performed in triplicate (biological replicates), resulting in the identification of 8818 proteins (Supplementary Spreadsheet 1). For conditioned media proteomic data, statistical significance was assessed using one-way ANOVA to detect differential protein abundance between experimental conditions. A significance threshold of *p* ≤ 0.01 was applied. Where appropriate, post hoc comparisons were performed to identify group-specific differences. Prior to visualization, protein intensities were Z-score normalized across rows, and heat maps were generated accordingly.

Tumoroid viability experiments for IC50 determination was performed in triplicate for each condition.

For peptide biomarker discovery from MSI data, statistical significance was evaluated using a nonparametric Kruskal–Wallis test to account for non-normal data distributions and unequal variances. To control the family-wise error rate arising from multiple testing, Bonferroni correction was applied to adjust *p*-values. Peptide features with adjusted *p*-values below the significance threshold were considered statistically significant. Data are reported as medians in Supplementary Spreadsheet 2.

### Reagents

Unless otherwise stated, all chemicals were analytical grade. Key reagents and materials included: HPLC-grade water, ethanol, methanol, and acetonitrile (Thermo Fisher Scientific); trifluoroacetic acid (TFA), formic acid, and other solvents (Sigma-Aldrich); α-cyano-4-hydroxycinnamic acid matrix and aniline additive (Sigma-Aldrich); porcine trypsin (sequencing grade, Promega); and Matrigel Basement Membrane Matrix (Corning) for 3D culture. Indium Tin Oxide slides were purchased from LaserBio Labs, whereas the poly-lysine coated slides were from EprediaTM.

### MALDI Mass Spectrometry Imaging Workflow

#### Tissue Preparation for MALDI MSI

FFPE sections (8 μm on indium tin oxide slides) were processed to expose proteins for imaging. Slides were first deparaffinized (2 × 5 min in xylene) and rehydrated in graded ethanol baths (*e.g*., 2 × 90% ethanol, 1 × 70% ethanol, 1 × 50% ethanol, then distilled water). An antigen retrieval step was then performed to unmask proteins: slides were immersed in 20 mM Tris buffer (pH 9.0) at 90 °C for 20 min, followed by two quick washes in 10 mM ammonium bicarbonate. Slides were dried under vacuum.

#### On-Tissue Trypsin Digestion

A solution of trypsin (40 μg/ml in 50 mM NH_4_HCO_3_) was uniformly sprayed onto the tissue sections using an automated sprayer (HTX M5 Sprayer, HTX Technologies). The slides were incubated in a humidified chamber (containing a small dish of 50% methanol to maintain moisture) at 56 °C overnight, allowing tryptic peptides to be generated throughout the tissue. After digestion, slides were again dried under vacuum.

#### Matrix Application

A MALDI matrix solution (α-cyano-4-hydroxycinnamic acid at 10 mg/ml in 70% acetonitrile/0.1% TFA, with 0.007% aniline as a chemical enhancer) was sprayed evenly across the digested tissue using the HTX Sprayer. Matrix deposition was done with gradual layering to avoid delocalization, and the coated slides were dried before analysis (17, 18).

#### MALDI MSI Data Acquisition

Mass spectrometry imaging was performed on a Bruker rapifleX MALDI-TOF mass spectrometer (Bruker Daltonics) equipped with a SmartBeam 3D laser. Data were acquired in positive ion reflectron mode over an *m/z* range of ∼360 to 3200. The raster step size (pixel size) was set to 60 μm, and ∼200 laser shots were accumulated per pixel to generate each mass spectrum. External calibration was applied using peptide calibration standards as needed. The resulting dataset comprised a spatially registered mass spectrum at each x,y coordinate of the tissue section.

#### MALDI MSI Data Processing

The raw imaging data were converted to imzML format (using Bruker’s SCiLS Lab or imzML converter) with a bin size of 0.3 and a mass range of 360 to 3200 *m/z* resulting in 25,100 features. The datasets are archived in Zenodo and accessible here: https://doi.org/10.5281/zenodo.19114048. We processed data following a “dry proteomics” pipeline for imaging analyses (18) on HPC mesocentre of Lille. Spectra were normalized (root-mean-square normalization) to account for intensity differences across the image. To reduce dimensionality and noise, we performed singular value decomposition on the spectral data matrix, retaining components that preserved the significant variance (>90%) in the dataset. Unsupervised segmentation of the tissue was then carried out by k-means++ clustering (MATLAB Statistics Toolbox), using the cosine distance metric between spectra (19). The optimal number of clusters was estimated with the silhouette criterion: we iteratively evaluated clustering results for a range of cluster numbers and selected the number that maximized the average silhouette score (indicating well-separated, cohesive clusters). Once the number of molecular clusters was fixed, k-means++ was run to assign each spectrum (pixel) to a cluster, and a false-color segmentation map was generated in which each cluster is represented by a distinct color (18, 20). This proteomic segmentation map delineates regions of the tissue with similar protein expression profiles, *i.e.,* putative tumor “clones” or other tissue compartments. Segmentation results were carefully overlaid on the histology image to correlate molecular regions with histopathological features (*e.g*., tumor *versus* stroma, or different tumor morphologies).

Following molecular segmentation, cluster-associated MSI features were extracted and further analyzed and further analyzed using Profiler (21), with the assistance of MSI2Profiler, an additional tool designed to convert MSI data into a format compatible with the Profiler workflow. MSI2Profiler was first used to compress the MSI data from the original two-dimensional imzML format into one-dimensional tabular data (CSV format), enabling downstream statistical analyses. The resulting files were then imported into Profiler using the “Tabular and Omics Data” workflow (Step 3). To identify discriminant m/z features among clusters and generate boxplots, the biomarkers module of Profiler was employed. Statistical comparisons were performed using the nonparametric Kruskal–Wallis test, followed by Benjamini–Hochberg correction for multiple testing. For comparative analyses of cluster molecular signatures, peak picking was first performed in DataLab Explorer using the preprocessing module and a local maxima detection approach. The resulting peak lists were subsequently analyzed using the comparison module to generate UpSet plots, allowing visualization of shared and cluster-specific m/z features. Within the same module, cluster similarity was assessed using Cohen’s kappa coefficient. As this metric requires categorical input, Profiler automatically discretizes feature intensities into three abundance categories (low, medium, and high) prior to analysis. Cohen’s kappa was then computed between cluster-specific molecular profiles, providing a quantitative measure of agreement that captures both similarities and discrepancies across segmented regions.

### Spatially Resolved Proteomics of Tumor Regions

To characterize the protein expression of each molecular cluster in depth, we performed spatially resolved microproteomics on selected regions of the tissue corresponding to the MALDI clusters.

#### On-Tissue Microdigestion and Peptide Extraction

From each MALDI MSI cluster, a representative region of approximately 0.8 mm^2^ was targeted for peptide extraction. An automated chemical printer (Shimadzu CHIP-1000) was used to deposit trypsin solution onto the region in a precise array of small droplets. Specifically, trypsin (40 μg/ml in 50 mM NH_4_HCO_3_) was spotted as a grid of 4 × 4 droplets (each ∼200 μm in diameter, covering a total area of 800 μm × 800 μm). The robot performed ∼1200 droplet deposition cycles per spot over ∼3 h, delivering ∼0.8 nl per droplet in repetitive fashion to ensure sufficient proteolysis (total ∼0.1 μg trypsin per spot). The tissue under each droplet was digested *in situ*, generating peptides from that localized region. After microdigestion, peptides were extracted from the tissue by liquid microjunction extraction using a TriVersa NanoMate system (Advion Biosciences) in liquid extraction surface analysis mode. We performed two consecutive extractions per spot for each of three solvent formulations: (1) 0.1% TFA in water, (2) 80:20 acetonitrile/0.1% TFA, and (3) 70:30 methanol/0.1% TFA. For each extraction, ∼2 μl of solvent was aspirated, and a small droplet (∼0.8 μl) was placed onto the tissue spot to dissolve peptides, followed by gentle aspiration. The six extracts from a given spot (two per solvent) were pooled in a single vial. Finally, 50 μl of acetonitrile was added to each pooled extract to aid subsequent evaporation, and samples were dried in a SpeedVac vacuum concentrator. The dried peptide mixtures were stored at −20 °C until LC–MS/MS analysis. This spatial proteomics workflow was done in triplicate for each cluster (*i.e.,* three spots from the same tissue section per cluster) to ensure reproducibility of protein identification and quantification.

### Bulk Proteomic

For comparison to the spatially resolved data, we also carried out bulk proteomic analysis of the original tumors and derived tumoroids. A section of each FFPE tumor (or a portion of fresh-frozen tumor, if available) and a pellet of tumoroid (collected before drug treatment) were subjected to bulk protein extraction. Tissues and tumoroids were lysed in RIPA buffer (50 mM Tris-HCl pH 8, 150 mM NaCl, 1% NP-40, 0.1% SDS, plus phosphatase and protease inhibitors). Lysates were sonicated on ice (3 × 30 s) and cleared by centrifugation. Protein concentrations were measured (Bradford assay) and standardized.

#### Filter-Aided Sample Preparation

For each sample, 100 μg of protein was reduced with dithiothreitol (100 mM DTT in 8 M urea, 0.1 M Tris pH 8.5) at 95 °C for 15 min. The denatured, reduced protein solution was transferred to a 10 kDa ultrafiltration unit (Amicon). Urea buffer (8 M urea in 0.1 M Tris pH 8.5) was added, and the unit centrifuged at 14,000×*g* for 30 min (this wash was repeated to remove detergents). Proteins on the filter were alkylated by adding 100 μl of 50 mM iodoacetamide in urea buffer and incubating in the dark for 20 min, then centrifuged. The filter was washed three times with 50 mM ammonium bicarbonate. Proteolytic digestion was carried out by adding 50 μl of Lys-C/trypsin solution (20 μg/ml in 50 mM ammonium bicarbonate) to the filter and incubating overnight at 37 °C. Tryptic peptides were collected by centrifugation, and two additional 100 μl washes of the filter with ammonium bicarbonate were combined with the flow-through. Peptide samples were acidified with 0.1% TFA and dried in a SpeedVac. These digests (from whole tumor and whole tumoroid) were analyzed in parallel with the spatially extracted peptides to compare global protein profiles.

### NanoLC–MS/MS and Data-Independent Acquisition-Parallel Accumulation Serial Fragmentation Analysis

All peptide samples were reconstituted in 0.1% formic acid and analyzed by nanoflow liquid chromatography coupled to tandem mass spectrometry (nano-LC–MS/MS). Peptide separation was performed using an Evosep One nano-LC system interfaced with a Bruker timsTOF fleX mass spectrometer. Peptides were loaded onto an 8 cm × 150 μm inner diameter C18 analytical column packed with 1.5 μm particles and eluted using the Evosep 60 samples-per-day segmented gradient. Mobile phase A consisted of 0.1% formic acid in water, and mobile phase B consisted of 0.1% formic acid in acetonitrile.

Mass spectrometric data were acquired using data-independent acquisition parallel accumulation serial fragmentation (dia-PASEF) (22). In each 10 acquisition cycles, one full MS1 scan was followed by dia-PASEF MS/MS scans covering a precursor mass range from m/z 100 to 1700. Ion mobility separation was performed in parallel, with the ion mobility range set from 1/K_0_ = 0.65 to 1.42 V s cm^−2^. The accumulation time and ion mobility ramp time were both set to 100 ms for MS1 and MS2 scans. Each MS1 scan and each dia-PASEF MS/MS scan therefore lasted 100 ms plus a short transfer time. A complete dia-PASEF cycle comprising one MS1 scan and 22 dia-PASEF MS/MS scans resulted in a total cycle time of approximately 1.06 s.

The timsTOF Flex was operated in high-sensitivity mode. Collision energy was ramped linearly as a function of ion mobility, ranging from approximately 59 eV at high ion mobility (1/K_0_ = 1.6 V s cm^−2^) to 20 eV at low ion mobility (1/K_0_ = 0.6 V s cm^−2^), enabling efficient fragmentation across a broad range of peptide ions. Ion mobility calibration was performed using three Agilent ESI Tuning Mix ions with known m/z and ion mobility values (m/z 622.02 at 1/K_0_ = 0.98 V s cm^−2^, m/z 922.01 at 1/K_0_ = 1.19 V s cm^−2^, and m/z 1221.99 at 1/K_0_ = 1.38 V s cm^−2^).

### Proteomic Data Processing and Analysis

Raw dia-PASEF data files were processed with DIA-NN software (version 2.1) for peptide identification and quantification (23). A project-specific spectral library was first generated from the data: DIA-NN was configured with the UniProt *Homo sapiens* reference proteome (release 2024_12, ∼82,000 entries) as the protein sequence database. Enzyme specificity was set to trypsin with up to two missed cleavages allowed. Carbamidomethylation of cysteine was specified as a fixed modification, and methionine oxidation was allowed as a variable modification (up to three modifications per peptide). Peptide identification parameters included a peptide length range of 7 to 30 amino acids, a precursor charge state range of 1 to 4, and a precursor *m/z* range of 100 to 1700, consistent with the dia-PASEF acquisition method. Fragment ion *m/z* values were considered in the range of 200 to 1700. Both mass accuracy (MS/MS mass tolerance) and MS1 accuracy (MS1 mass tolerance) were set to 15.0 (ppm). Library generation was performed using the “IDs, RT & IM profiling” option, enabling retention time and ion mobility–dependent spectrum prediction directly from the experimental data. DIA-NN’s neural network classifier was run in single-pass mode. False discovery rate (FDR) control was applied at multiple levels, with a precursor-level FDR threshold of 0.1% and a protein-level FDR of 1%. Protein inference was performed at the gene level, consolidating unique peptides per gene. Quantification was carried out using the robust LC (high-accuracy) strategy, with retention time–dependent cross-run normalization enabled to correct for technical variation between LC–MS runs.

The DIA-NN output consisted of a quantitative matrix of gene-level protein abundances across all samples, with each sample corresponding to a specific tumor or tumoroid region or bulk sample. All proteomics raw and processed data have been deposited in the PRIDE repository under the accession number PXD069792 (Username: reviewer_pxd069792@ebi.ac.uk) (Password: OH6dz6MeUqAA).

#### For Quantitative Analysis

Proteins identified in each dataset were filtered and normalized to ensure high-quality comparisons. We retained only proteins detected in at least 66% of the samples of interest (*e.g.,* in at least two of the three replicate extracts for a given cluster). Intensity values were log_2_-transformed, and missing values were imputed using a low-end imputation (random draw from a normal distribution centered just below the detection limit, with a width reflecting noise, often referred to as a “shifted Gaussian” imputation). This approach preserves data structure while approximating undetected low-abundance proteins. The processed data were used to generate heatmaps and perform unsupervised clustering (using Profiler software (21)) to visualize protein expression patterns across tumor regions and conditions.

#### Differential Expression and Pathway Analysis

To identify proteins significantly varying between groups (*e.g.,* between different tumor clones), we performed statistical testing such as multiple-sample ANOVA with a significance threshold of *p* < 0.01 (after controlling for false discovery using the Benjamini–Hochberg method). Sets of differentially expressed proteins were further analyzed for enriched biological pathways and processes. Pathway enrichment analysis was conducted using the enrichment module of Profiler software (21), applying an overrepresentation analysis approach on the list of significantly differentially expressed proteins. This analysis was performed against reference databases such as Hallmark pathways, Gene Ontology, and KEGG, with significance thresholds (*e.g.,* q < 0.05) to highlight key functional differences between clusters. Therefore, enrichment results reflect differences between tumor clones or regions, rather than absolute pathway expression levels. This approach reduces potential bias related to overall protein abundance and focuses on biologically meaningful variations between groups. To assess druggability, we annotated proteins of interest according to the Illuminating the Druggable Genome categories: “Tclin” (clinically validated targets with approved drugs) (24), “Tchem” (targets with known bioactive compounds) (25), “Tbio” (biologically significant but not yet targeted) (26), and “Tdark” (little-known proteins) (27, 28). This categorization provided a framework for selecting candidate therapeutic targets from the proteomic data.

### Tumoroid Culture and Drug Treatment Assays

#### Primary Breast Cancer Tumoroid Culture and Drug Treatment-PDT

Fresh tumor samples corresponding to each of the three patients were used to generate tumoroids as described before (12, 13), briefly tissues were finely minced and enzymatically dissociated during 2h at 37 °C in digestion medium of consisting Collagenase Type IV (Sigma-Aldrich) and hyaluronidase. After digestion, the cell suspension was filtered through a 100 μm cell strainer and centrifuged at 300×*g* for 5 min. In cases where red blood cell contamination was visible, red blood cell lysis step was performed. The resulting cell pellet was resuspended in reduced growth factor-soluble basement membrane matrix for tumoroid culture (Matrigel, Corning) and polymerized at 37 °C for 15 to 30 min, after which 500 μl of tumoroid culture medium was gently added to each well. The culture medium was formulated as previously described tumoroid, comprising Advanced DMEM/F12 supplemented with 1× GlutaMAX, 10 mM Hepes, 1× B-27 supplement (all Thermo Fisher), 1 mM N-acetylcysteine, 5 mM nicotinamide, and a cocktail of human recombinant growth factors including 250 ng/ml R-spondin1, 5 nM heregulin-β1, 100 ng/ml Noggin, 5 ng/ml EGF, 5 ng/mlL, FGF-7 (KGF), 20 ng/ml FGF-10, as well as 500 nM A83-01 (TGF-β pathway inhibitor), 500 nM SB202190 (p38 MAPK inhibitor), and 5 μM Y-27632 (ROCK inhibitor). The medium was also supplemented with 1× penicillin/streptomycin and 50 μg/ml primocin to prevent bacterial growth.

#### Tumoroid Treatment and Viability Assays

To evaluate treatment responses, tumoroids were enzymatically dissociated with TrypLE treatment for 5 min. The tumoroids were then diluted in HBSS and then passed through a 100-μm filter. Approximately 3000 to 5000 tumoroid-derived cells/ml were then suspended in 2% Matrigel and distributed at 100 μl per well into 96-well plates (coated with 1.5% agarose to prevent cell adherence). Once tumoroids had reformed (72 h after seeding), drug treatments were applied. For each tumor, we tested two categories of treatment: (i) the conventional chemotherapy regimen that the patient would have received (for tumor 1, this was paclitaxel monotherapy; for tumors 2 and 3, it was the combination of epirubicin, cyclophosphamide, and paclitaxel) and (ii) the proteomic-guided experimental therapy (the combination of targeted drugs selected based on that tumor’s spatial proteomic analysis, *e.g*., cerulenin + sunitinib for tumor 1, bortezomib + dasatinib for tumor 2, paclitaxel + bortezomib for tumor 3). First, key constituent drugs were tested individually for reference (*e.g.,* single-agent paclitaxel, single-agent targeted drug). Tumoroids were treated for 7 days. Drug concentrations were applied in an eight-point dose range (typically 0.01 μM to 100 μM, 10× serial dilutions) to generate dose–response curves. Vehicle controls (*e.g.,* 0.1% DMSO) were included in each plate. All conditions were tested in triplicate wells. After 7 days of treatment, cell viability was assessed using the CellTiter-Glo 3D luminescent assay (Promega). An equal volume (100 μl) of CellTiter-Glo 3D reagent was added to each well, contents were gently mixed, and plates were incubated at room temperature for 25 min. Luminescence was then measured on a microplate reader (Berthold TriStar2 or equivalent). Signal values were normalized to the untreated control wells to calculate % viability. For combined treatments, drugs were administered across a range of seven concentrations: three concentrations 2-fold higher than the IC_50_, the IC_50_, and three concentrations 2-fold lower than the IC_50_.

#### IC_50_ Calculation

For each drug or drug combination, dose–response curves were generated by nonlinear regression using GraphPad Prism 8. The concentration producing 50% growth inhibition (IC_50_) was determined from these fitted curves. Synergy or additivity of drug combinations was qualitatively assessed by comparing the combination IC_50_ to those of the individual agents and by observing the slope/shape of the viability curves.

All experimental data (imaging, proteomic, and viability) were collected and analyzed with an aim to ensure reproducibility. Each key experiment (MALDI MSI clustering, proteomics, tumoroid assays) was performed on at least three biological replicates (the three patient-derived samples) and technical replicates where applicable, with similar results observed. No statistical methods were used to predetermine sample size; the sample size was constrained by available patient tissues. Data are presented with appropriate statistical tests and significance thresholds as described above.

## Results

**Fig. 1:**
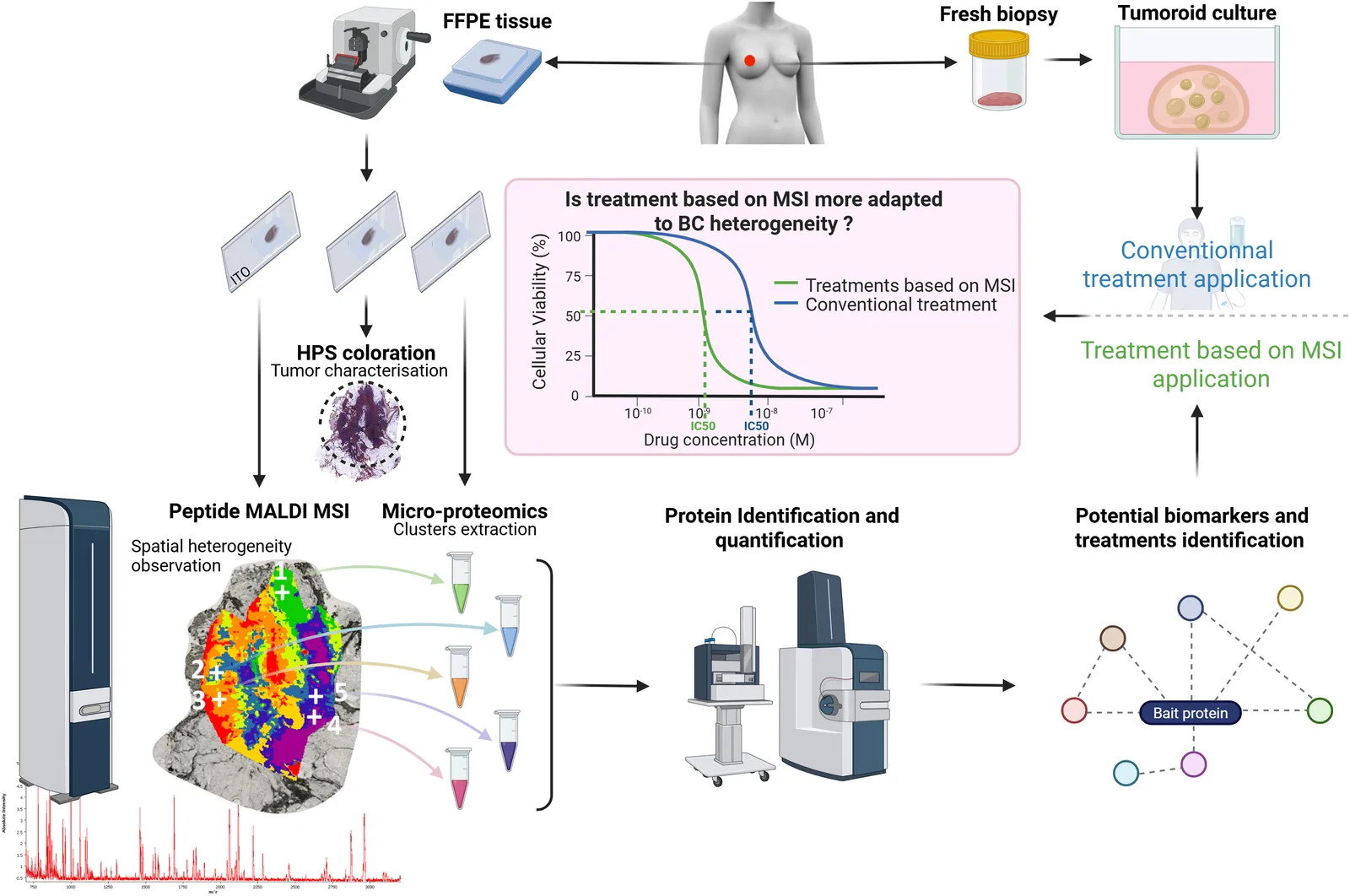
**Theragnostic approach for breast cancer treatment, integrating MSI to address tumor heterogeneity.** The workflow begins with tissue sampling and imaging, followed by MSI analysis to map molecular variations in the tumor. The approach leverages proteomics to identify key protein interactions, leading to more precise, tailored cancer treatments. These data guide personalized treatment strategies. The effectiveness of MSI-based treatments is compared to conventional methods on tumor paired tumoroids, by measuring tumoroid viability. BC, breast cancer; FFPE, formalin-fixed, paraffin-embedded; HPS, hematoxylin-phloxine-saffron; MSI, mass spectrometry imaging.

The results are presented in four sequential steps that follow the experimental workflow (Fig. 1): (i) MALDI MSI segmentation and clone identification across all three tumors; (ii) pathway enrichment analysis and drug target prioritization, with assessment of cellular composition effects; (iii) identification of shared molecular archetypes across patients; and (iv) functional validation of proteomics-guided therapies in PDTs, including cross-validation of treatment specificity.

### Intratumoral Heterogeneity Revealed by MALDI MSI Segmentation

**Fig. 2:**
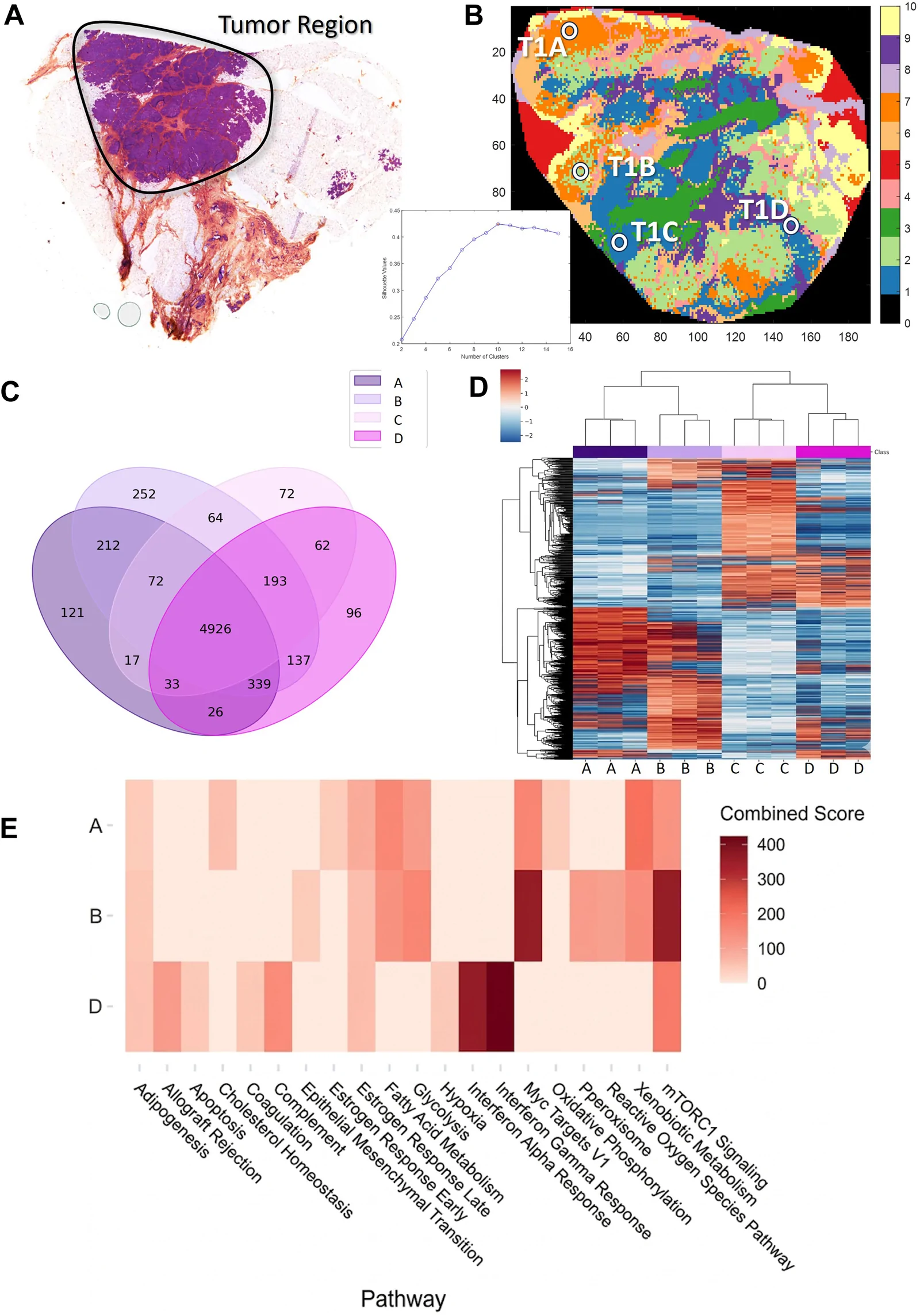
**Spatial and molecular characterization of tumor 1 heterogeneity.***A,panel* (*A*) highlights a region of interest within a tumor section stained with HPS, serving as the anatomical foundation for further analysis. *B,panel* (*B*) maps distinct tumor regions (*A*–*D*) using MALDI MSI spatial clustering, revealing molecularly defined zones. *C*, *panel* (*C*) compares the overlap and uniqueness of molecular features across four classes using a Venn diagram, suggesting both shared and class-specific elements. *D,* panel (*D*) visualizes protein enrichment patterns through ANOVA (*p*-value < 0.01), uncovering overexpressed proteins in *red* and relationships among samples or protein genes. *E*, finally, panel (*E*) displays pathway enrichment scores across the overexpressed proteins from the four classes, identifying biologically significant pathways that may drive tumor behavior in each region. HPS, hematoxylin-phloxine-saffron; MSI, mass spectrometry imaging.

**Fig. 3:**
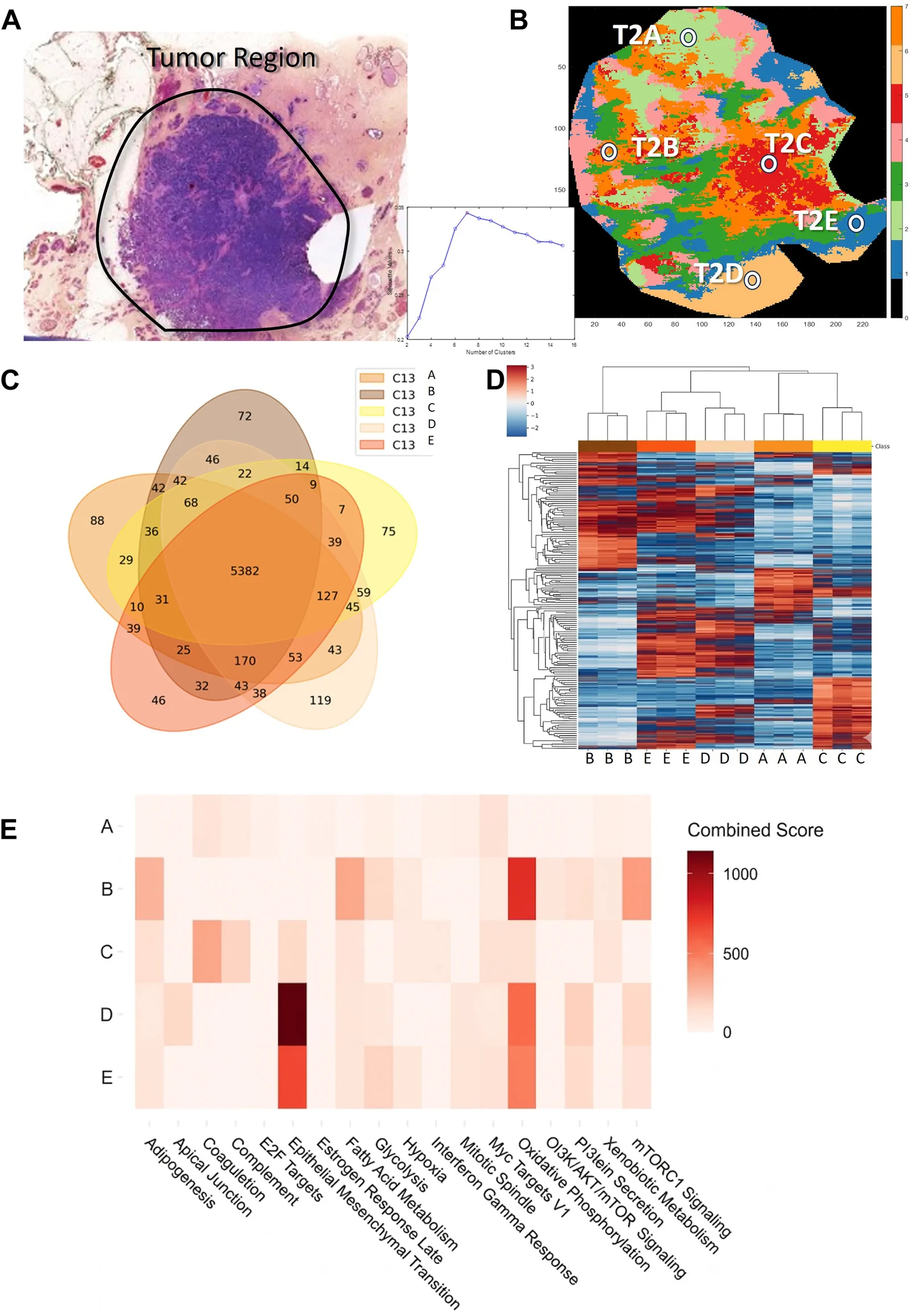
**Spatial and molecular characterization of tumor 2 heterogeneity.***A*, *panel* (*A*) highlights a region of interest within a tumor section stained with HPS, serving as the anatomical foundation for further analysis. *B*, *panel* (*B*) maps distinct tumor regions (*A*–*E*) using MALDI MSI spatial clustering, revealing molecularly defined zones. *C*, *panel* (*C*) compares the overlap and uniqueness of molecular features across five classes using a Venn diagram, suggesting both shared and class-specific elements. *D*, panel (*D*) visualizes protein enrichment patterns through ANOVA (*p*-value < 0.01), uncovering overexpressed proteins in *red* and relationships among samples or protein genes. *E*, finally, panel (*E*) displays pathway enrichment scores across the overexpressed proteins from tumor-specific classes, identifying biologically significant pathways that may drive tumor behavior in each region. HPS, hematoxylin-phloxine-saffron; MSI, mass spectrometry imaging.

**Fig. 4:**
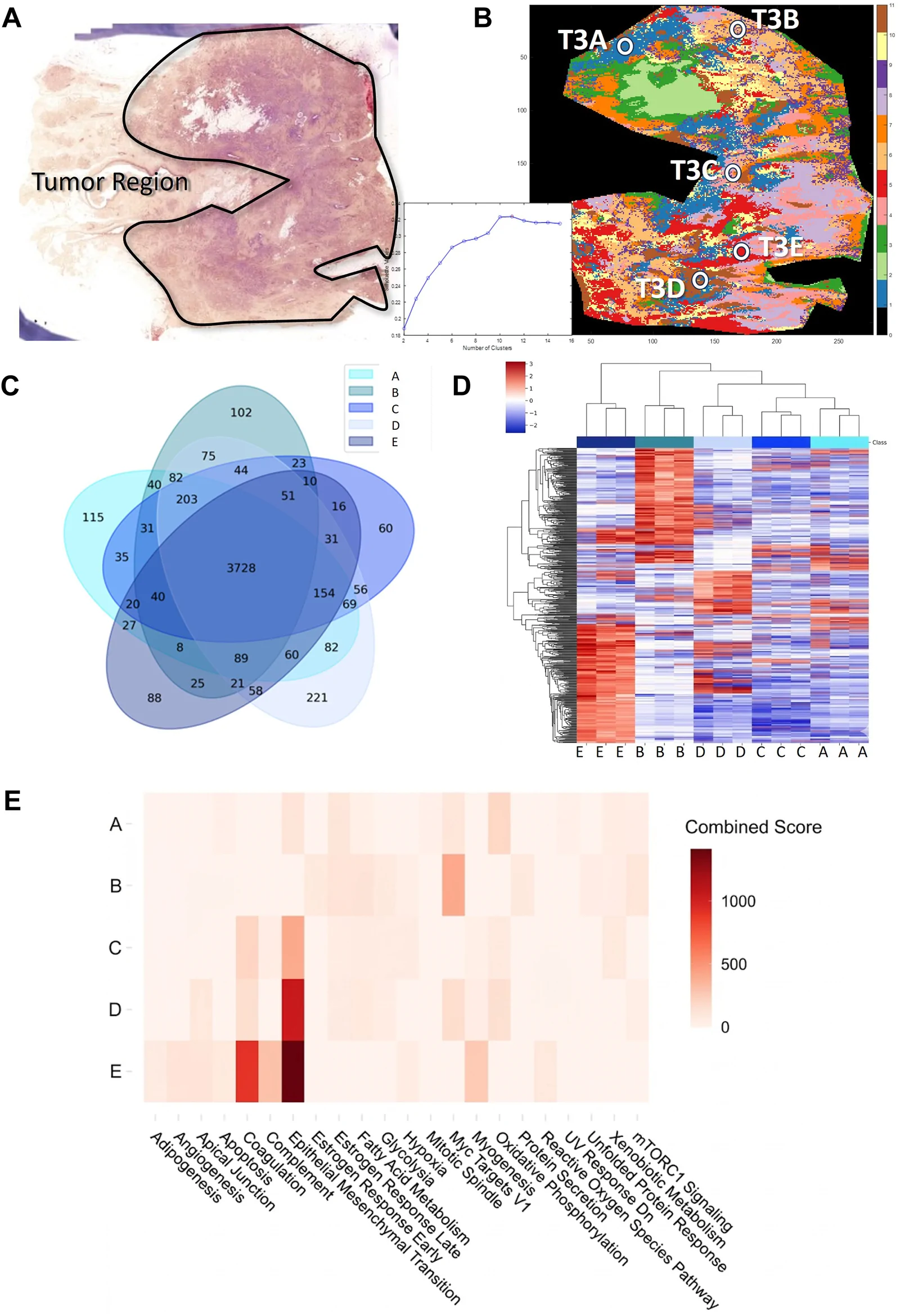
**Spatial and molecular characterization of tumor 3 heterogeneity.***A,panel* (*A*) highlights a region of interest within a tumor section stained with HPS, serving as the anatomical foundation for further analysis. *B*, *panel* (*B*) maps distinct tumor regions (*A*–*E*) using MALDI MSI spatial clustering, revealing molecularly defined zones. *C*, *panel* (*C*) compares the overlap and uniqueness of molecular features across five classes using a Venn diagram, suggesting both shared and class-specific elements. *D*, *panel* (*D*) visualizes protein enrichment patterns through ANOVA (*p*-value < 0.01), uncovering overexpressed proteins in *red* and relationships among samples or protein genes. *E*, finally, panel (*E*) displays pathway enrichment scores across the overexpressed proteins from the five classes, identifying biologically significant pathways that may drive tumor behavior in each region. HPS, hematoxylin-phloxine-saffron; MSI, mass spectrometry imaging.

Unsupervised MALDI MSI analysis of FFPE sections from three luminal breast cancer specimens revealed pronounced intratumoral proteomic heterogeneity in each case. Cluster selection was guided by two complementary criteria: proteomic distinctiveness as assessed by t-SNE cluster separation (Supplementary Fig. S1), and morphological validation by a certified pathologist (N.H.) on the matched HPS-stained sections, which served as the anatomical foundation for the segmentation maps generated for each tumor (Fig. 2*A* for tumor 1, Fig. 3*A* for tumor 2, and Fig. 4*A* for tumor 3). MSI segmentation maps were overlaid on these HPS images to confirm epithelial *versus* stromal cluster identity, and clusters confirmed as purely stromal (desmoplastic stroma, vascular structures) or ambiguous (mixed epithelial/stromal zones) were excluded from spatial proteomic extraction; only regions where pleomorphic nuclei within pink cytoplasmic zones occupied >70 to 80% of the area were classified as epithelium enriched. The optimal cluster number for each tumor was determined by maximizing the average silhouette score over a range of k values, yielding 10, 7, and 11 molecular clusters for tumors 1, 2, and 3, respectively (silhouette curves shown alongside each segmentation map in Figs. 2*B*, 3B, and 4B). The *m/z* distribution and cluster-specific mean spectra underlying these segmentations are provided in Figure S2 (Upset plots and Skyline plots for each tumor), which graphically illustrate the molecular features and their distribution across clusters (resulting *m/z* ions listed in Supplementary MSI data). Not all initial clusters were retained for downstream proteomic analysis: four clusters were retained for tumor 1 (Fig. 2*B*), three epithelial clones (T1-A, T1-B, T1-D) and one stromal region (T1-C), the latter confirmed by Pearson correlation of proteomic profiles (Supplementary Fig. S3*A*); tumor 2 presented a more complex architecture with five distinct clusters (T2-A through T2-E; Fig. 3*B*); and tumor 3 also contained five clusters (T3-A through T3-E; Fig. 4*B*).

For example, the initial k means plus segmentation of tumor 1 identified 10 molecular clusters named C1 to C10 (Figs. 2*B* and S1*A*). Examination of the t-SNE plot showed that clusters C5 (red), C1 (blue), and C6 (light orange) formed a distinct projection in t-SNE space, separated from the main epithelial group, and were subsequently confirmed as nonepithelial regions by histological review. When these clusters were mapped onto the HPS-stained tissue section (Fig. 2*A*), they corresponded to desmoplastic stroma, fibroblast rich regions, and peritumoral adipose tissue. For this reason, they were excluded from the subsequent proteomic analysis. Three other clusters, C2 (light green), C7 (orange), and C8 (purple), occupied intermediate positions in the t-SNE plot and showed unclear histological features. Following review by a pathologist, these clusters were identified as transitional regions between stroma and epithelium and were also excluded from further analysis. The four remaining clusters, renamed T1 A (orange), T1 B (light green), T1 C (blue), and T1 D (purple), were retained for downstream analyses. Among them, T1 C was later confirmed as a stromal region through Pearson correlation analysis of spatial proteomic profiles (Supplementary Fig. S3*A*). This cluster showed a lower correlation with the three epithelial clones, with correlation values ranging from 0.68 to 0.69, whereas the epithelial clones T1 A, T1 B, and T1 D showed very high pairwise correlations ranging from 0.97 to 0.98. These results indicate that T1 C possesses a clearly distinct proteomic composition compared with the epithelial regions.

### Pathway Enrichment, Cellular Composition Assessment, and Drug Target Prioritization

Differential protein expression across clusters was assessed by ANOVA (*p* < 0.01) for each tumor: 1128 of 3810 quantifiable proteins were significantly differentially expressed in tumor 1 (Supplementary Spreadsheets 2 and 3); 2031 of 4221 in tumor 2 (Supplementary Spreadsheets 4 and 5); and 976 of ∼2600 in tumor 3 (Supplementary Spreadsheets 6 and 7). The proteins shared between clones and those unique to each clone are summarized by Venn analysis for each tumor (Fig. 2*C*, 3C, and 4C). Hierarchical clustering of these proteins revealed clone-specific signatures alongside a shared tumor protein core in each specimen (Fig. 2*D*, 3D, and 4D).

Pathway enrichments were derived by over-representation analysis applied exclusively to proteins significantly differentially expressed among clones (ANOVA, *p* < 0.01), so that enrichment scores reflect interclone biological differences rather than absolute pathway abundance. To systematically evaluate the potential contribution of nontumor cell populations, we quantified established stromal, immune, and vascular marker proteins across all clusters in parallel with the pathway analyses.

For clinical reference, each patient had an assigned standard-of-care chemotherapy regimen: paclitaxel monotherapy for tumor 1 (luminal B); epirubicin, cyclophosphamide, and paclitaxel (E/C/P) for tumors 2 and 3. These regimens served as comparators in the subsequent tumoroid validation experiments.

#### Tumor 1: Metabolic and Hormonal Vulnerabilities in Three Epithelial Clones

Pathway enrichment analysis of the three epithelial clones revealed three distinct biological identities (Fig. 2*E*). Clone T1-A was most enriched for estrogen response early and late, fatty acid metabolism, glycolysis, and mTORC1 signaling (consistent with a hormone-driven, anabolic phenotype) with FASN and ESR1 among its most elevated proteins (Supplementary Fig. S3*B*). Clone T1-B showed the strongest enrichment for oxidative phosphorylation, reactive oxygen species pathway, xenobiotic metabolism, and peroxisome signatures, supported by elevated GPX1 (*p* = 0.035) and PRDX1 (*p* = 0.048; Supplementary Fig. S3*B*). Clone T1-D was most enriched for hypoxia, interferon alpha response, and interferon gamma response, with the highest expression of IFIT1 (*p* = 0.035), IFIT2 (ns, *p* = 0.070), MX1 (*p* = 0.035), MX2 (*p* = 0.036), ISG15 (*p* = 0.035), and STAT1 (*p* = 0.035) among epithelial clones (Supplementary Fig. S3*B*).

Cellular composition analysis (Supplementary Fig. S4) confirmed the stromal identity of T1-C via elevated VIM (*p* = 0.035), FN1 (*p* = 0.035), and COL1A1 (*p* = 0.035), all significantly higher than in epithelial clones. T1-D showed intermediate VIM and FN1 levels, significantly elevated relative to T1-A and T1-B, suggesting possible stromal adjacency. Among T-cell-associated markers, THY1 was highest in T1-C (*p* = 0.035), PTPRC peaked in T1-C and T1-D (*p* = 0.035), CD44 showed no significant variation (*p* = 0.58), and GATA3 was significantly lower in T1-C and T1-D than in T1-A and T1-B (*p* = 0.044). Among macrophage-associated markers, CD14 (*p* = 0.035), ITGAM (*p* = 0.035), ITGAX (*p* = 0.035), ITGB2 (*p* = 0.035), and LYZ (*p* = 0.035) were all significantly elevated in T1-D relative to T1-A and T1-B, while GPNMB (*p* = 0.035) and APOE (*p* = 0.035) were highest in T1-C. HLA-DRA (*p* = 0.035) was most elevated in T1-D. AIF1 (*p* = 0.14), LGMN (*p* = 0.11), and CTSB (*p* = 0.11) did not reach significance. Canonical macrophage markers CD68 and CD163 were absent from the preprocessed dataset, a limitation of MALDI MSI data-independent acquisition for low-abundance immune proteins.

These data indicate that T1-A and T1-B showed the lowest immune and stromal marker levels among all clusters, supporting a tumor cell-intrinsic origin for their metabolic and redox enrichments. T1-D contains the highest myeloid marker co-enrichment among epithelial clones, raising the possibility that its interferon signatures are partially driven by infiltrating macrophages; however, its dominant hypoxia enrichment (the strongest signal in Fig. 2*E*) and ISG induction (IFIT1, MX1, and MX2) are not macrophage-specific programs and can be induced in tumor cells by cytosolic nucleic acid sensing, suggesting a mixed contribution.

Based on these profiles, cerulenin (a FASN inhibitor) (29) was selected to target the lipid and estrogen-driven metabolism of T1-A, where FASN was most elevated among epithelial clones (Supplementary Fig. S3*B*). Sunitinib, which targets VEGFR2, PDGFRB, and CSNK2A1, was selected to address the hypoxia-associated signaling (30) of T1-D; although CSNK2A1 interclone differences did not reach significance (*p* = 0.14), its absolute expression was detectable in T1-D at levels supportive of kinase dependency (Supplementary Fig. S3*B*). Paclitaxel was excluded based on elevated expression of resistance markers: EDIL3 (ns *p* = 0.060, highest in T1-B) and CapG (ns *p* = 0.060, highest in T1-B and T1-D) are elevated in resistant clones (Supplementary Fig. S3*B*); CA12 (ns *p* = 0.14) was highest in T1-D (31); and PGK1 (ns *p* = 0.14), though not specifically elevated in T1-B/D, showed broad expression across all clones consistent with a permissive resistance background (32, 33, 34, 35, 36). The cerulenin + sunitinib combination was therefore predicted to outperform paclitaxel, whose mechanism of microtubule stabilization does not address the metabolic or hypoxia-driven vulnerabilities identified here (31).

#### Tumor 2: Proteostatic Stress and EMT Vulnerabilities Across Five Clones

Pathway enrichment analysis (Fig. 3*E*, Supplementary Fig. S5*B*) revealed five molecularly distinct clone identities. EMT was the most prominent pathway overall, peaking in T2-D and elevated in T2-E, consistent with elevated SPARC, MMP2, and MMP14. Myc Targets V1 was most enriched in T2-B, alongside elevated ENO1 and PKM. Interferon gamma response was enriched in T2-C and T2-D, with GBP1 and GBP2 highest in T2-D, while complement proteins C3, C1R, and CFB peaked in T2-C. PI3K/AKT/mTOR signaling was moderately enriched in T2-D and T2-E, consistent with significantly elevated PIK3R1 (*p* = 0.020), while RPS6KB1 showed a nonsignificant trend (*p* = 0.055; Supplementary Fig. S5*B*). Glycolysis enrichment was observed in T2-B and T2-D (elevated LDHA and ENO1).

Cellular composition analysis (Supplementary Fig. S6) identified T2-C as the cluster most heavily influenced by nontumor cell infiltration. Among macrophage-associated markers, ITGAM (*p* = 0.033), ITGB2 (*p* = 0.033), LYZ (*p* = 0.031), GPNMB (*p* = 0.031), CTSS (*p* = 0.033), CTSB (*p* = 0.031), HLA-DRA (*p* = 0.031), and HLA-DPB1 (*p* = 0.031) were all most elevated in T2-C. Among T-cell-associated markers, PTPRC was most elevated in T2-C (*p* = 0.045), consistent with leucocyte infiltration, while THY1 (*p* = 0.045) and CD44 (*p* = 0.045) were highest in T2-D and T2-E. GATA3 did not reach significance (*p* = 0.086). APOE (*p* = 0.031) was highest in T2-A. For endothelial markers, VWF (*p* = 0.018) and MCAM (*p* = 0.027) were most elevated in T2-C, indicating a vascular component. These data collectively identify T2-C as the cluster most influenced by microenvironmental composition: its interferon and complement enrichments should be interpreted primarily as environmental signals rather than tumor cell-intrinsic biology. By contrast, the EMT signatures of T2-D and T2-E and the proliferative signature of T2-B show lower immune marker co-enrichment and are more likely tumor intrinsic.

Bortezomib was selected to target clones with elevated proteostatic burden (37, 38, 39), particularly T2-D (where HSP90B1 was most elevated; *p* = 0.020) and T2-C (intermediate HSP90B1; ACAT1 *p* = 0.020 and ALDH1A1 *p* = 0.020 most elevated in T2-C; ANPEP *p* = 0.020 also highest in T2-C; Supplementary Fig. S5*B*). Dasatinib was directed at T2-B, T2-D, and T2-E, where PI3K/AKT/mTOR signaling was elevated (PIK3R1 *p* = 0.020), CASP7 was most expressed in T2-D and T2-E (*p* = 0.020), and CA12 peaked in T2-E (*p* = 0.050; Supplementary Fig. S5*B*) (40, 41, 42). Together, bortezomib and dasatinib address complementary vulnerabilities: proteostatic stress in the metabolically burdened clusters and kinase-driven signaling in the proliferative and invasive ones.

#### Tumor 3: EMT and Mesenchymal Vulnerabilities Dominate the Proteomic Landscape

Pathway enrichment analysis (Fig. 4*E*, Supplementary Fig. S7*B*) showed a dominant EMT and coagulation/complement gradient peaking in T3-D and T3-E. T3-B showed myogenesis enrichment and elevated glycolytic markers (ALDOA, GAPDH). UPR, Myc targets, mTORC1, and oxidative phosphorylation enrichments were uniformly low across all clusters.

At the protein level, coagulation factors FGA, FGB, FGG (all *p* = 0.022), F2 (*p* = 0.022), and SERPINC1 (*p* = 0.022), alongside complement proteins C3 (*p* = 0.022), C1S (*p* = 0.023), and CFB (*p* = 0.022), were all most elevated in T3-E (Supplementary Fig. S8); C1R showed a nonsignificant trend (*p* = 0.060) and is not included as a statistically supported marker. Cellular composition analysis confirmed that stromal markers COL1A1 (*p* = 0.022), BGN (*p* = 0.022), DCN (*p* = 0.022), and LUM (*p* = 0.020) were most elevated in T3-C and T3-E, co-localizing with the strongest coagulation and complement signals. Vascular markers VWF (*p* = 0.026) and DES (*p* = 0.040) were similarly elevated in T3-E, and LYZ (*p* = 0.022) peaked in T3-C and T3-E (Supplementary Fig. S8). These data indicate that the complement and coagulation enrichments of T3-E are at least partially driven by stromal and vascular cell colocalization.

Paclitaxel was selected based on mitotic spindle pathway enrichment in T3-B (Fig. 4*E*) and expression of tubulin isoforms across clusters (43): TUBB was significantly elevated in T3-B (*p* = 0.045), and TUBA4A was significantly differentially expressed (*p* = 0.038), with highest levels in T3-B and T3-A (Supplementary Fig. S7*B*). TUBB4A (*p* = 0.19, ns) and TUBB2A (*p* = 0.077, ns) showed nonsignificant trends and are noted as supporting context rather than primary statistical evidence. Bortezomib was selected to address the protein synthesis and secretory burden in mesenchymal clones T3-D and T3-E, where high expression of LAMB1 (*p* = 0.038), LAMC1 (*p* = 0.038), SPARC (*p* = 0.038), COL4A2 (*p* = 0.038), and FN1 (*p* = 0.020) implies elevated proteasomal dependency, supported by significantly elevated chaperones CANX (*p* = 0.038) in T3-D and HSPA8 (*p* = 0.038) in T3-B (Supplementary Fig. S7*B*); HSP90B1 showed a nonsignificant trend (*p* = 0.054). Together, paclitaxel targets the proliferative clones and bortezomib, the secretory-burdened mesenchymal clones, covering the principal axes of heterogeneity identified in tumor 3.

Having characterized clone-specific pathway vulnerabilities in each individual tumor and derived patient-specific therapeutic hypotheses, we next performed an integrated cross-patient analysis to determine whether recurring molecular programs underlie these intertumor differences.

### Cross-Patient Analysis Identifies Three Recurring Molecular Archetypes

**Fig. 5:**
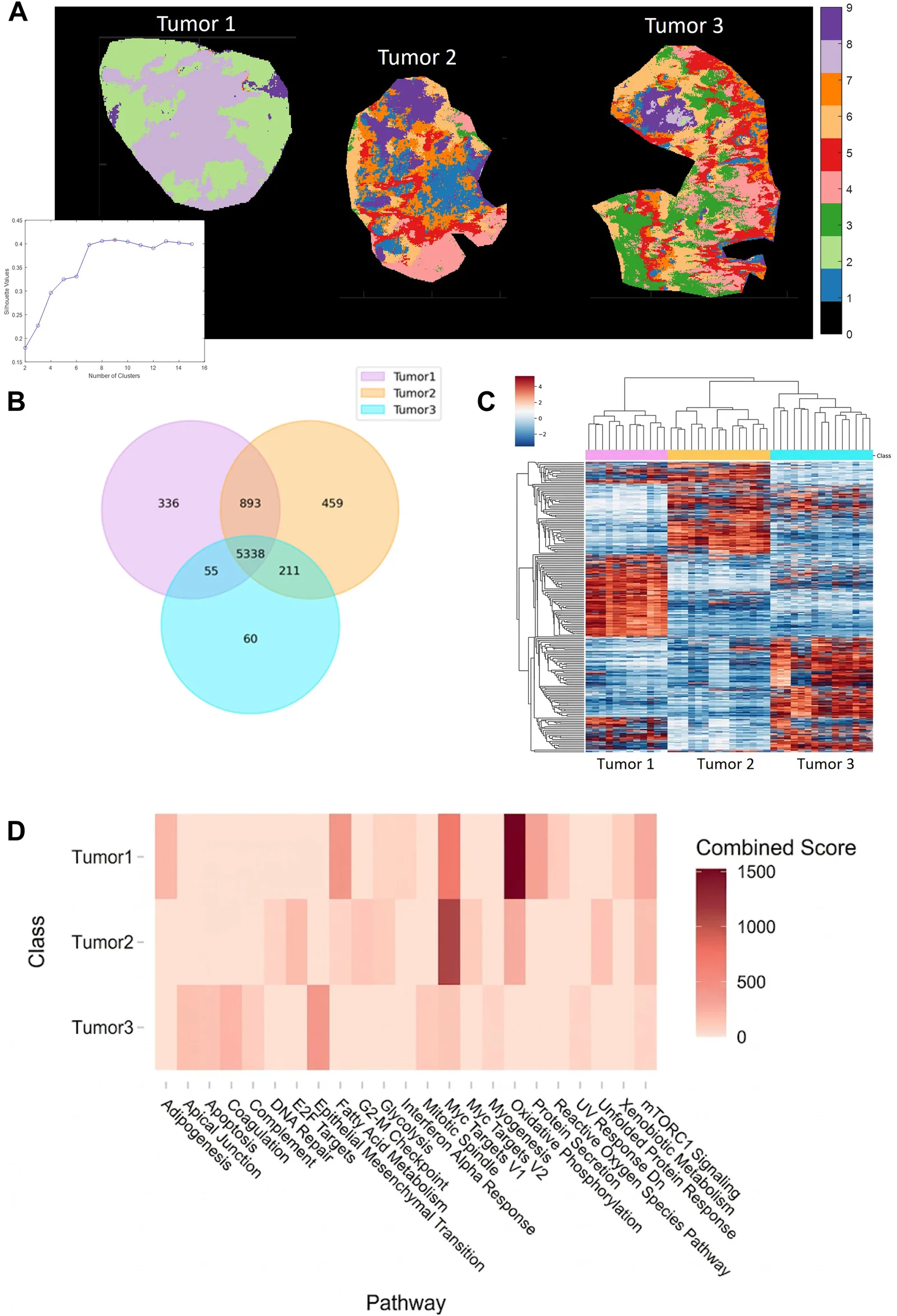
**Spatial and molecular characterization of intertumor heterogeneity.***A, B,panel* (*A*) MALDI images cosegmentation with nine clusters following silhouette criterion, spatial proteomic analysis represented through (*B*) compares the overlap and uniqueness of molecular features across three tumors using a Venn diagram, suggesting both shared and class-specific elements. *C*, *panel* (*C*) visualizes protein enrichment patterns through ANOVA (*p*-value < 0.01), uncovering overexpressed proteins in *red* and relationships among samples or protein genes. *D*, finally, *panel* (*D*) displays pathway enrichment scores across the overexpressed proteins from the three tumors, identifying biologically significant pathways that may drive tumor behavior in each tumor.

Integrated co-clustering of MALDI MSI proteomic profiles from all three tumors produced a composite segmentation with nine clusters (Fig. 5*A*), grouping tissue regions from different patients by molecular similarity independent of tumor of origin. Cohen's Kappa similarity analysis confirmed partial but meaningful overlap in proteomic composition between tumors (tumor 1 *versus* tumor 2 κ = 0.57; tumor 1 *versus* tumor 3 κ = 0.55; tumor 2 *versus* tumor 3 κ = 0.62; Supplementary Fig. S9*A*). A Venn diagram (Fig. 5*B*) showed that out of 7352 proteins identified across all three tumors, 5338 were shared, with 336 unique to tumor 1, 459 unique to tumor 2, and 60 unique to tumor 3; 893 proteins were shared between tumors 1 and 2, and 211 between tumors 2 and 3 exclusively, confirming that tumors 2 and 3 share the largest interpatient proteome overlap. ANOVA across tumors (*p* < 0.01) identified 2748 differentially expressed proteins, whose hierarchical clustering (Fig. 5*C*) revealed tumor-specific expression blocks alongside a shared core program.

The pathway enrichment heatmap across all tumors (Fig. 5*D*) reveals three recurring molecular profiles. The first, tumor 1, is characterized by enrichment of estrogen response, fatty acid metabolism, glycolysis, MYC/E2F activity, and mTORC1 signaling. The second, most prominent in tumor 2, is associated with proteostatic stress, oxidative stress response, and interferon signaling with partial EMT features. The third, dominant in tumor 3, is defined by EMT, coagulation, and extracellular matrix remodeling. The heatmap (Supplementary Fig. S9*C*) shows that across all individual clones from all three tumors, the mitotic spindle and Myc targets V1 pathways show consistently elevated scores in proliferative clusters (T1-A, T1-B, T2-B, T3-B) while epithelial mesenchymal transition, coagulation, and E2F targets show more spatially restricted enrichment in mesenchymal clusters (T2-D, T2-E, T3-D, T3-E).

To directly link these archetypes to the therapeutic choices made for each patient, we examined the expression of primary drug target proteins across all three tumors (Supplementary Fig. S10). FASN (*p* = 0.035) and ACLY (*p* = 0.002) (targets of cerulenin) were most elevated in tumor 1. Proteasome subunits PSMB1 (*p* = 1.86e-06), PSMB5 (*p* = 1.76e-05), PSMB6 (*p* = 0.035), PSMA1 (*p* = 1.61e-05), and PSMD1 (*p* = 2.71e-07) (targeted by bortezomib) were all most elevated in tumor 1 and significantly lower in tumors 2 and 3. CSNK2A1 (inhibited by sunitinib) was highest in tumor 2 and significantly lower in tumor 3 (*p* = 0.0004; Supplementary Fig. S10). Tubulin isoforms TUBB (*p* = 1.86e-08) and TUBB4A (*p* = 1.35e-06) were most elevated in tumor 2, while TUBB2A (*p* = 0.0005) was most elevated in tumor 1; TUBA4A (*p* = 0.037) and TUBB4B (*p* = 0.041) showed significant intertumor variation. This cross-tumor target expression map provides the molecular rationale for why each combination was predicted to be most potent in its matched tumor model. These shared molecular profiles should be interpreted as functional states rather than discrete tumor classes and are presented here as hypothesis-generating observations.

With patient-specific drug combinations derived from spatial pathway analysis and cross-patient archetypes characterized, we proceeded to functionally test each proteomics-guided regimen against the corresponding standard-of-care in matched PDTs.

### Functional Validation in PDTs

Cosine similarity analysis (Supplementary Fig. S11) confirmed that tumoroid proteomic profiles shared substantial concordance with their matched tumor epithelial clusters: for tumor 1, similarity scores between tumoroid 1 and the epithelial clones T1-A, T1-B, and T1-D ranged from 0.62 to 0.66, while T1-C (stromal) showed a lower score of 0.39, consistent with its distinct cellular identity; for tumor 2, tumoroid 2 showed similarity scores of 0.59 to 0.72 with all five clusters; and for tumor 3, tumoroid 3 showed scores of 0.54 to 0.70 with the five retained clusters. Key drug target proteins identified in the primary tumor clones were confirmed present in the tumoroids prior to treatment (Supplementary Figs. S12, S13; Supplementary Spreadsheets 10 and 11), supporting the relevance of this *ex vivo* system for testing proteomics-guided strategies.

### Proteomics-Guided Combinations Outperform Standard-of-Care in Matched Tumoroids

Across the three tumoroid models, the proteomics-guided drug combinations consistently outperformed the corresponding standard-of-care regimen, primarily by selecting agents with demonstrated single-agent potency and excluding agents to which each tumoroid was insensitive. The degree of combination synergy varied by tumor model and is reported below.

#### Tumor 1 Tumoroid: Paclitaxel Resistance Confirmed; Cerulenin + Sunitinib Effective

**Fig. 6:**
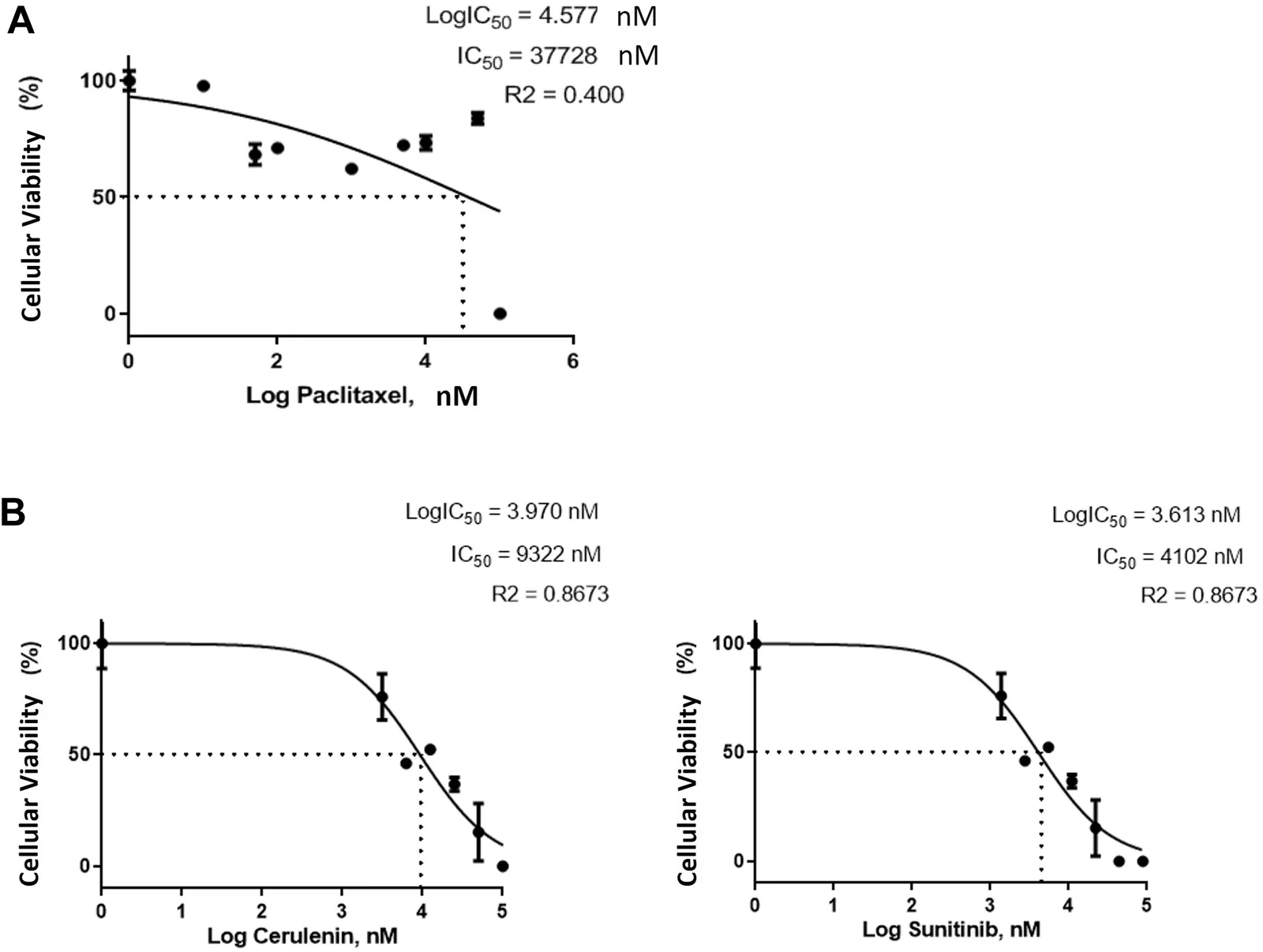
**Dose–response curves and IC_50_ values obtained from viability assays performed on tumor 1-derived tumoroids treated with various compounds.***A, B*, paclitaxel (*A*) and (*B*) the combination of cerulenin and sunitinib.

Tumor 1 tumoroids treated with paclitaxel showed minimal response, with an IC_50_ of 37,728 nM (LogIC_50_=4.577; R^2^ = 0.40) and a shallow dose–response curve (Fig. 6*A*), consistent with near-complete resistance. The proteomic basis for this resistance was directly confirmed in the tumoroid dataset: paclitaxel resistance markers EDIL3 and CapG (elevated in T1-B and T1-D of the primary tumor; Supplementary Fig. S3*B*), CA12 (highest in T1-D), and PGK1 (broad expression across all clones) were all detected at high levels in tumor 1 tumoroids prior to treatment (Supplementary Fig. S13). By contrast, cerulenin and sunitinib as single agents yielded IC_50_ values of 25,188 nM (LogIC_50_=4.40; R^2^ = 0.54) and 11,460 nM (LogIC_50_=4.059; R^2^ = 0.87), respectively (Supplementary Fig. S14*A*), both substantially lower than paclitaxel. In combination (Fig. 6*B*), IC_50_ values decreased further to 9322 nM for cerulenin (LogIC_50_=3.970; R^2^ = 0.87) and 4102 nM for sunitinib (LogIC_50_=3.613; R^2^ = 0.87), representing approximately 2.7- and 2.8-fold reductions relative to single-agent use, with steeper dose–response curves indicative of improved killing efficiency.

#### Tumor 2 Tumoroid: Bortezomib and Dasatinib Superior to E/C/P; Combination Improves Potency

**Fig. 7:**
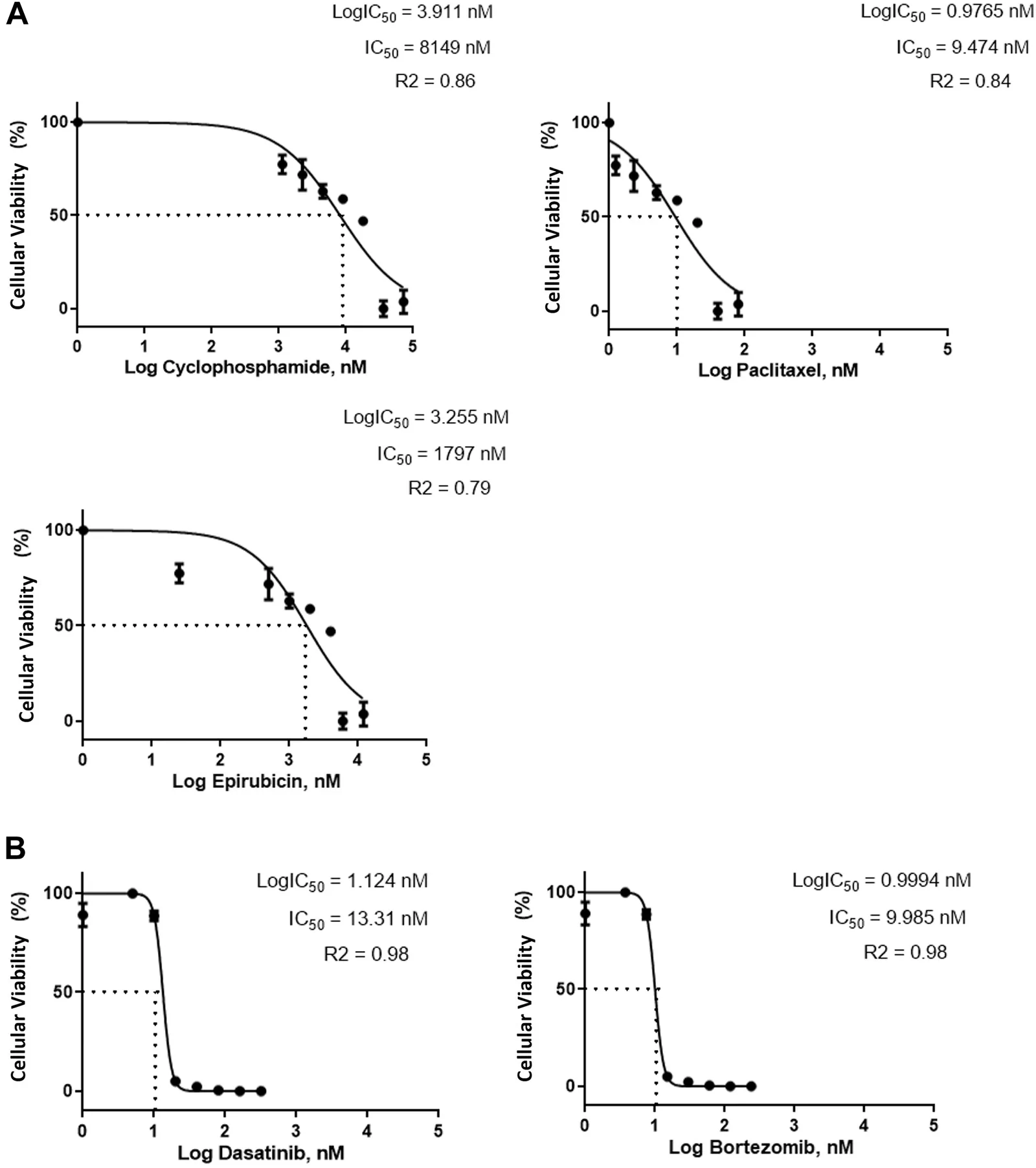
**Dose–response curves and IC_50_ values obtained from viability assays performed on tumor 2-derived tumoroids treated with various compounds.***A, B,* combination of paclitaxel, epirubicin, and cyclophosphamide (*A*) and (*B*) the combination of dasatinib and bortezomib.

Single-agent testing in tumor 2 tumoroids (Supplementary Fig. S15*A*) demonstrated that bortezomib (IC_50_=29.46 nM; Log IC_50_=1.469; R^2^ = 0.97) and dasatinib (IC_50_=37.56 nM; LogIC_50_=1.5; R^2^ = 0.99) were markedly more potent than the E/C/P components: epirubicin (IC_50_=2079 nM; R^2^ = 0.98), cyclophosphamide (IC_50_=9537 nM; R^2^ = 0.11), and paclitaxel (IC_50_=13.18 nM; R^2^ = 0.70). The E/C/P combination components (Fig. 7*A*) showed no improvement over single-agent values: cyclophosphamide IC_50_ remained at 8149 nM (R^2^ = 0.86), epirubicin at 1797 nM (R^2^ = 0.79), and paclitaxel at 9.474 nM (R^2^ = 0.84). These data support preferential activity of the proteomics-guided combination, though formal pharmacological synergy was not established. The very low R^2^ for cyclophosphamide (0.11) indicates a poorly fitted dose–response curve, consistent with a weak and irregular response across concentrations. In the combination setting (Fig. 7*B*), dasatinib IC_50_ decreased to 13.31 nM (Log IC_50_=1.124; R^2^ = 0.98) and bortezomib to 9.985 nM (Log IC_50_=0.999; R^2^ = 0.98), representing approximately 2.8- and 3.0-fold improvements.

#### Tumor 3 Tumoroid: Paclitaxel + Bortezomib Superior to E/C/P; Limited Combination Synergy

**Fig. 8:**
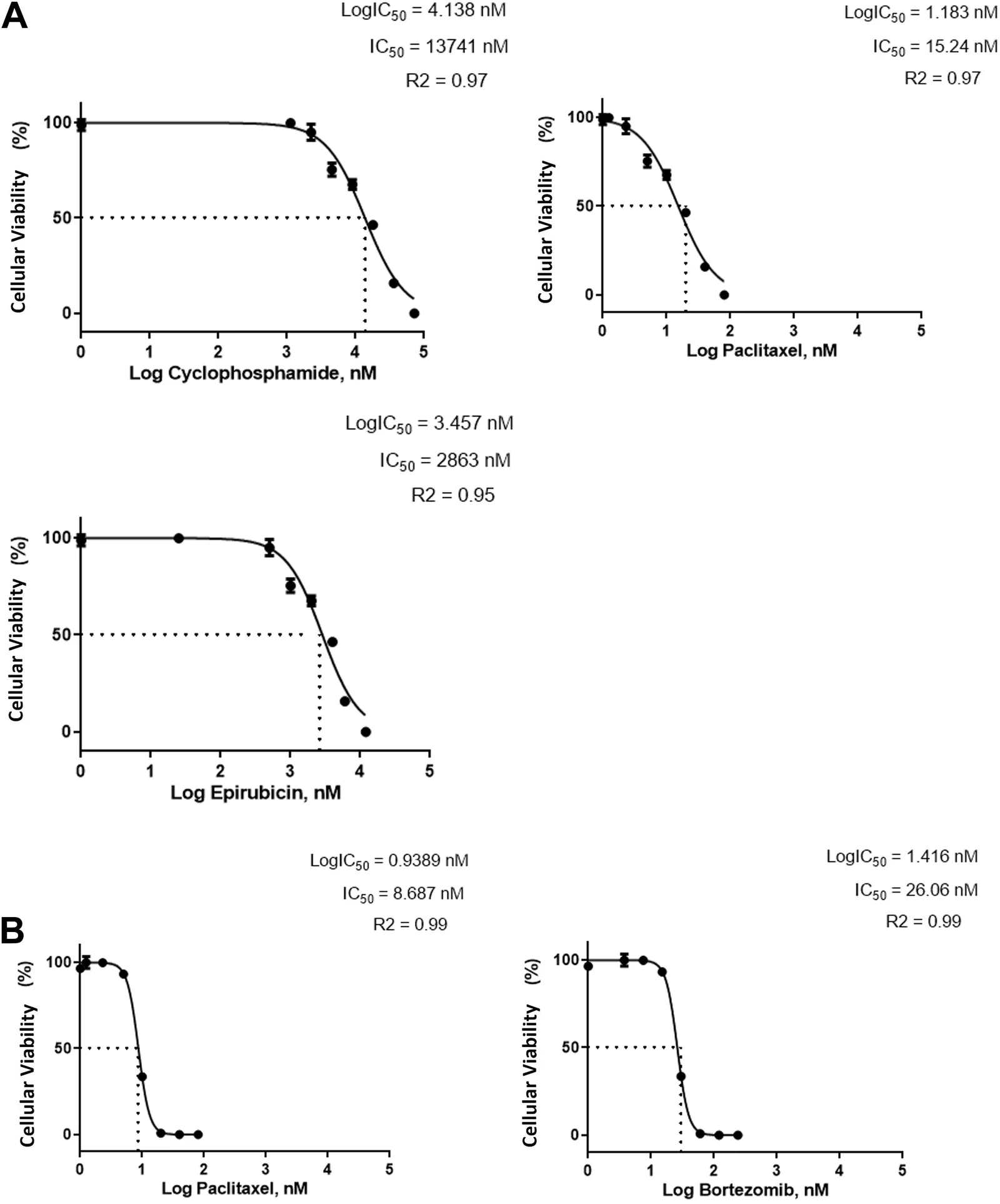
**Dose–response curves and IC_50_ values obtained from viability assays performed on tumor 3-derived tumoroids treated with various compounds**. *A*, *B,* combination of paclitaxel, epirubicin and cyclophosphamide (*A*) and (*B*) the combination of paclitaxel and bortezomib.

In tumor 3 tumoroids, single-agent testing (Supplementary Fig. S16*A*) confirmed that paclitaxel (IC_50_=10.12 nM; Log IC_50_=1.005; R^2^ = 0.80) and bortezomib (IC_50_=28.58 nM; Log IC_50_=1.456; R^2^ = 0.99) were the most potent agents. Epirubicin showed moderate activity (IC_50_=2286 nM; R^2^ = 0.99), and cyclophosphamide was largely ineffective (IC_50_=9108 nM; R^2^ = 0.14). In the proteomics-guided combination (Fig. 8), paclitaxel IC_50_ was 8.687 nM (R^2^ = 0.99) and bortezomib 26.06 nM (R^2^ = 0.99). These values are comparable to single-agent IC_50_ values, indicating that the combination did not demonstrate clear additive or synergistic activity beyond what either agent achieved individually, an acknowledged limitation. The data support the superiority of each individual agent over E/C/P components (particularly the exclusion of cyclophosphamide) but do not unambiguously demonstrate true combination synergy for tumor 3.

### Cross-Validation Confirms Tumor-of-Origin Specificity for Tumor 1; Partial for Tumors 2 to 3

To evaluate the tumor-specificity of each proteomics-guided combination, each treatment was subsequently tested across all three tumoroid models at concentrations standardized to the IC_50_ determined in the matched model (Supplementary Figs. S17, S18 and S19).

The cerulenin + sunitinib combination (tumor 1-derived; Supplementary Fig. S17) showed the clearest tumor-of-origin specificity: dose–response curves in tumor 2 and tumor 3 tumoroids were substantially right-shifted and flatter, with both drugs requiring considerably higher concentrations to achieve equivalent viability reduction, consistent with the lower FASN and ACLY expression in tumors 2 and 3 (Supplementary Fig. S10). IC_50_ fold-changes relative to tumor 1 exceeded 5-fold in nonmatching models.

The dasatinib + bortezomib combination (tumor 2-derived; Supplementary Fig. S18) showed more modest specificity (∼1.3–2-fold IC_50_ shifts in nonmatching models), reflecting the broader cellular relevance of proteasome function and kinase signaling across tumor types. As noted above, the combination did not clearly outperform either drug alone in tumor 2, which limits the strength of the specificity conclusion.

The paclitaxel + bortezomib combination (tumor 3-derived; Supplementary Fig. S19) showed consistently high potency across all three tumoroid models (∼1.3–2-fold IC_50_ fold-changes), as both microtubule dynamics and proteasome function are fundamental cellular processes. Notably, bortezomib cotreatment appeared to partially overcome paclitaxel resistance in tumor 1 tumoroids (IC_50_ ∼6 nM *versus* 37,728 nM for paclitaxel alone), though this was not a primary prediction of the proteomics-guided framework and warrants prospective investigation.

Together, these cross-validation data provide partial support for the proteomics-guided approach. Tumor-of-origin specificity was clearest for tumor 1 (>5-fold shifts in nonmatching models). For tumors 2 and 3, the proteomics-guided combinations outperformed conventional regimens and identified effective single agents, but tumor-of-origin specificity was limited (∼1.3–2-fold), and true combination synergy was not demonstrated. Demonstrating robust tumor-specific targeting will require additional models beyond the scope of the present study.

### Consistency of In Vitro Drug Responses With Clinical Pharmacology

Response curves used to determine the IC50 values for each treatment were established according to published literature on breast cancer cell therapies. Overall, the concentrations used in this study are clinically relevant, as when tumoroids responded to a given treatment, the corresponding IC50 values were generally within the range reported in human pharmacokinetic studies.

For example, paclitaxel is typically effective in patients at concentrations ranging from 80 to 280 nM (31). When tumor 2 responded well to paclitaxel, the IC50 was 13.18 nM, and a similar response was observed for tumor 3, with an IC50 of 10.12 nM. In contrast, tumor 1, which was resistant to paclitaxel, exhibited a much higher IC50 of 37,728 nM. Similar trends were observed for epirubicin (clinically administered at 9.2–16.6 μM) (44) and cyclophosphamide (180–215 μM), where dose–response curves remained within clinically relevant ranges, even in resistant tumoroids.

For treatments currently under clinical evaluation, bortezomib is administered to patients at 0.1 to 0.3 μM (37, 39). Tumoroids 2 and 3 responded well to this drug, with IC_50_ values around 29 nM. Likewise, dasatinib is recommended at patient doses of 0.12 to 0.22 μM (41, 42), and tumoroid 2 showed a strong response with an IC50 of 29.46 nM.

However, some discrepancies were observed. For instance, sunitinib was used at a higher concentration in our experiments (11.5 μM) compared to reported patient plasma concentrations (0.15–0.25 μM) (31, 45). It should be noted that sunitinib is still under clinical investigation, and drug concentrations administered to patients do not directly reflect intratumoral drug levels. Furthermore, in this study, treatments were applied to tumoroids for only 7 days, whereas patients typically receive therapy over much longer periods. Therefore, direct comparisons between *in vitro* and *in vivo* drug concentrations should be interpreted with caution, as *in vitro* systems do not account for pharmacokinetic constraints, systemic toxicity, or adverse effects observed in patients.

## Discussion

In this study, we developed and applied an integrated spatial proteomics pipeline combining MALDI mass spectrometry imaging and PDT drug testing to characterize intratumoral heterogeneity and guide personalized therapy selection in luminal breast cancer. Analysis of three FFPE tumor specimens revealed that each tumor harbored multiple molecularly distinct clonal subpopulations defined by unique protein expression signatures and pathway activations, a level of heterogeneity invisible to conventional histopathological subtyping or bulk genomic profiling. These spatially resolved molecular profiles were used to select multi-targeted drug combinations, which were then evaluated in matched PDTs alongside standard-of-care regimens. In two of the three cases, proteomics-guided combinations outperformed standard regimens, primarily by identifying agents to which each tumor was intrinsically sensitive and by excluding agents to which resistance was proteomolecularly predicted. Taken together, the present work provides proof-of-concept for a pipeline that moves from tissue imaging and clone-specific characterization to functionally validated therapeutic selection.

The three tumors examined here displayed markedly distinct dominant phenotypes despite sharing a luminal breast cancer classification, illustrating the inadequacy of subtype-level treatment assignment for capturing actionable intratumoral diversity. Tumor 1 was characterized by a hormone-driven, lipid-anabolic phenotype, with estrogen response, fatty acid metabolism, glycolysis, and mTORC1 signaling enriched in its dominant epithelial clone T1-A and elevated FASN, ESR1, and ACACA as central effectors. Clone T1-B presented a distinct oxidative stress program enriched for oxidative phosphorylation and reactive oxygen species, supported by elevated GPX1 and PRDX1. Clone T1-D showed hypoxia and interferon gamma response enrichments with the strongest ISG induction (IFIT1, MX1, MX2, and STAT1), a signature compatible with both tumor-intrinsic cytosolic nucleic acid sensing and potential macrophage colocalization (see below). Tumor 2 was dominated by proteostatic stress and partial EMT features across five clones, with HSP90B1 most elevated in T2-D, strong MMP2 and MMP14 expression in EMT-enriched T2-D and T2-E, and complement and vascular signatures concentrated in T2-C. Tumor 3 presented a mesenchymal-invasive landscape, with coagulation (FGA, FGB, FGG, F2, and SERPINC1), extracellular matrix remodeling (SPARC, FN1, COL4A2, and LAMC1), and EMT enrichments peaking in T3-D and T3-E, alongside a proliferative glycolytic clone in T3-B. The depth and specificity of these observations underscore that two regions within the same clinical-subtype tumor can present fundamentally different pathway dependencies, motivating spatially resolved molecular profiling as a complement to existing diagnostic methods (13).

An important methodological consideration in spatial proteomics of solid tumors is the potential contribution of non-tumor cell types (fibroblasts, macrophages, endothelial cells, and lymphocytes) to the observed pathway enrichments. We addressed this directly by quantifying established stromal, myeloid, T-cell, and vascular marker proteins across all clusters in parallel with the pathway analyses (Supplementary Figs. S4, S6 and S8). These analyses revealed that certain clusters (T1-C, T2-C, and T3-E) showed elevated coenrichment of VIM, COL1A1, FN1, ITGAM, ITGB2, LYZ, VWF, and HLA-DRA, indicating dominant stromal, myeloid, and vascular contributions to their molecular signatures. Accordingly, the interferon and complement enrichments in T2-C and the coagulation/complement enrichments in T3-E are interpreted primarily as microenvironmental rather than tumor-cell-intrinsic signals. For T1-D, while canonical interferon-stimulated genes (IFIT1, MX1, MX2, ISG15, and STAT1) were among its most significantly elevated proteins, co-enrichment of macrophage-associated markers (CD14, ITGAM, ITGAX, LYZ, and HLA-DRA) was also detected, raising the possibility of a mixed contribution from tumor-intrinsic nucleic acid sensing and infiltrating myeloid cells. We now explicitly acknowledge this ambiguity in our interpretation, while noting that the hypoxia pathway (the strongest enrichment in T1-D) is not a macrophage-specific program and is consistent with a tumor-cell-intrinsic hypoxic response. Conversely, T1-A, T1-B, and T2-B showed the lowest levels of immune and stromal markers, supporting a tumor-cell-intrinsic basis for their metabolic, redox, and proliferative enrichments. The absence of canonical T-cell (CD3D, CD3E) and macrophage (CD68, CD163) markers from the preprocessed dataset represents a known limitation of MALDI MSI-based proteomics for low-abundance immune proteins and highlights the need for complementary orthogonal methods (such as multiplexed immunofluorescence or spatial transcriptomics) to fully resolve cellular composition in future studies.

Cross-patient coclustering of MALDI MSI proteomic profiles from all three tumors identified three recurring molecular states: a hormone-driven/metabolic profile dominant in tumor 1, a proteostatic-stress/interferon profile most prominent in tumor 2, and a mesenchymal-invasive/coagulation profile dominant in tumor 3. These states partially overlapped between patients (Cohen's Kappa 0.55–0.62; Supplementary Fig. S9*A*), and examination of drug target protein expression across tumors confirmed the cross-patient molecular rationale for the therapeutic choices made: FASN and ACLY (cerulenin targets) were most elevated in tumor 1; proteasome subunits PSMB1, PSMB5, PSMB6, PSMA1, and PSMD1 (bortezomib targets) showed the highest inter-tumor variation; CSNK2A1 (sunitinib target) was highest in tumor 2; and tubulin isoforms TUBB and TUBB4A (paclitaxel targets) were most elevated in tumor 2 (Supplementary Fig. S10). If confirmed in larger cohorts, the existence of a limited number of recurring functional states across luminal breast cancers could form a basis for archetype-informed treatment stratification, analogous to molecular subtyping but at a higher resolution and anchored in spatial protein activity rather than gene expression at the bulk level. However, these observations are based on three cases and must be considered strictly hypothesis-generating at this stage.

### Proteomics-Guided Therapy Selection: Tumor-By-Tumor Evidence

For tumor 1, the proteomics framework predicted resistance to paclitaxel and sensitivity to the cerulenin–sunitinib combination. This prediction was directly validated in the matched PDT. Paclitaxel showed near-complete resistance (IC_50_ = 37,728 nM; R^2^ = 0.40), consistent with high expression of established paclitaxel resistance markers: EDIL3 and CapG (elevated in T1-B and T1-D of the primary tumor), CA12 (highest in T1-D), and PGK1 (broadly expressed across all clones), all confirmed in the tumoroid prior to treatment (Supplementary Fig. S13) (32, 33, 34, 35, 36). Cerulenin and sunitinib as single agents already substantially outperformed paclitaxel, and their combination reduced IC_50_ values approximately 2.7- and 2.8-fold relative to single-agent use, with steeper dose-response curves indicative of improved killing efficiency (Fig. 6*B*). The mechanistic basis is consistent with the proteomic data: FASN (the primary cerulenin target) was most elevated in clone T1-A (Supplementary Fig. S3*B*), and sunitinib's multikinase activity is aligned with the hypoxia and signaling dependencies of T1-D. Importantly, the cerulenin–sunitinib combination showed the strongest tumor-of-origin specificity in cross-validation, with IC_50_ fold-changes exceeding 5-fold in nonmatching tumoroid models (Supplementary Fig. S17), consistent with the lower FASN and ACLY expression in tumors 2 and 3 (Supplementary Fig. S10). Sunitinib was tested at concentrations (approximately 11.5 μM) exceeding reported patient plasma levels (0.15–0.25 μM) (31, 45); this discrepancy should be interpreted cautiously. It is noted that sunitinib remains under clinical investigation in breast cancer and that plasma concentrations do not reflect intratumoral drug exposure over a 7-day *in vitro* treatment period. Clinical translation of this combination would require evaluation of a validated FASN inhibitor such as TVB-2640, for which intratumoral pharmacokinetic data in breast cancer are being accumulated.

For tumor 2, the proteomics-guided combination of bortezomib and dasatinib demonstrated clear superiority over the standard E/C/P regimen, both as single agents and in combination. Bortezomib (IC_50_ = 29.46 nM) and dasatinib (IC_50_ = 37.56 nM) individually outperformed all E/C/P components, with cyclophosphamide showing a poorly fitted dose–response curve (R^2^ = 0.11) indicating an irregular and likely nonspecific response across the tested concentrations. In combination, dasatinib IC_50_ decreased to 13.31 nM and bortezomib to 9.99 nM (approximately 2.8- and 3.0-fold improvements respectively) with consistently steeper dose–response curves (Fig. 7*B*). The molecular rationale is directly supported by the proteomic data: bortezomib was selected based on elevated HSP90B1, ACAT1, and ALDH1A1 in T2-C and T2-D (indicative of proteasomal and unfolded protein response burden) (37, 38, 39), while dasatinib was directed at the PI3K/AKT/mTOR signaling enrichment in T2-D and T2-E (elevated PIK3R1, CASP7) and the kinase-driven features of T2-B (40, 41, 42). We note that the bortezomib–dasatinib combination did not demonstrate formal pharmacological synergy: while the combination IC_50_ values are meaningfully lower than single-agent values, we did not formally calculate combination indices or assess Bliss/Loewe additivity. The observed improvement is therefore best interpreted as additive potency arising from complementary mechanisms rather than true synergy. Cross-validation showed only modest specificity for this combination (∼1.3–2-fold IC_50_ shifts in nonmatching tumoroids; Supplementary Fig. S18), consistent with the broad cellular relevance of proteasome function and kinase signaling across tumor types.

For tumor 3, the paclitaxel–bortezomib combination outperformed the E/C/P regimen, primarily by demonstrating high single-agent potency for both selected drugs (paclitaxel IC_50_ = 15.24 nM; bortezomib IC_50_ = 28.58 nM) and by confirming that cyclophosphamide was largely ineffective (IC_50_ = 13,741 nM; Fig. 8*A*). The proteomics rationale for paclitaxel rested on mitotic spindle enrichment in T3-B and significantly elevated TUBB expression (*p* = 0.045) and TUBA4A (*p* = 0.038) in T3-B and T3-A (Supplementary Fig. S7*B*) (43). Bortezomib was selected to address the secretory and proteasomal burden in mesenchymal clones T3-D and T3-E, supported by significantly elevated LAMB1, LAMC1, SPARC, COL4A2, FN1, and the chaperone CANX. However, in the combination setting, paclitaxel and bortezomib IC_50_ values (8.69 nM and 26.06 nM respectively) remained comparable to those of the respective single agents, indicating the absence of clear additive or synergistic activity beyond what either drug achieved individually, a direct limitation that we explicitly acknowledge. The proteomics-guided approach for tumor 3 therefore demonstrates its value primarily in the identification of effective single agents (notably by replacing cyclophosphamide with bortezomib), rather than in demonstrating a synergistic combination. Cross-validation confirmed high potency of this combination across all three tumoroid models (∼1.3–2-fold IC_50_ shifts; Supplementary Fig. S19), consistent with the fundamental cellular role of microtubule dynamics and proteasome function, and underscoring the limited tumor-of-origin specificity of combinations targeting core cellular processes.

The cross-validation experiments allow a more nuanced assessment of the proteomics framework's predictive accuracy. Across the three combinations, a clear gradient of tumor-of-origin specificity was observed: cerulenin–sunitinib (tumor 1) showed the strongest specificity (>5-fold IC_50_ shifts), bortezomib–dasatinib (tumor 2) showed intermediate specificity (1.3–2-fold), and paclitaxel–bortezomib (tumor 3) showed the weakest (1.3–2-fold). This gradient is mechanistically interpretable: combinations that target tumor-enriched metabolic dependencies (lipid synthesis and receptor kinase signaling in tumor 1) are inherently more tumor-specific than combinations targeting fundamental cellular housekeeping processes (proteasome function and microtubule dynamics) that are broadly active across cancer types. Notably, the bortezomib–paclitaxel combination partially overcame paclitaxel resistance in tumor 1 tumoroids (IC_50_ approximately 6 nM *versus* 37,728 nM for paclitaxel alone), an observation that was not a primary prediction of the framework and that warrants prospective investigation to determine whether proteasome inhibition can reverse tubulin-independent resistance mechanisms *in vivo*. Taken together, the cross-validation data support the proteomics-guided approach most convincingly for tumor 1, provide partial support for tumor 2, and demonstrate principal value in correct agent exclusion rather than true combination synergy for tumor 3.

An important consideration when interpreting IC_50_ values from *in vitro* tumoroid assays is their relationship to clinically achievable drug concentrations. For paclitaxel, reported plasma concentrations in patients range from 80 to 280 nM (31). The IC_50_ values observed in tumors 2 and 3 (13.18 nM and 10.12 nM respectively) are well within this range, supporting clinical relevance of the observed sensitivity. For bortezomib, clinical administration corresponds to 0.1 to 0.3 μM plasma exposure (37, 39), and the IC_50_ values in tumors 2 and 3 (29 nM) are substantially below this threshold. Similarly, for dasatinib, clinical doses correspond to 0.12 to 0.22 μM plasma levels (41, 42), and the IC_50_ of 29.46 nM in tumor 2 falls comfortably within this range. For epirubicin (9.2–16.6 μM clinically) and cyclophosphamide (180–215 μM), the dose–response profiles observed in our experiments remained within clinically accessible ranges. The principal exception is sunitinib, where *in vitro* activity in tumor 1 was observed at concentrations substantially higher than reported plasma levels (0.15–0.25 μM) (30, 31, 44, 45). This caveat is explicitly acknowledged, and direct in vitro-to-in vivo comparisons for this compound should be made with caution, as plasma concentrations do not reflect intratumoral drug levels and clinical pharmacokinetics over prolonged treatment periods differ substantially from a 7-day *in vitro* exposure.

PDTs served as the functional validation platform in this study, and their relevance rests on the degree of molecular concordance with the source tumors. Cosine similarity analysis confirmed that tumoroid proteomic profiles shared substantial concordance with their matched tumor epithelial clusters (similarity scores 0.62–0.66 for tumor 1, 0.59–0.72 for tumor 2, and 0.54–0.70 for tumor 3; Supplementary Fig. S11), while stromal clusters showed substantially lower scores, confirming the tumor cell-enriched nature of the PDT models. Key drug target proteins identified in the primary tumor were confirmed present in the tumoroids prior to treatment (Supplementary Figs. S12–S13; Supplementary Spreadsheets 10 and 11), providing direct molecular support for the PDT as a tractable functional proxy. These findings are consistent with previous studies demonstrating strong proteomic and phenotypic similarity between PDTs and their matched parental tumors (12, 13). At the same time, PDTs do not fully recapitulate the immune microenvironment, stromal interactions, or systemic pharmacokinetics present *in vivo*, and this should be borne in mind when extrapolating drug response data to the clinical context.

### Limitations

Several limitations of this study should be explicitly acknowledged. First and most importantly, the sample size of three tumors is insufficient to draw statistically generalizable conclusions about the predictive value of the pipeline, the prevalence of the three molecular archetypes identified, or the clinical benefit of the proposed drug combinations. A larger, prospectively collected cohort will be required to determine whether the three recurring functional states observed here are stable and reproducible across luminal breast cancers and whether they can predict treatment response. Second, while the proteomics-guided combinations outperformed standard-of-care regimens in the tumoroid models, formal pharmacological synergy was not established for any combination. Combination IC_50_ reductions were observed for tumors 1 and 2, but no combination index analysis, Bliss independence model, or Loewe additivity calculation was performed; conclusions about synergy therefore exceed what the data support. Third, for tumor 3, the paclitaxel–bortezomib combination did not improve upon the potency of either single agent, which limits the strength of the proteomics-guided strategy for this tumor and underscores that the framework is not universally additive. Fourth, the absence of canonical immune cell markers (CD3D, CD3E, CD68, and CD163) from the preprocessed proteomics dataset is a known technical limitation of MALDI MSI-based DIA proteomics for low-abundance proteins and prevents a fully resolved assessment of cellular composition in all clusters. Fifth, several proposed compounds (cerulenin–sunitinib, bortezomib–dasatinib) have not been evaluated in clinical trials for luminal breast cancer, and their tolerability and systemic pharmacokinetics as combinations are unknown. Sixth, cerulenin is a research-grade natural product; clinical translation would require substitution with a clinically validated FASN inhibitor such as TVB-2640. Finally, as with all *ex vivo* systems, PDTs do not capture the full complexity of the tumor microenvironment, systemic immune responses, or the pharmacokinetic constraints that govern drug exposure in patients.

### Future Directions

Future work should prioritize several directions. First, prospective validation in a larger cohort, ideally with paired tissue proteomics and PDT drug testing, is essential to determine whether the pipeline improves clinical decision-making. Second, integration of complementary spatial modalities such as multiplexed immunofluorescence, CyCIF, or spatial transcriptomics would allow cellular composition to be resolved at single-cell resolution, addressing the key limitation of myeloid and stromal marker ambiguity identified in this work. Third, formal synergy quantification using established models (Bliss, Loewe, or ZIP) should be incorporated into future combination experiments, both to strengthen mechanistic conclusions and to identify dose windows where true synergistic activity exists. Fourth, comparison of the three molecular archetypes described here with publicly available proteogenomic datasets such as CPTAC breast cancer cohorts would allow their prevalence, stability, and clinical associations to be assessed at scale. Finally, whether this approach can be implemented within a clinically actionable timeframe, from biopsy to PDT-validated drug selection, and whether it translates into improved patient outcomes remains to be demonstrated in prospective trials.

## Conclusions

This study demonstrates the feasibility of using spatial proteomics and PDTs in an integrated pipeline to characterize intratumoral heterogeneity and inform drug selection in luminal breast cancer. MALDI MSI analysis revealed molecularly distinct clones within each of the three tumors examined, with pathway dependencies that differed substantially from those predicted by clinical subtype alone. Quantitative assessment of cell-type marker proteins confirmed that several enrichment signals, particularly in clusters with high myeloid or stromal marker co-expression, reflect microenvironmental rather than tumor cell-intrinsic biology, and therapeutic interpretations were adjusted accordingly.

Proteomics-guided drug combinations outperformed standard-of-care regimens in matched tumoroids in two of the three cases (tumors 1 and 3), primarily by identifying effective agents (notably cerulenin, sunitinib, and bortezomib) and excluding demonstrably ineffective ones such as paclitaxel in tumor 1 and cyclophosphamide in tumors 2 and 3. For tumor 2, individual agents outperformed standard-of-care components, but clear combination synergy was not demonstrated. Cross-validation experiments confirmed tumor-of-origin specificity most clearly for the cerulenin + sunitinib combination; for the other combinations, specificity was more limited, and conclusions about predictive accuracy should be drawn with caution.

Cross-tumor analysis identified three recurring molecular profiles (a hormone-metabolic, a proteostatic-stress, and a mesenchymal-invasive archetype) which partially overlapped between patients and were associated with distinct drug sensitivities. These observations are hypothesis-generating and require confirmation in larger cohorts before they can inform clinical stratification.

The principal contribution of this work is methodological: it provides a proof-of-concept for a pipeline that integrates MALDI MSI spatial proteomics, cellular composition analysis, and PDT testing to move from molecular characterization to functionally validated drug selection. The limitations identified: small sample size, partial combination synergy, limited *in vivo* validation, and technical constraints on immune marker detection, define the priorities for future investigation. Prospective trials will ultimately be required to determine whether this approach improves patient outcomes.

## Data availability

The data from this study, including MS DIA raw files, DIA-NN files, and annotated MS/MS datasets, have been deposited to the ProteomeXchange Consortium *via* the PRIDE partner repository with the dataset identifier PXD069792 (Username: reviewer_pxd069792@ebi.ac.uk - Password: OH6dz6MeUqAA).

The MALDI MSI datasets are archived in Zenodo and accessible here: https://doi.org/10.5281/zenodo.19114048

## Supplemental data

Supplementary information’s represents the 15 supplementary figures and 13 spreadsheets.

## Conflicts of Interest

The authors declare no competing interests.

## Supplemental Data

Supplemental Spreadsheet 1

Supplemental Spreadsheet 2

Supplemental Spreadsheet 3

Supplemental Spreadsheet 4

Supplemental Spreadsheet 5

Supplemental Spreadsheet 6

Supplemental Spreadsheet 7

Supplemental Spreadsheet 8

Supplemental Spreadsheet 9

Supplemental Spreadsheet 10

Supplemental Spreadsheet 11

Supplemental Spreadsheet 12

Supplementary MSI Data

Supplementary Figures Legends

Supplementary Spreadsheet Legends
