# Supplementary material for: Clone-By-Clone Therapy Guidance in Luminal Breast Cancer Via Spatial Proteomics and Patient-Derived Tumoroid
Source: Mol Cell Proteomics. 2026 Aug 12;25(9):101639. doi: 10.1016/j.mcpro.2026.101639 (PMC13579033; doi:10.1016/j.mcpro.2026.101639)
Supplement: Supplementary file 1 [file mmc14.pdf]

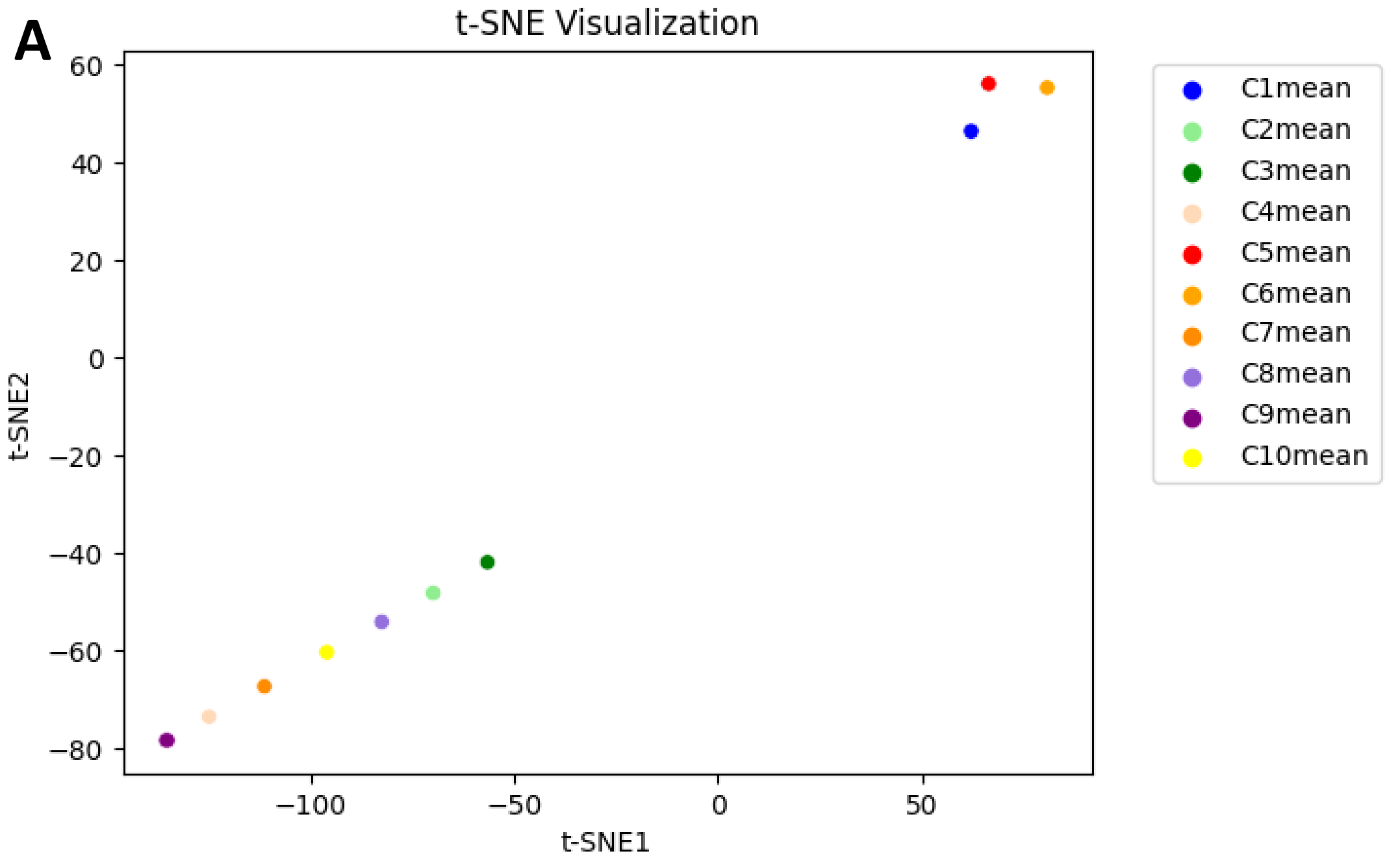


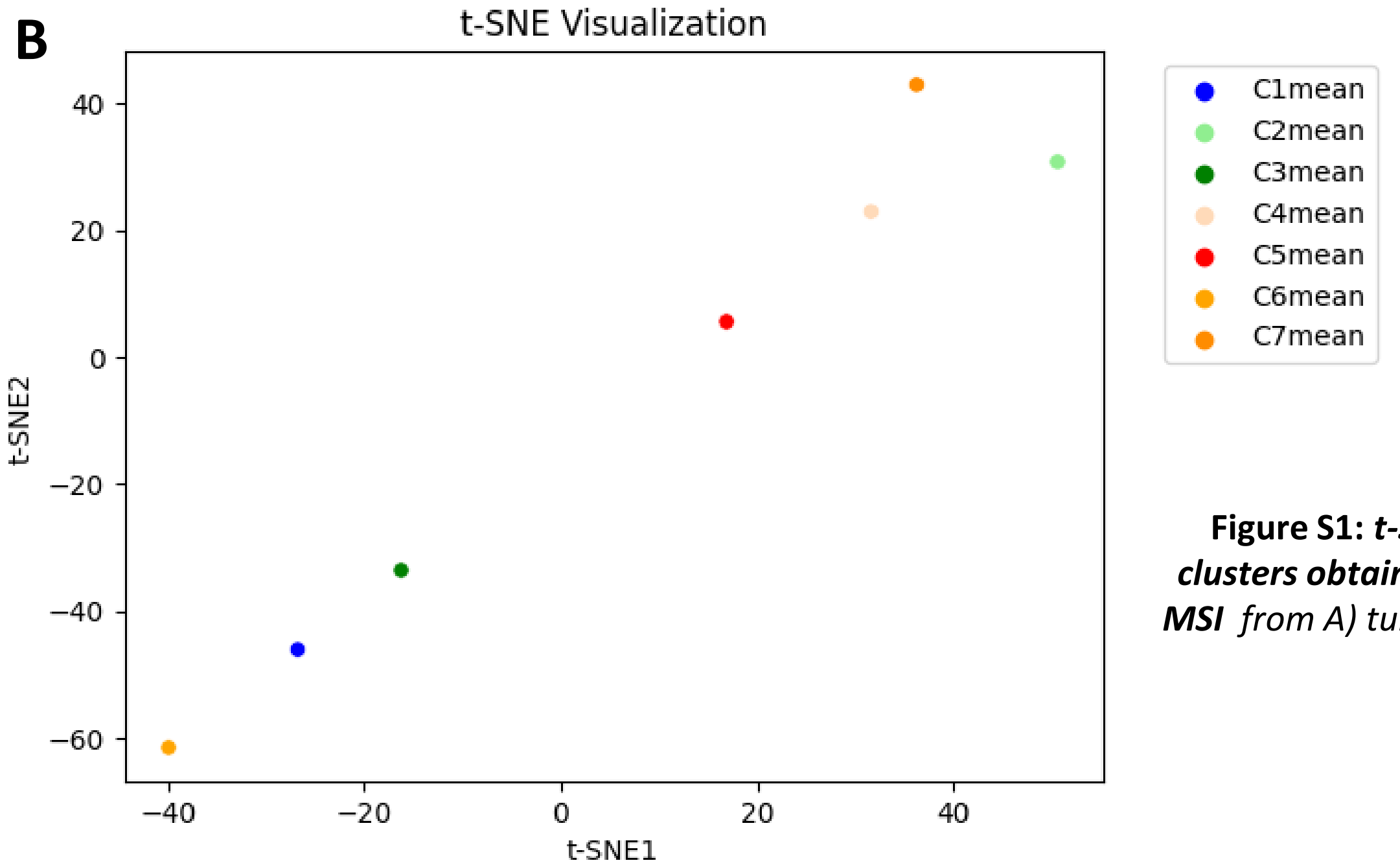


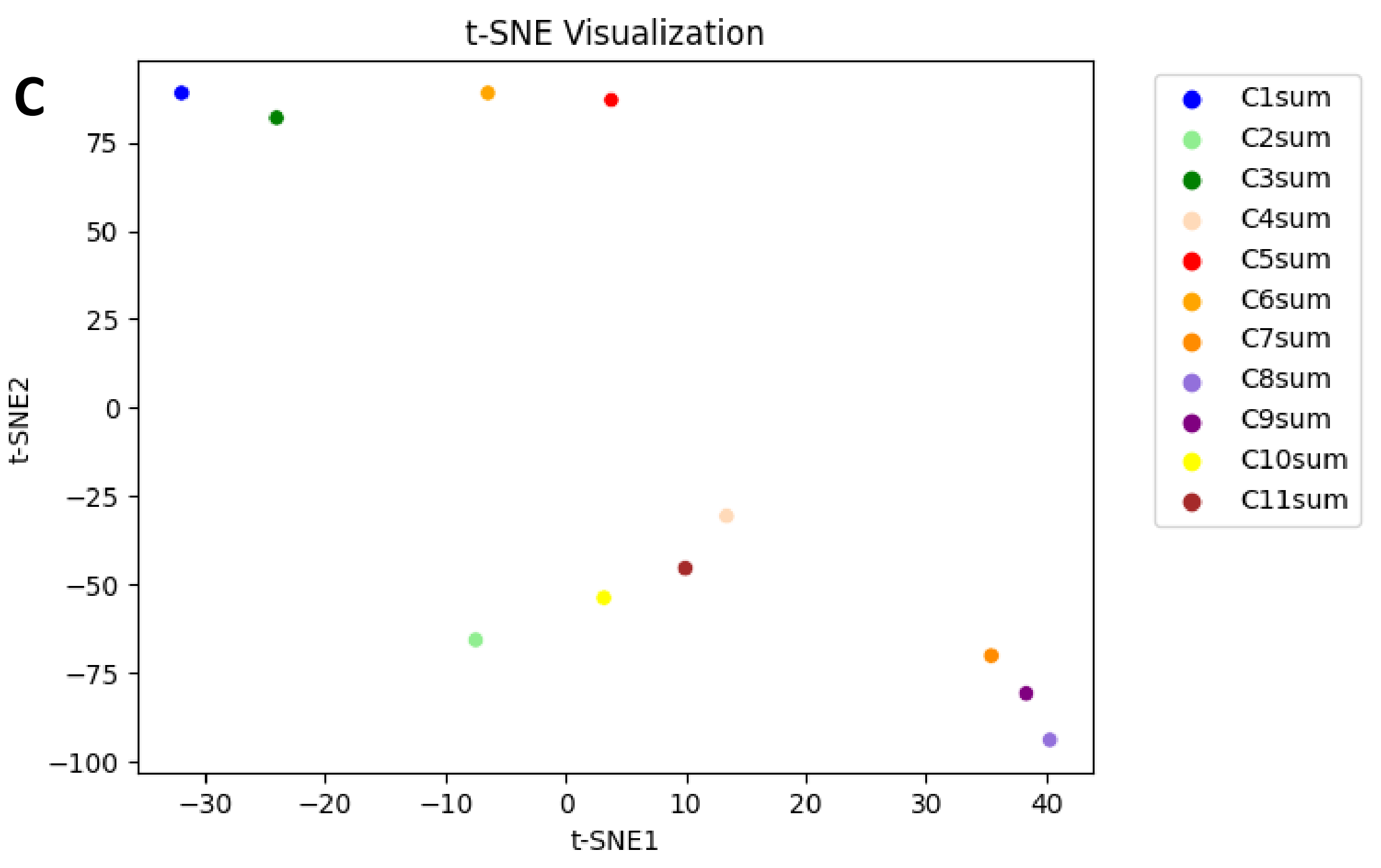


**Figure S1:** ***t-SNE visualization of the clusters obtained after segmentation of MSI*** *from A) tumour 1, B) tumour 2 and C) tumour 3.*

A

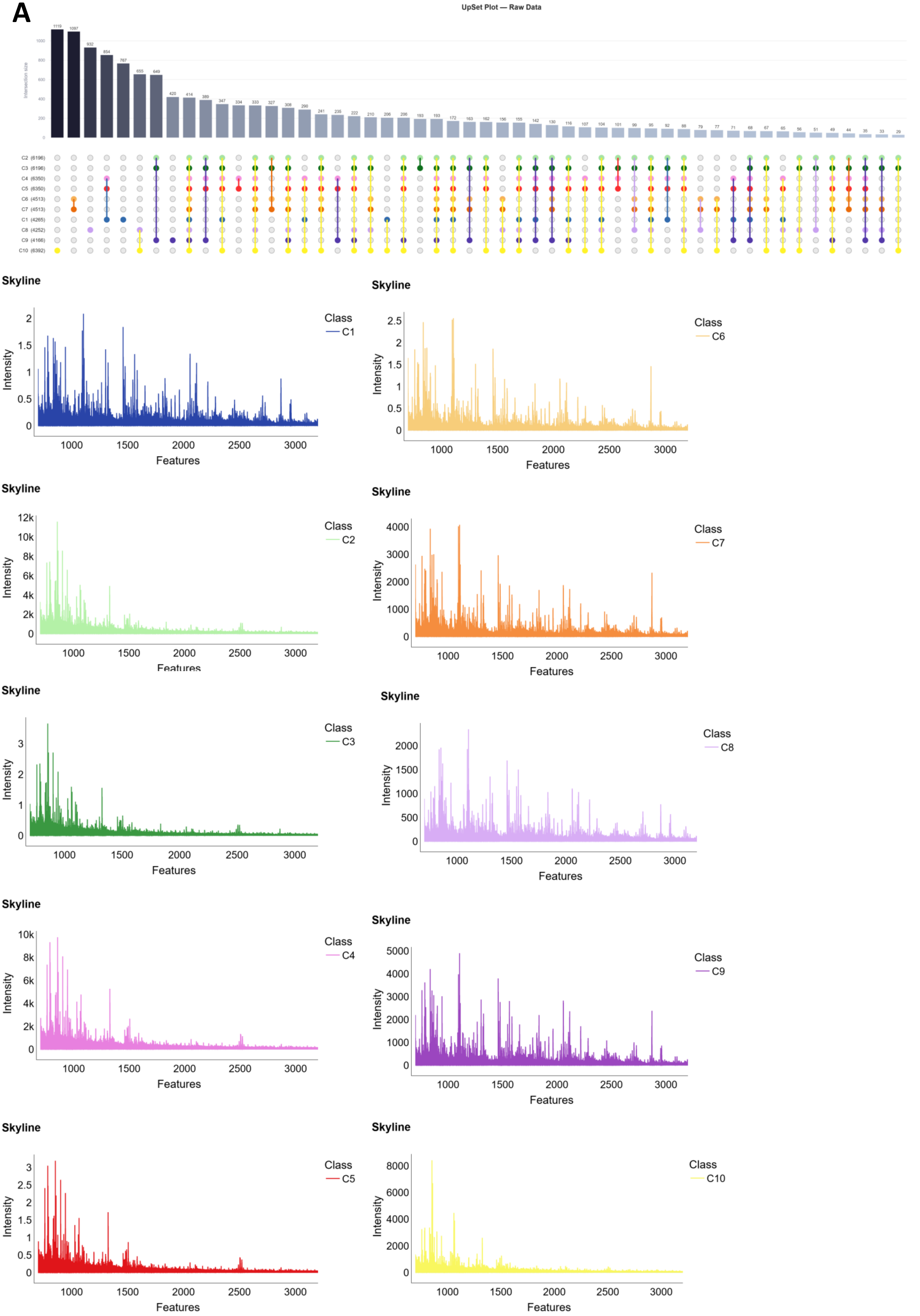
UpSet Plot — Raw Data
Intersection size
C2 (6196)
C3 (6196)
C4 (6350)
C5 (6350)
C6 (4513)
C7 (4513)
C1 (4265)
C8 (4252)
C9 (4166)
C10 (6392)
Skyline
Intensity
Features
Class
C1
C6
C2
C7
C3
C8
C4
C9
C5
C10

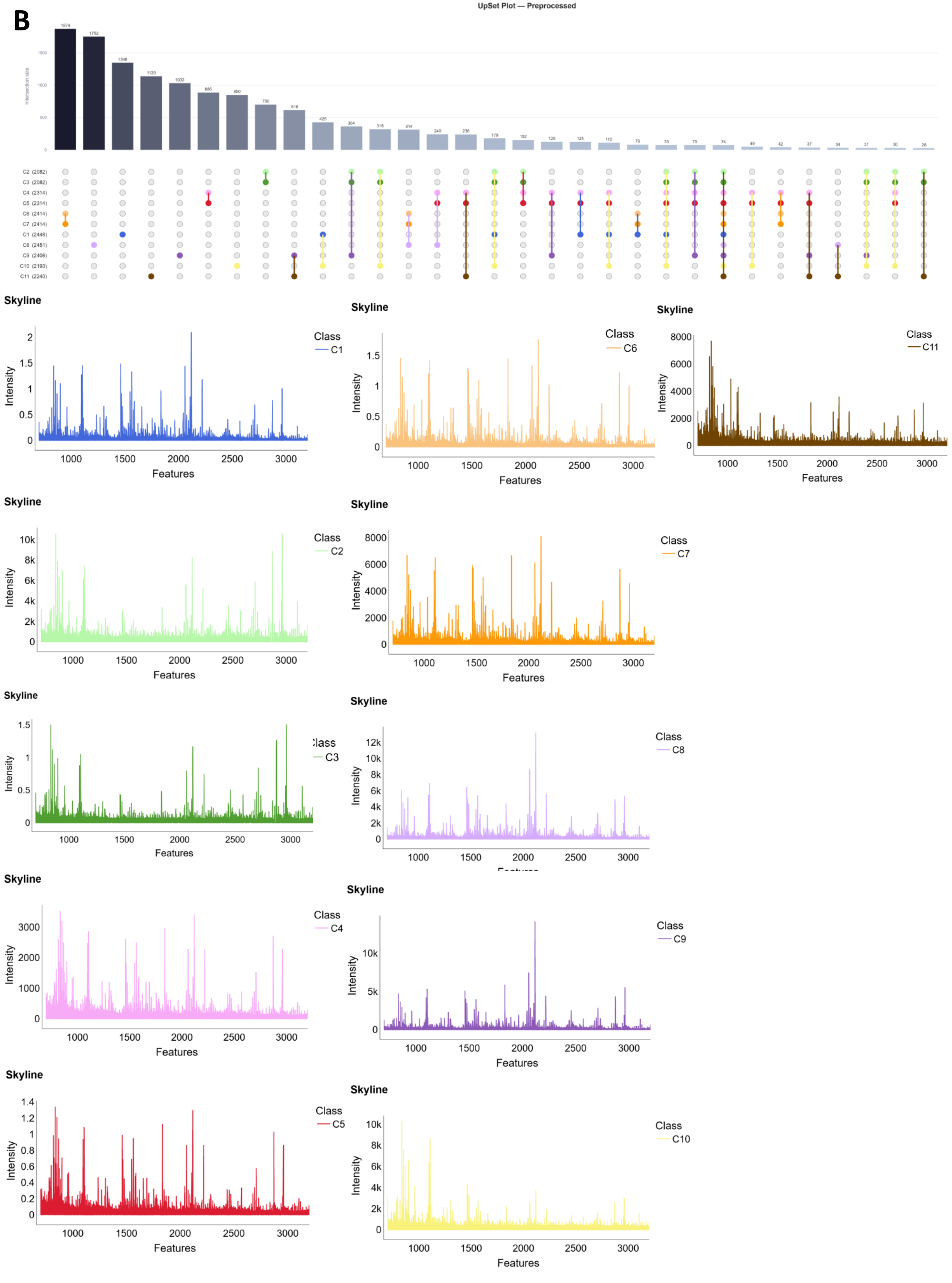
B
UpSet Plot — Preprocessed
Intersection size
1874
1752
1348
1139
1033
886
850
700
616
425
364
318
314
240
238
179
152
125
124
110
79
75
75
74
49
42
37
34
31
30
26
C2 (2082)
C3 (2082)
C4 (2314)
C5 (2314)
C6 (2414)
C7 (2414)
C1 (2448)
C8 (2451)
C9 (2408)
C10 (2193)
C11 (2240)
Skyline
Class
C1
C6
C11
C2
C7
C3
C8
C4
C9
C5
C10
Intensity
Features

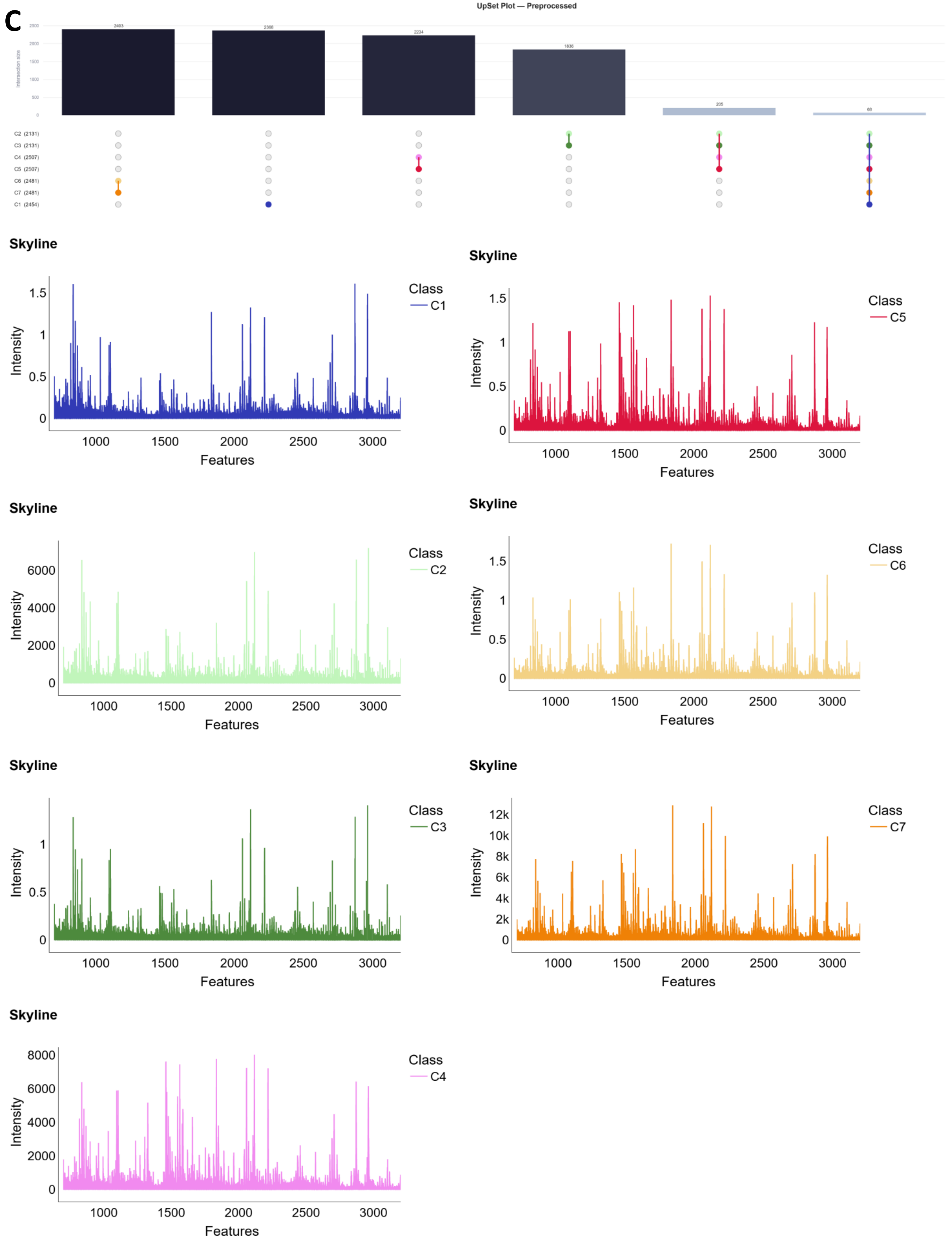


**Figure S2**: ***m/z comparision spectra between each tumour MSI cluster comprising upset plot and cluster mean spectra for A) Tumour 1, B) Tumour 3 and C) Tumour 2.*** *Upset plot supplementary data disponible as Supplementary MSI data.*

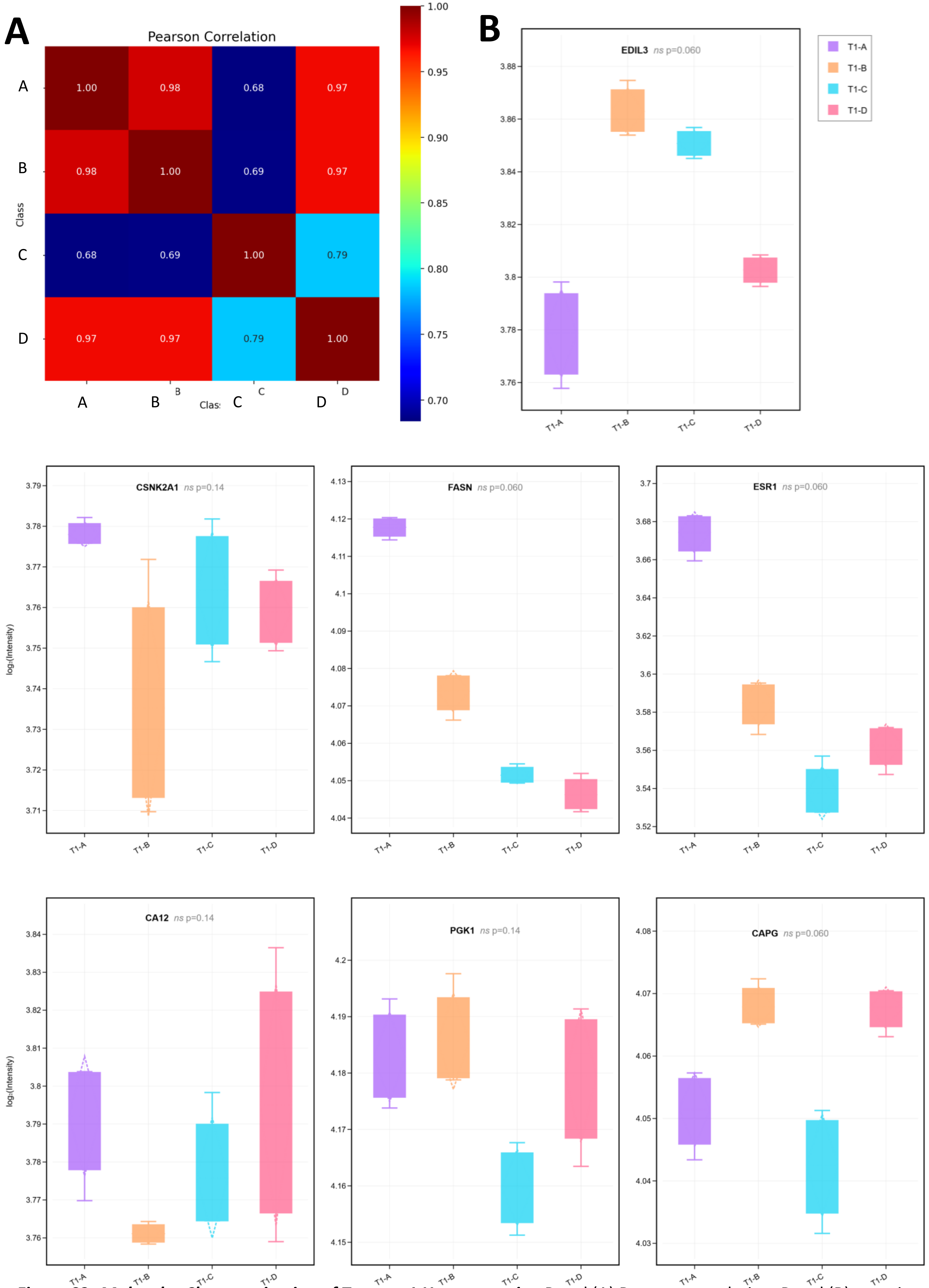


**Figure S3:** ***Molecular Characterization of Tumour 1 Heterogeneity.*** Panel (A) Pearson correlation. Panel (B) protein target enrichment across clones.

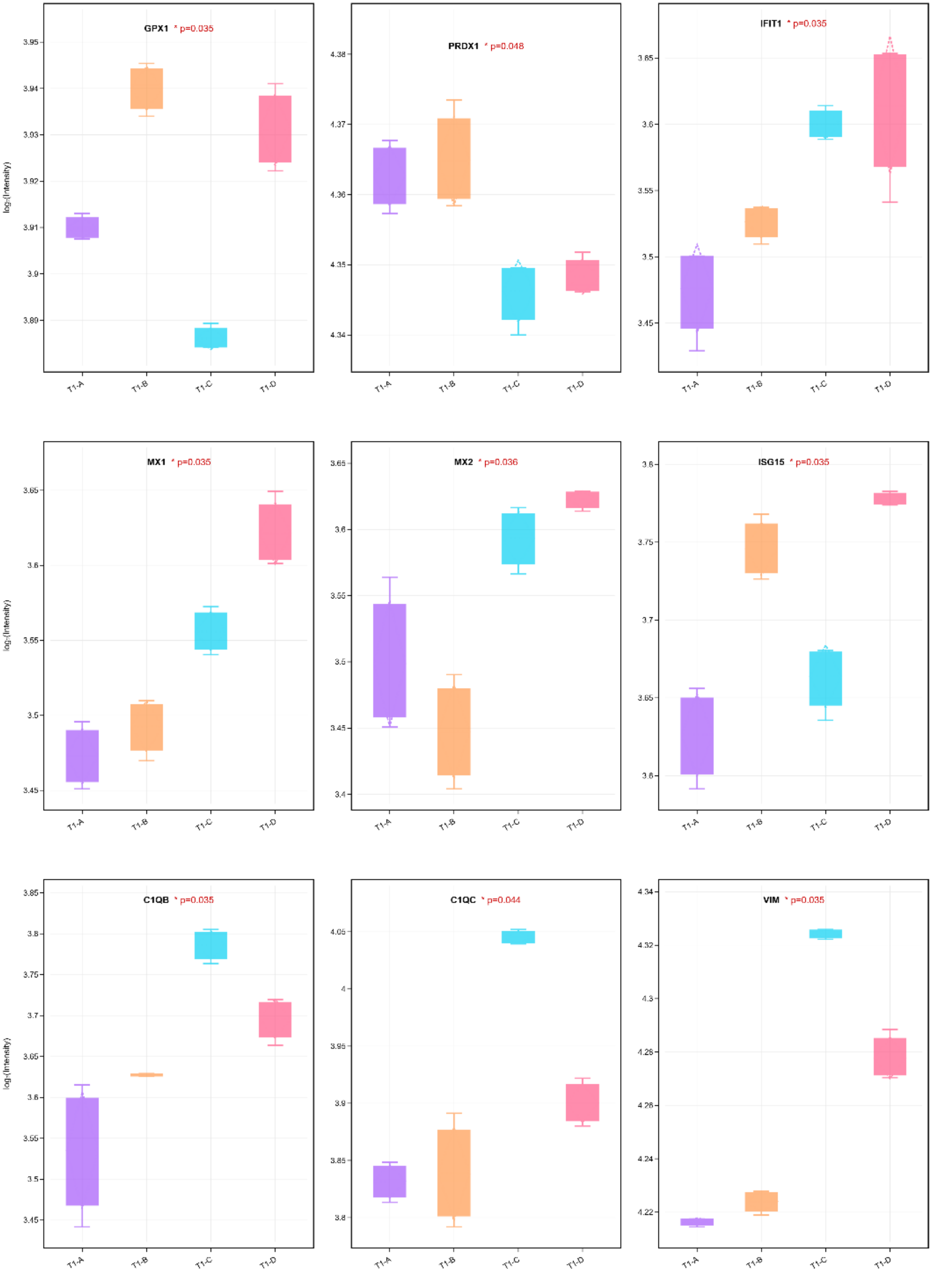
GPX1 * p=0.035
PRDX1 * p=0.048
IFIT1 * p=0.035
MX1 * p=0.035
MX2 * p=0.036
ISG15 * p=0.035
C1QB * p=0.035
C1QC * p=0.044
VIM * p=0.035
log2(Intensity)
T1-A
T1-B
T1-C
T1-D

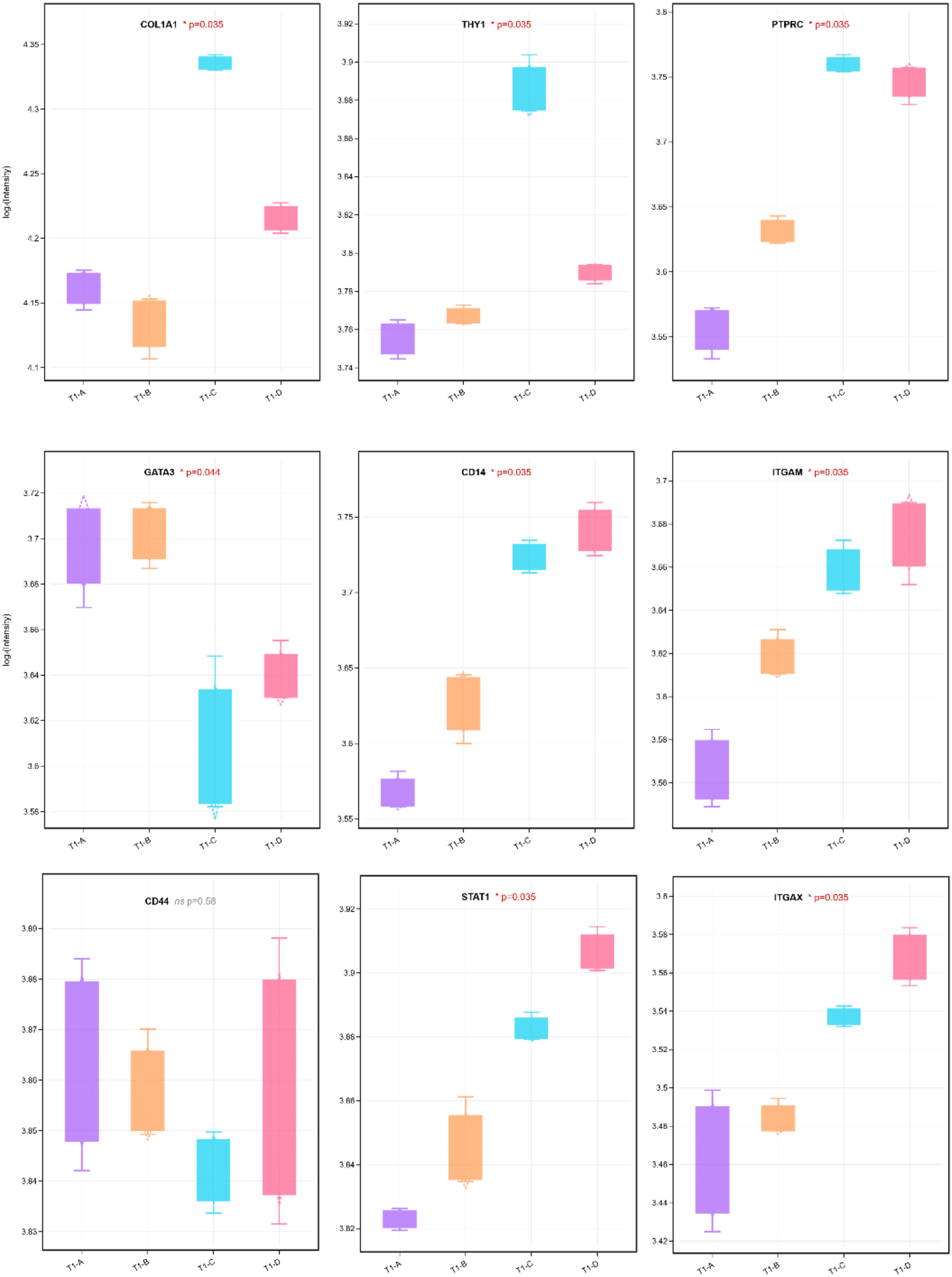

COL1A1 * p=0.035
THY1 * p=0.035
PTPRC * p=0.035
GATA3 * p=0.044
CD14 * p=0.035
ITGAM * p=0.035
CD44 ns p=0.58
STAT1 * p=0.035
ITGAX * p=0.035
log2(Intensity)
T1-A
T1-B
T1-C
T1-D

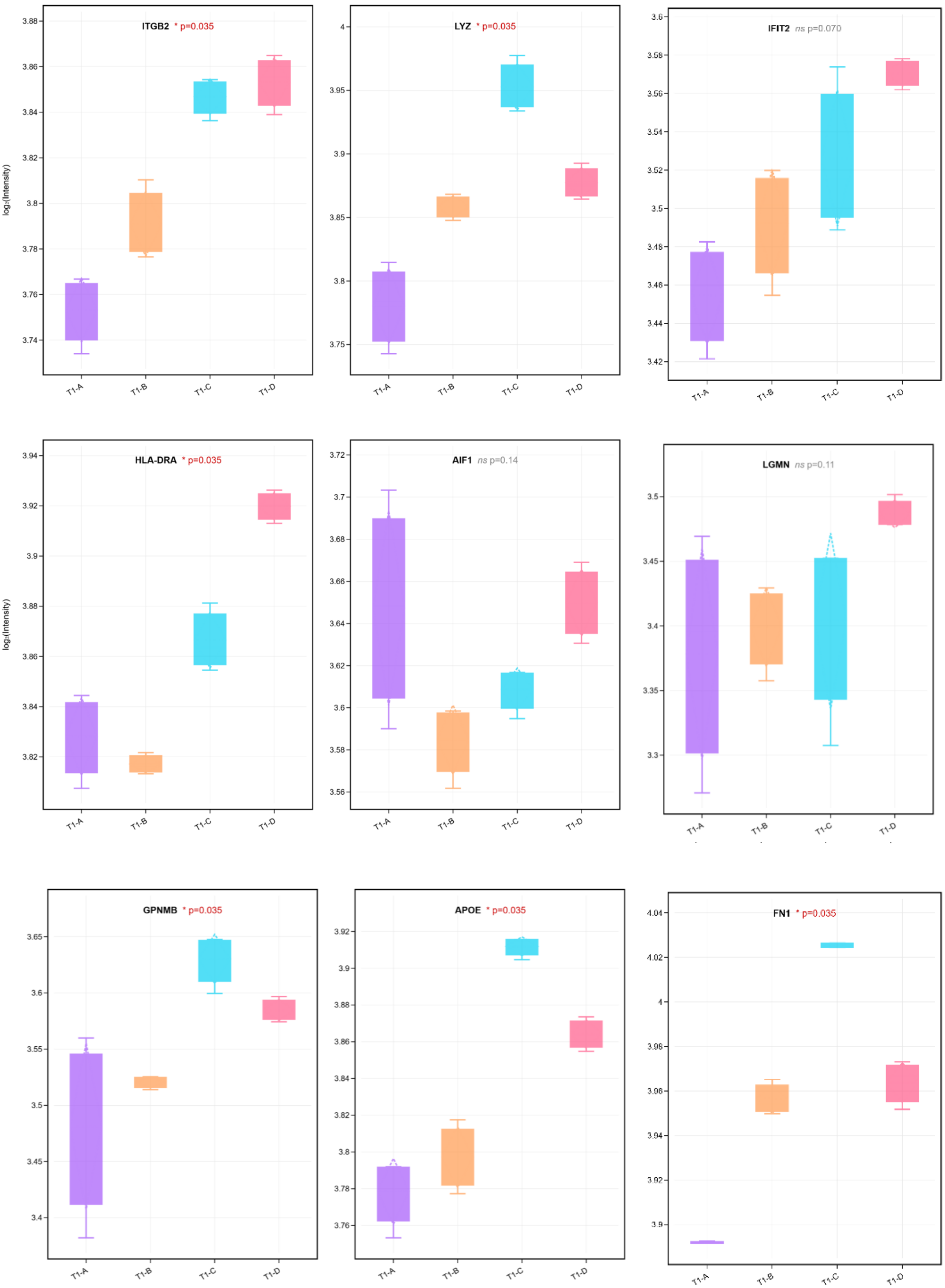


**Figure S4:** ***Stromal, immune, and vascular marker proteins across all Tumour 1 clusters.*** Protein of interest involvement across protein pathways

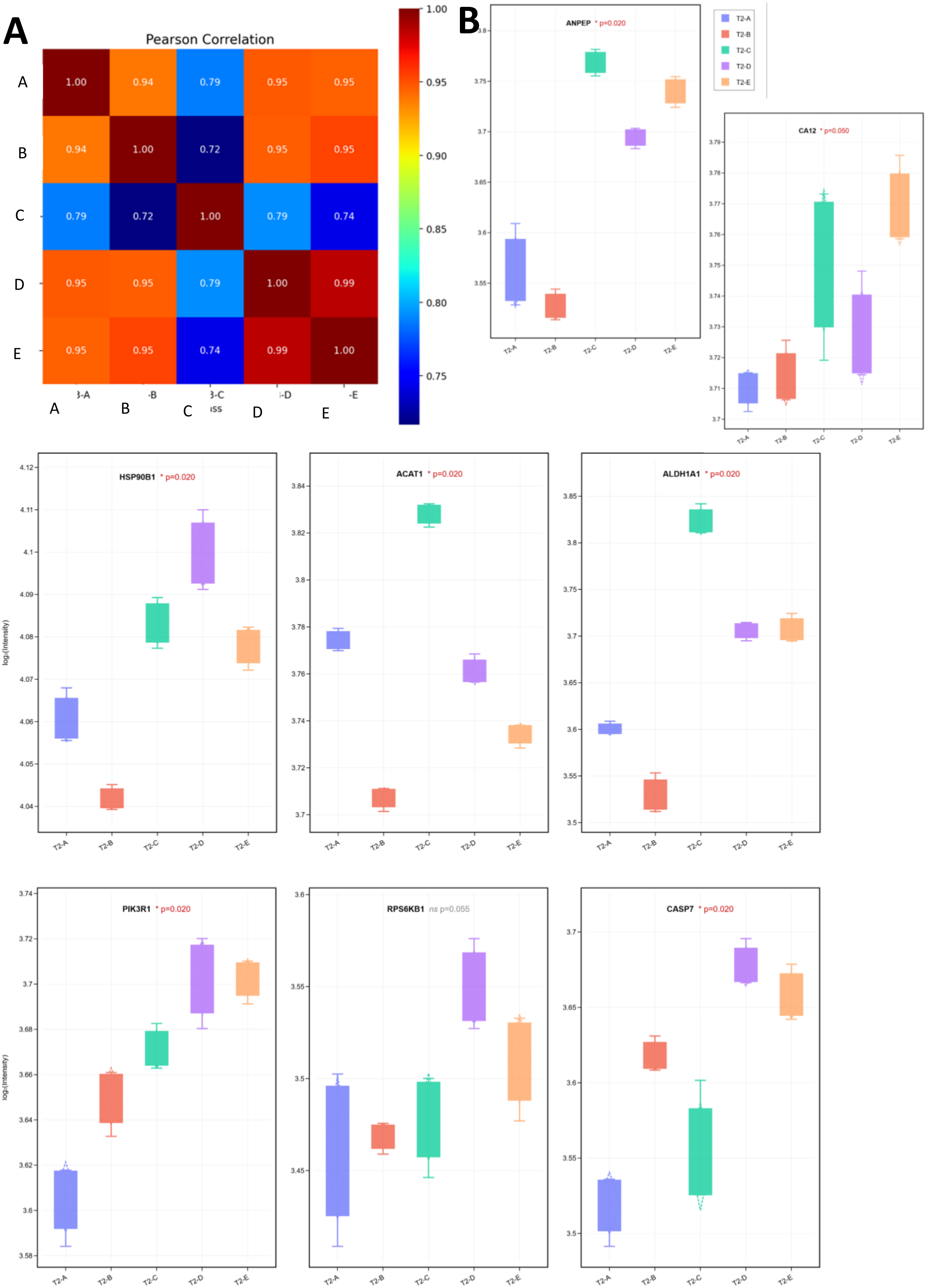


**Figure S5:** ***Molecular Characterization of Tumour 2 Heterogeneity.*** Panel (A) Pearson correlation. Panel (B) protein target enrichment across clones.

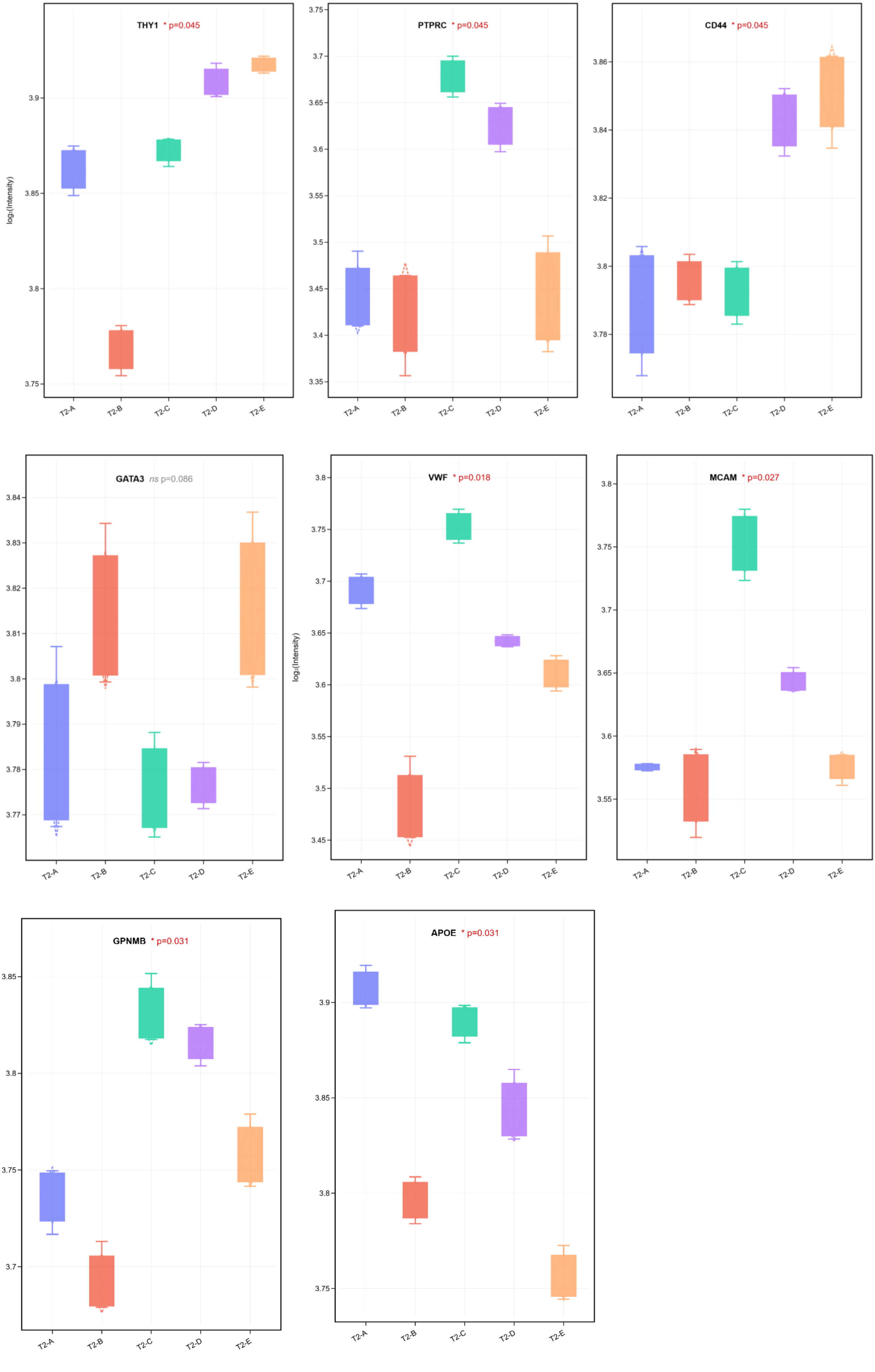
THY1 * p=0.045
log₂(Intensity)
3.75
3.8
3.85
3.9
T2-A
T2-B
T2-C
T2-D
T2-E
PTPRC * p=0.045
3.35
3.4
3.45
3.5
3.55
3.6
3.65
3.7
3.75
T2-A
T2-B
T2-C
T2-D
T2-E
CD44 * p=0.045
3.78
3.8
3.82
3.84
3.86
T2-A
T2-B
T2-C
T2-D
T2-E
GATA3 ns p=0.086
3.77
3.78
3.79
3.8
3.81
3.82
3.83
3.84
T2-A
T2-B
T2-C
T2-D
T2-E
VWF * p=0.018
log₂(Intensity)
3.45
3.5
3.55
3.6
3.65
3.7
3.75
3.8
T2-A
T2-B
T2-C
T2-D
T2-E
MCAM * p=0.027
3.55
3.6
3.65
3.7
3.75
3.8
T2-A
T2-B
T2-C
T2-D
T2-E
GPNMB * p=0.031
3.7
3.75
3.8
3.85
T2-A
T2-B
T2-C
T2-D
T2-E
APOE * p=0.031
3.75
3.8
3.85
3.9
T2-A
T2-B
T2-C
T2-D
T2-E

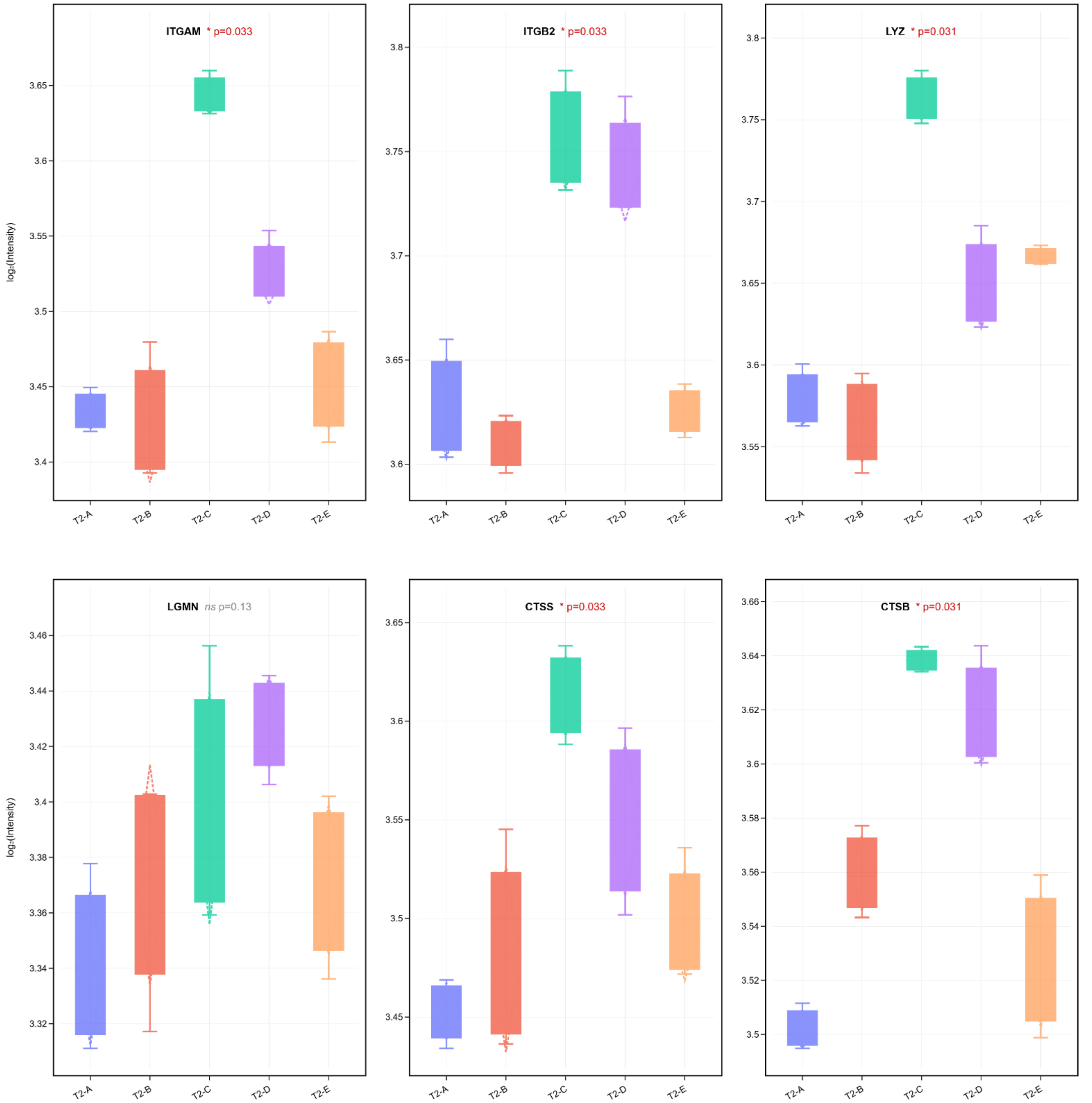
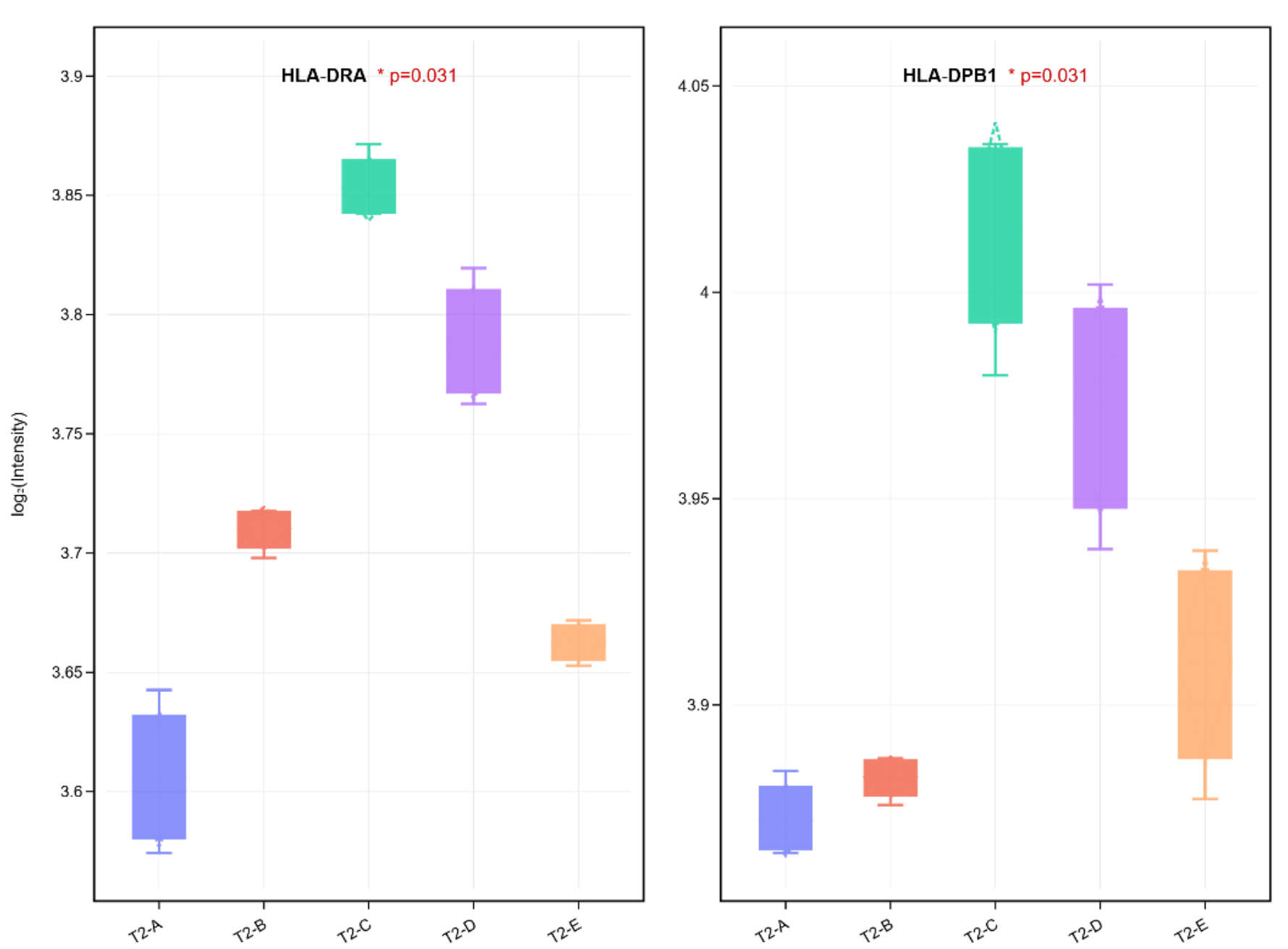


**Figure S6:** ***Stromal, immune, and vascular marker proteins across all Tumour 2 clusters.*** Protein of interest involvement across protein pathways

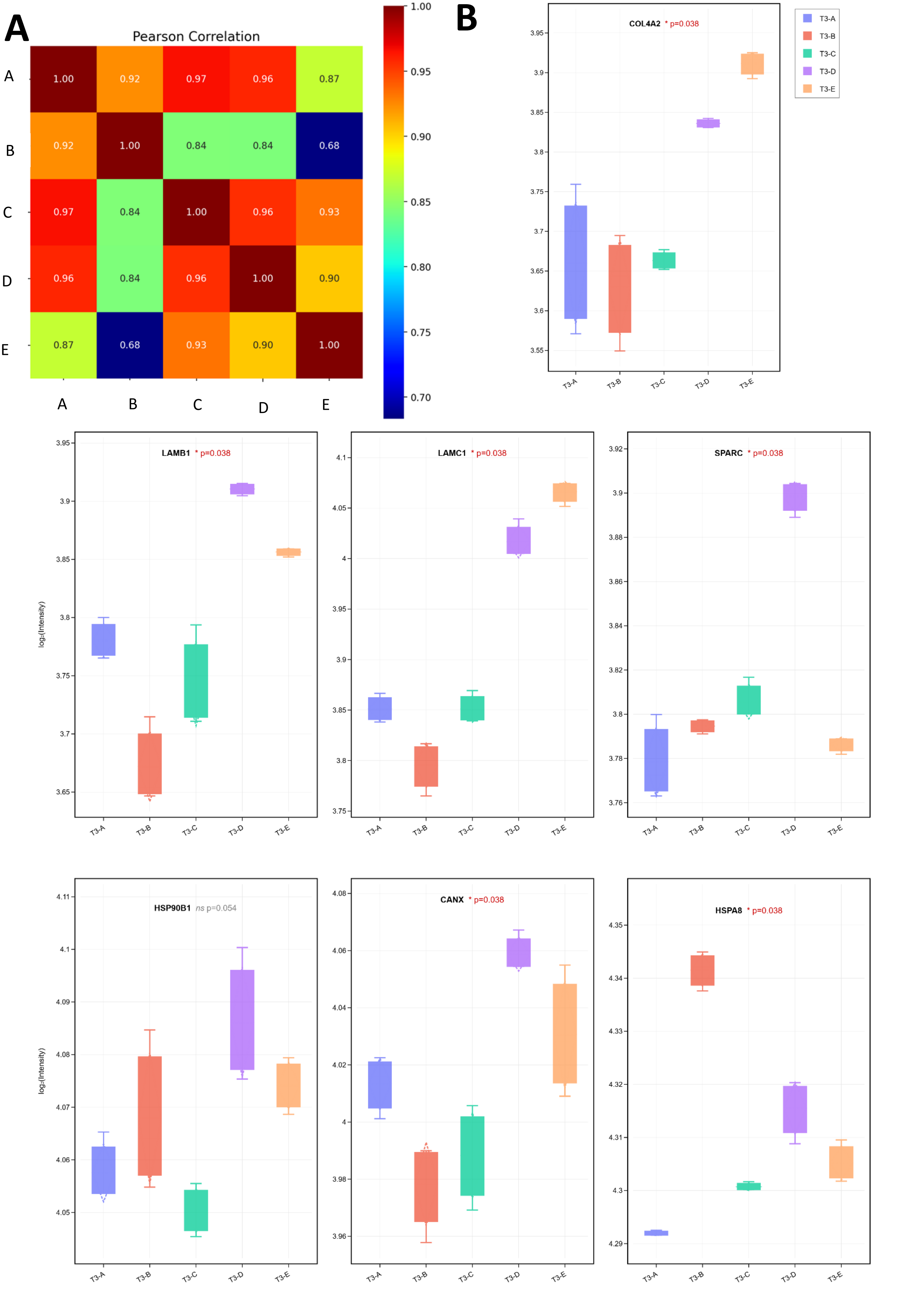
A
Pearson Correlation
A B C D E
1.00 0.92 0.97 0.96 0.87
0.92 1.00 0.84 0.84 0.68
0.97 0.84 1.00 0.96 0.93
0.96 0.84 0.96 1.00 0.90
0.87 0.68 0.93 0.90 1.00
1.00 0.95 0.90 0.85 0.80 0.75 0.70
B
COL4A2 * p=0.038
T3-A T3-B T3-C T3-D T3-E
LAMB1 * p=0.038
LAMC1 * p=0.038
SPARC * p=0.038
HSP90B1 ns p=0.054
CANX * p=0.038
HSPA8 * p=0.038
log₂(Intensity)

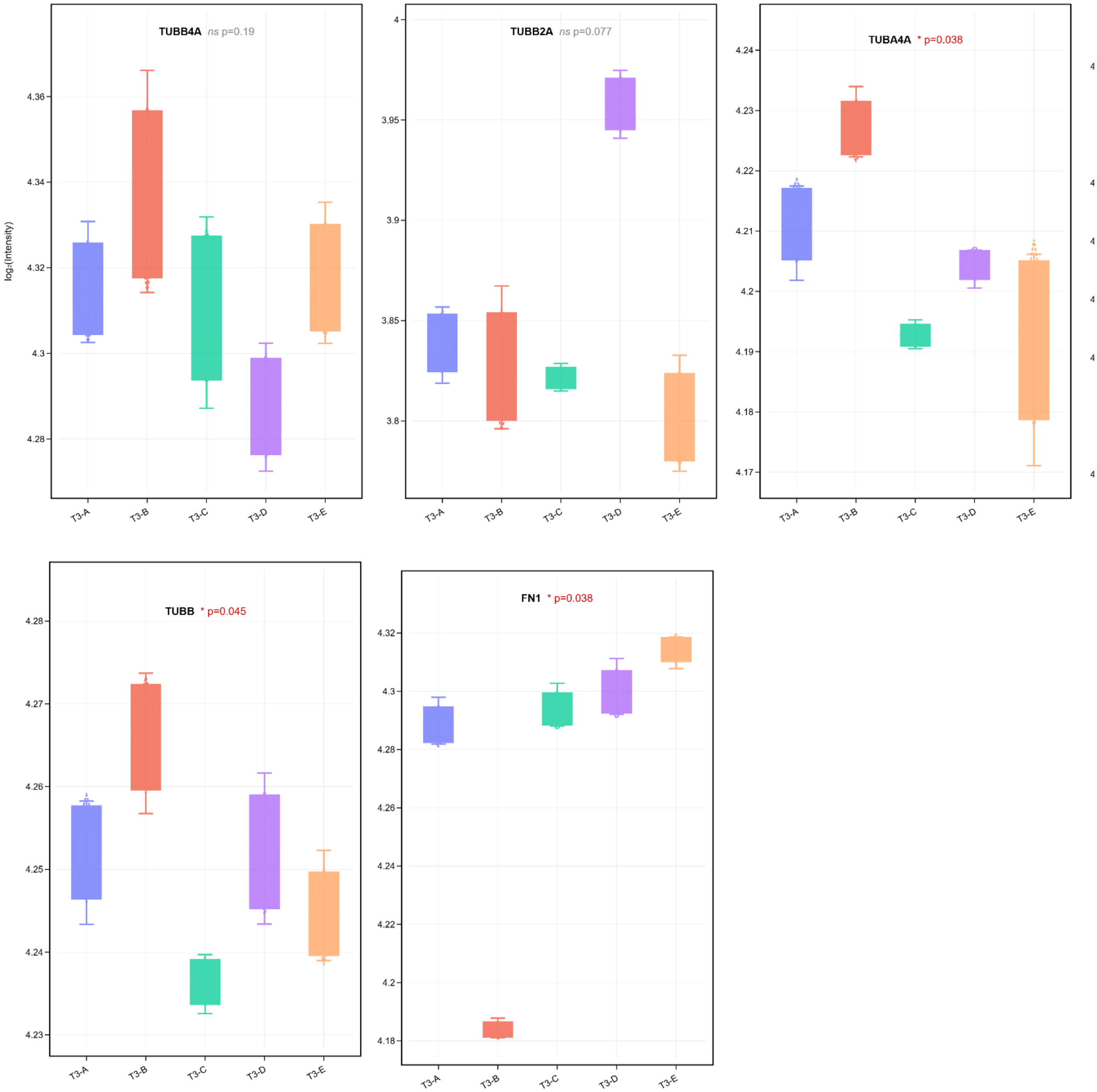


**Figure S7:** ***Molecular Characterization of Tumour 3 Heterogeneity.*** Panel (A) Pearson correlation. Panel (B) protein target enrichment across clones.

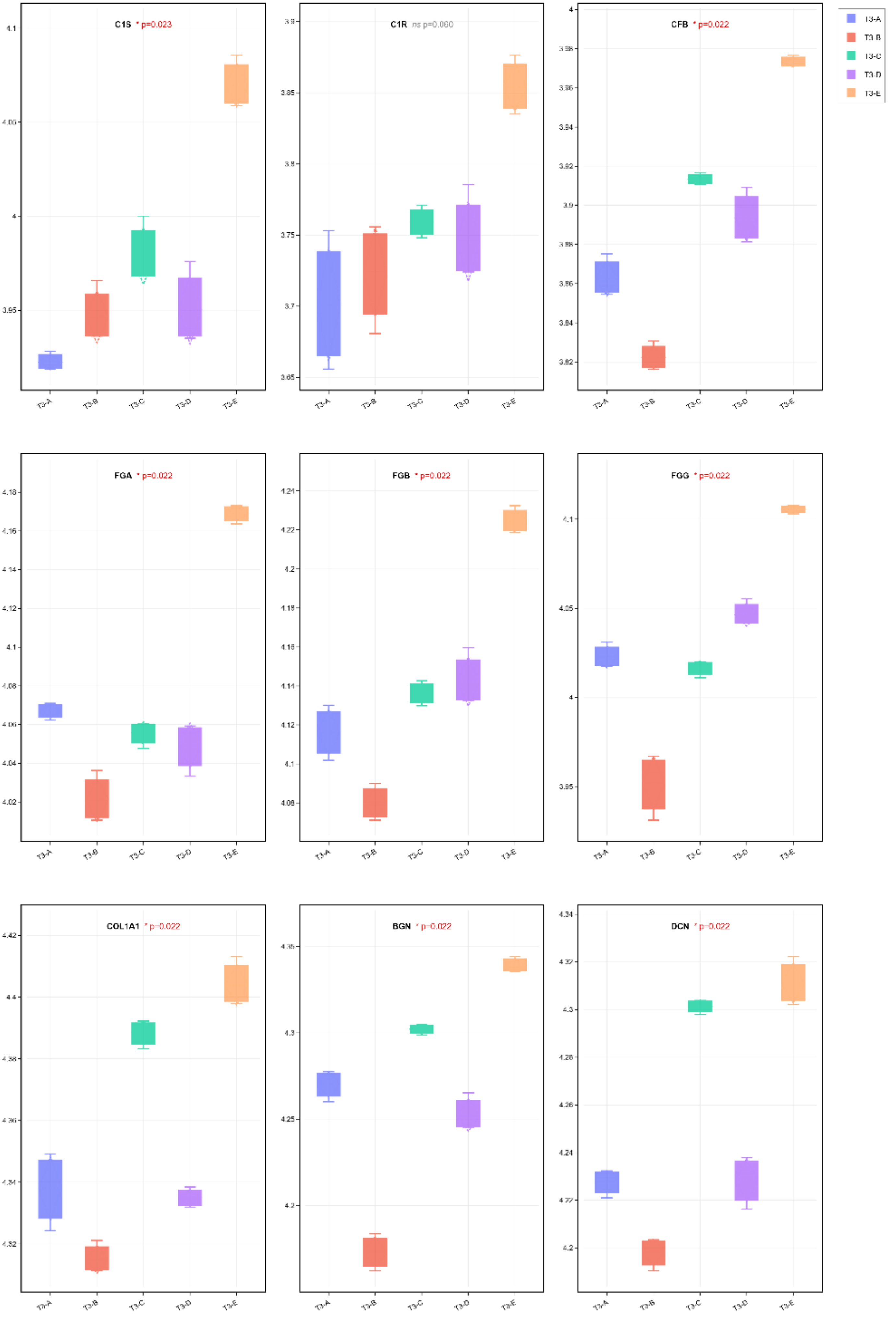

C1S * p=0.023
C1R ns p=0.060
CFB * p=0.022
FGA * p=0.022
FGB * p=0.022
FGG * p=0.022
COL1A1 * p=0.022
BGN * p=0.022
DCN * p=0.022
T3-A
T3-B
T3-C
T3-D
T3-E

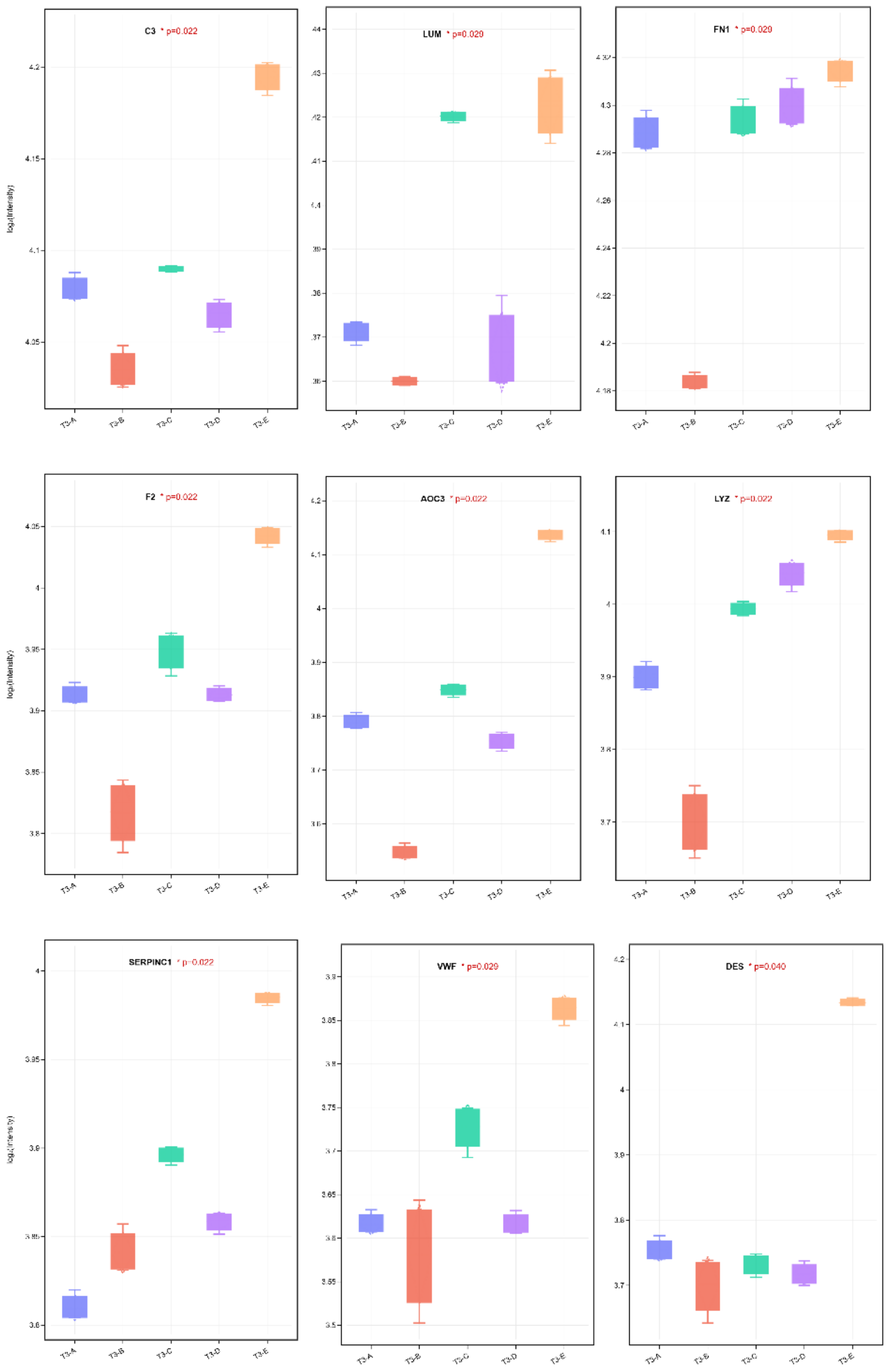


**Figure S8:** ***Stromal, immune, and vascular marker proteins across all Tumour 3 clusters.*** Protein of interest involvement across protein pathways

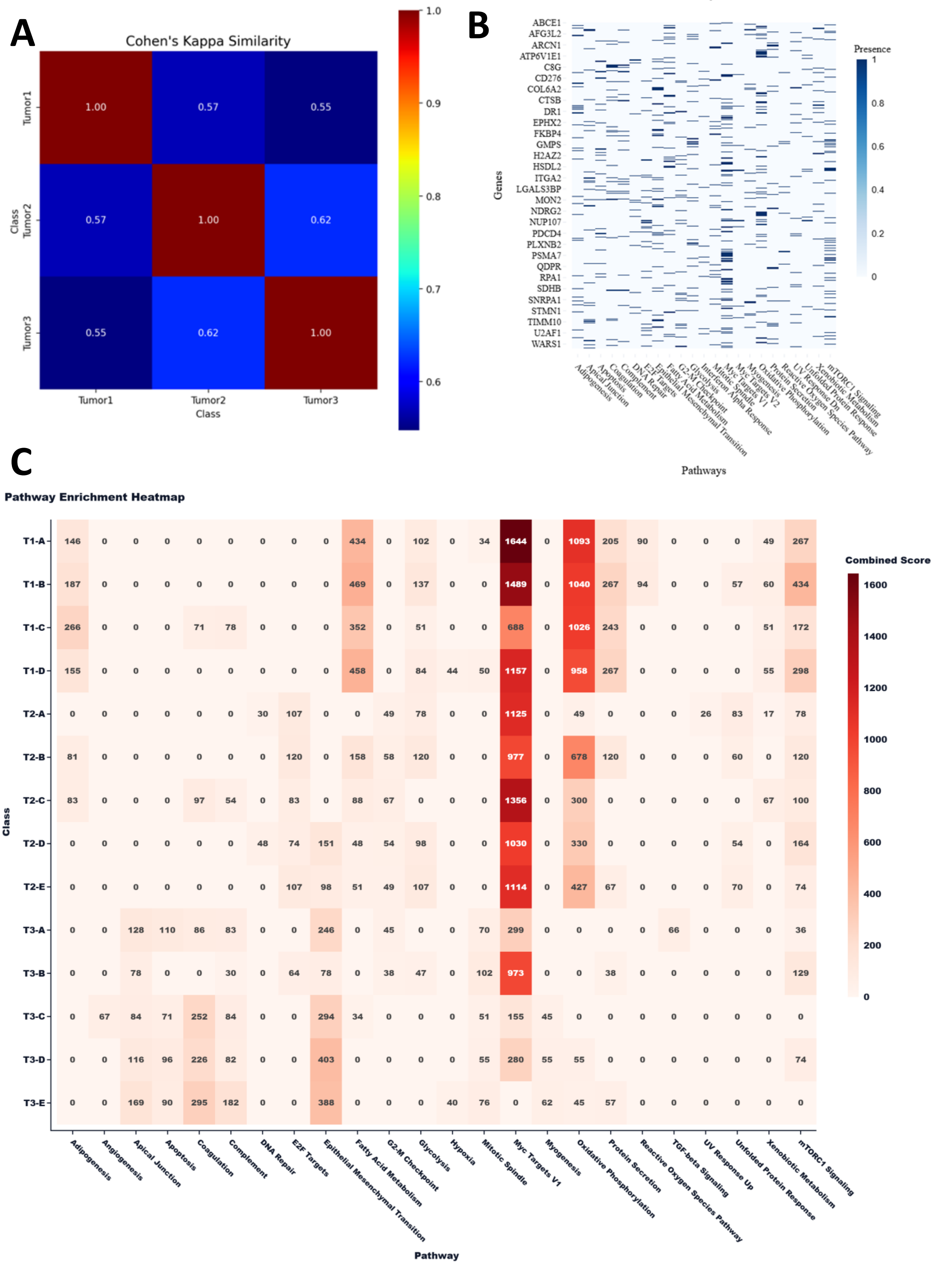


**Figure S9:** ***Spatial and Molecular Characterization of Inter-tumor Heterogeneity.*** Panel (A) Pearson correlation. Panel (B) Gene count per enriched pathway. Panel (C) pathway enrichment across regions selected from all tumors.

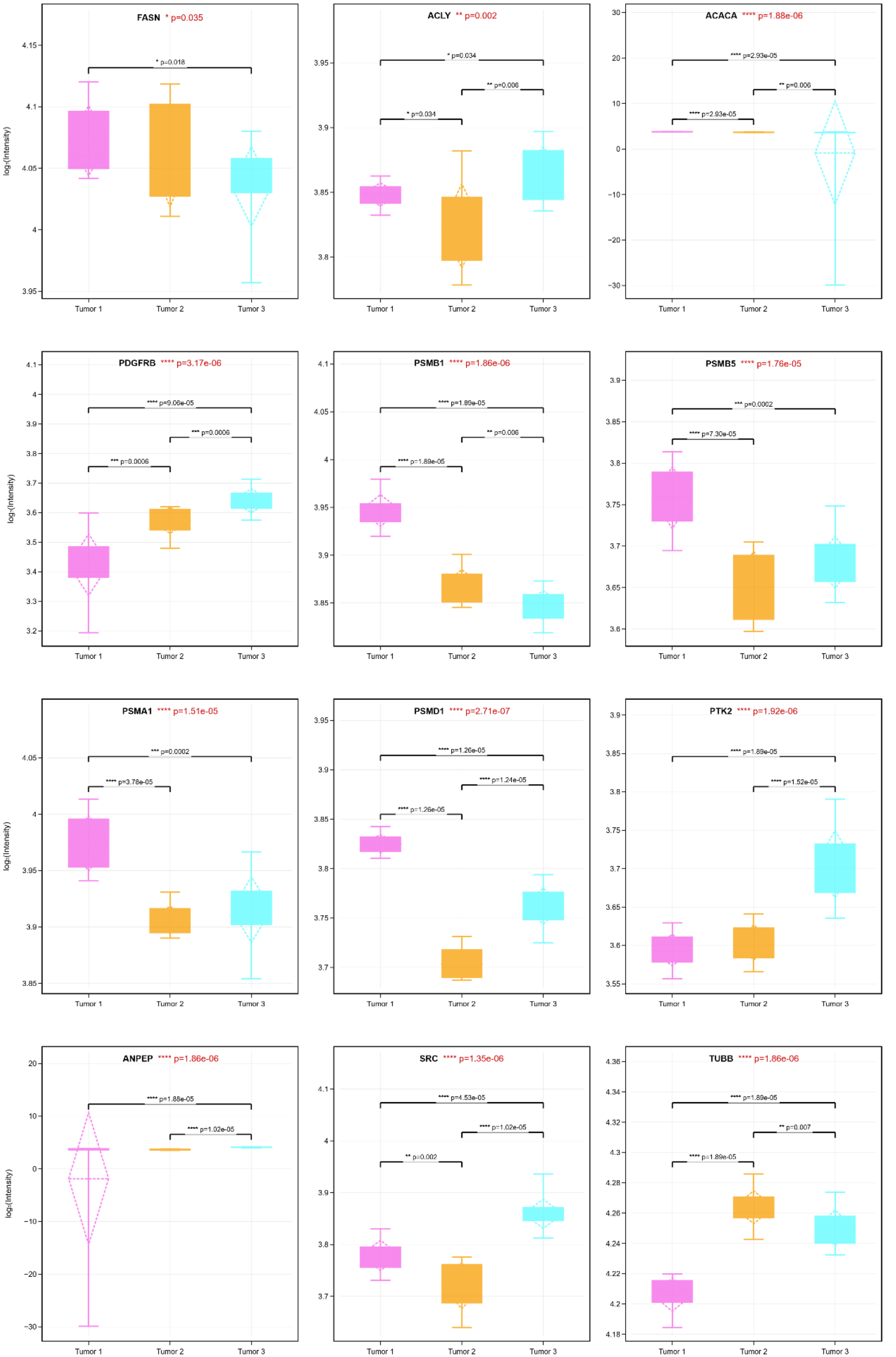

FASN * p=0.035
* p=0.018
ACLY ** p=0.002
* p=0.034
** p=0.006
* p=0.034
ACACA **** p=1.88e-06
**** p=2.93e-05
** p=0.006
**** p=2.93e-05
PDGFRB **** p=3.17e-06
**** p=9.06e-05
*** p=0.0006
*** p=0.0006
PSMB1 **** p=1.86e-06
**** p=1.89e-05
** p=0.006
**** p=1.89e-05
PSMB5 **** p=1.76e-05
*** p=0.0002
**** p=7.30e-05
PSMA1 **** p=1.51e-05
*** p=0.0002
**** p=3.78e-05
PSMD1 **** p=2.71e-07
**** p=1.26e-05
**** p=1.24e-05
**** p=1.26e-05
PTK2 **** p=1.92e-06
**** p=1.89e-05
**** p=1.52e-05
ANPEP **** p=1.86e-06
**** p=1.88e-05
**** p=1.02e-05
SRC **** p=1.35e-06
**** p=4.53e-05
**** p=1.02e-05
** p=0.002
TUBB **** p=1.86e-06
**** p=1.89e-05
** p=0.007
**** p=1.89e-05
log₂(Intensity)
Tumor 1
Tumor 2
Tumor 3

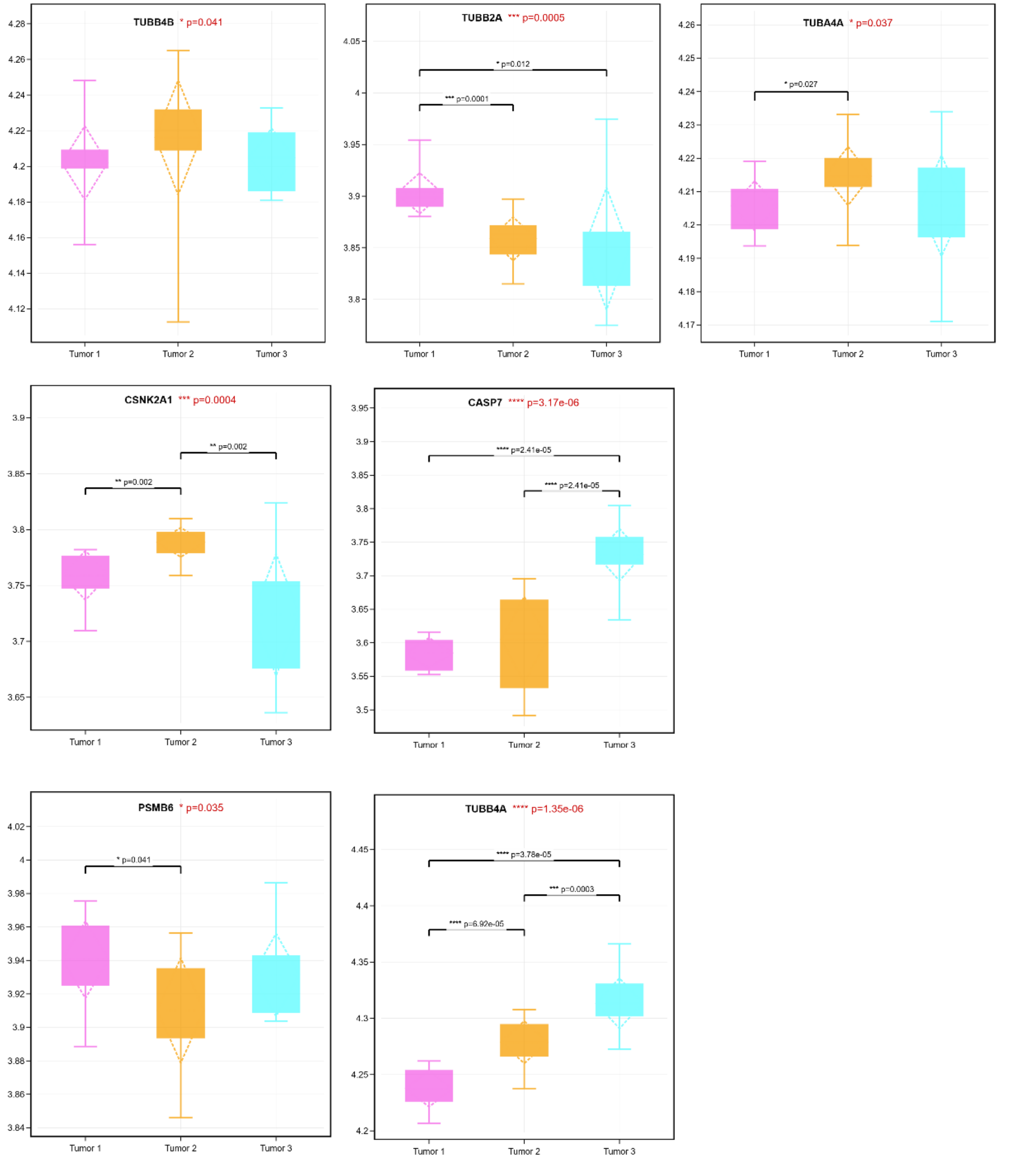


**Figure S10: Protein target enrichment in the tissues from tumors 1, 2 and 3.**

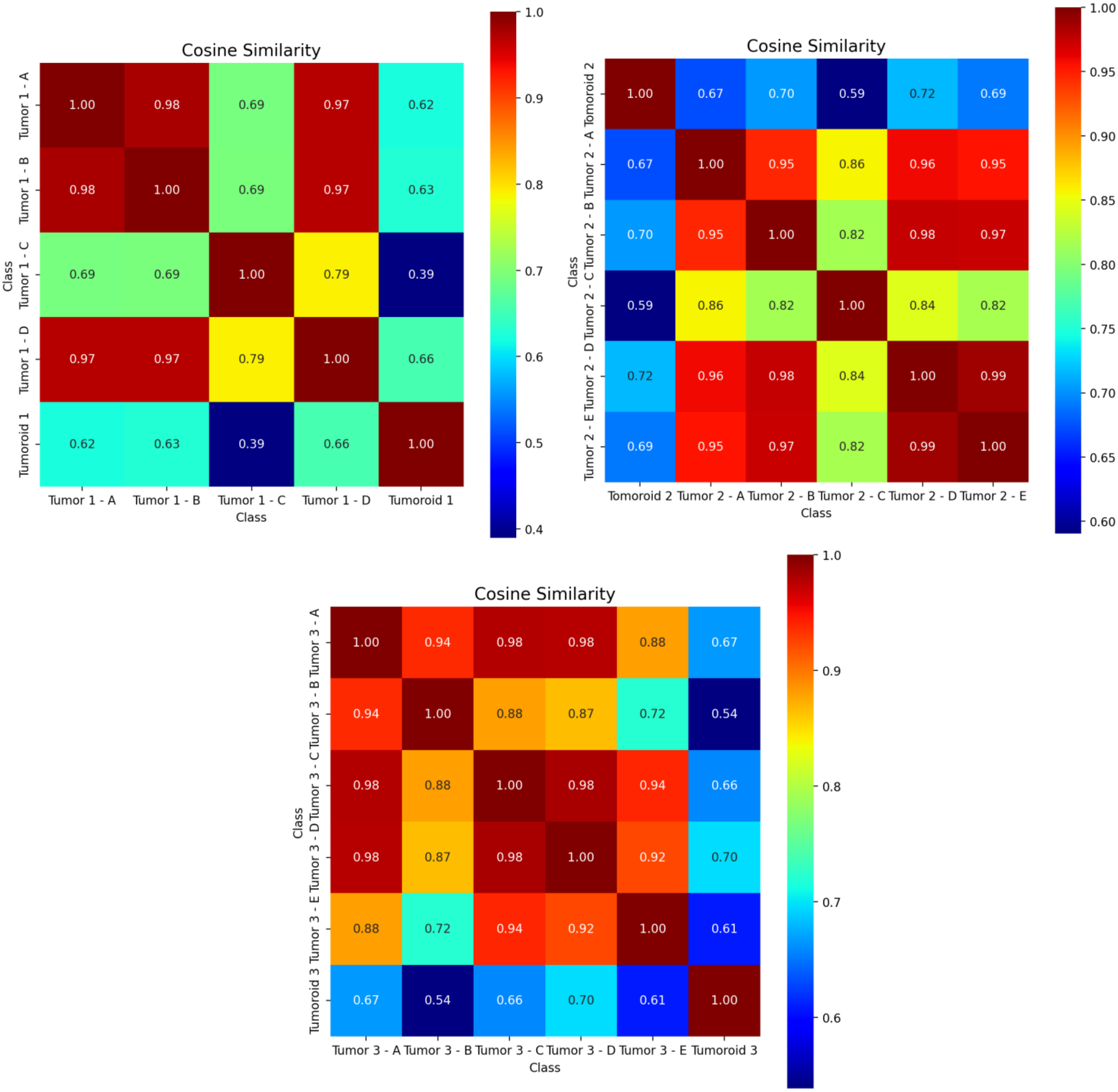


**Figure S11: Proteomic similarity between tumors and paired tumoroids.** Cosine similarity between tumoroids and their matched tumor protein extraction areas for each tumor case, calculated after data preprocessing.

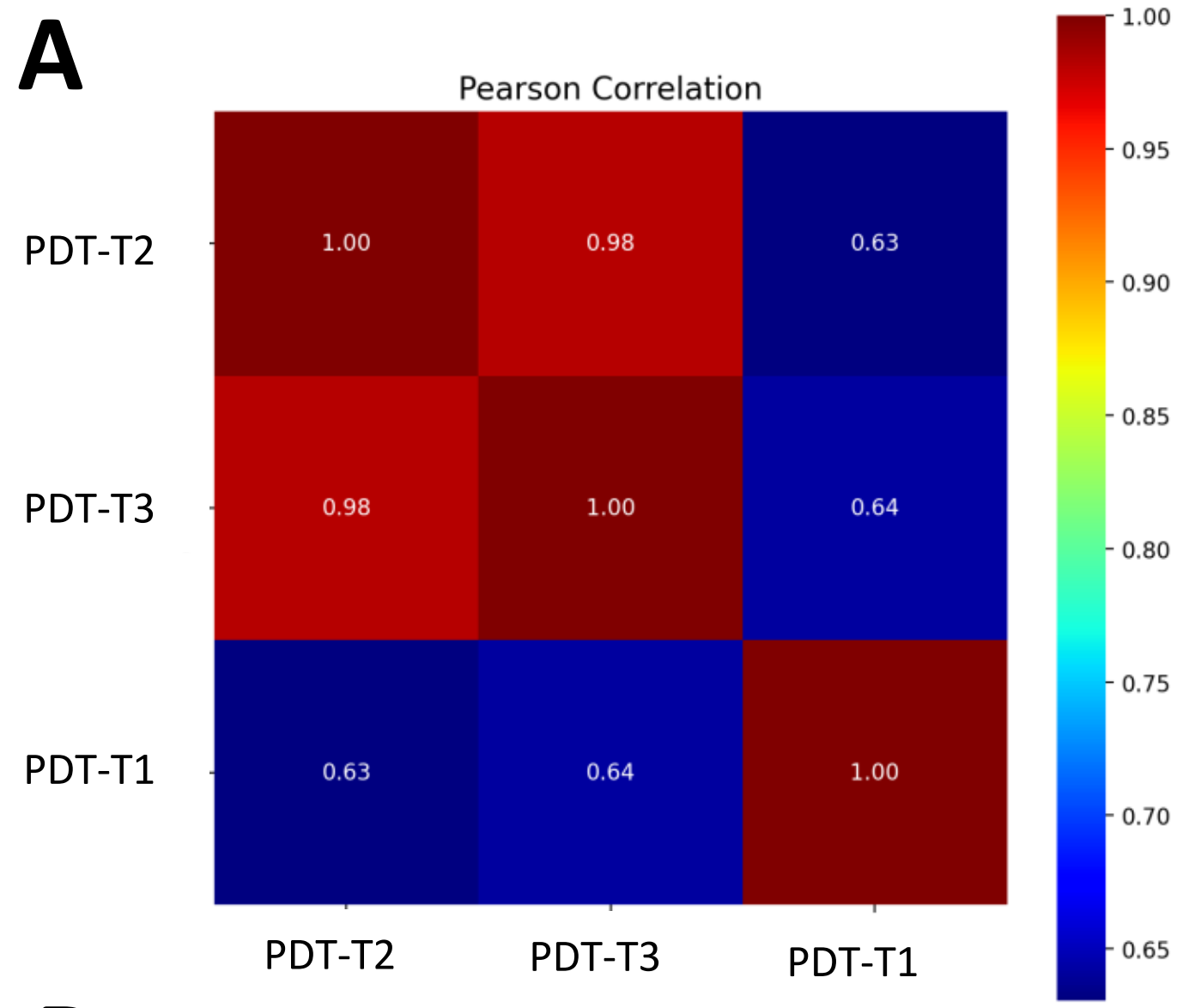


**Figure S12: Spatial and Molecular Characterization of Inter-tumoroid Heterogeneity.** Panel (A) Pearson correlation. Panel (B) compares the overlap and uniqueness of molecular features across four classes using a Venn diagram, suggesting both shared and class-specific elements. Panel (C) visualizes gene expression patterns through ANOVA (p-value < 0,01), uncovering over-expressed proteins in red and relationships among samples or protein genes. Finally, Panel (D) displays pathway enrichment scores across the overexpressed proteins from the four classes, identifying biologically significant pathways that may drive tumor behavior in each tumor.

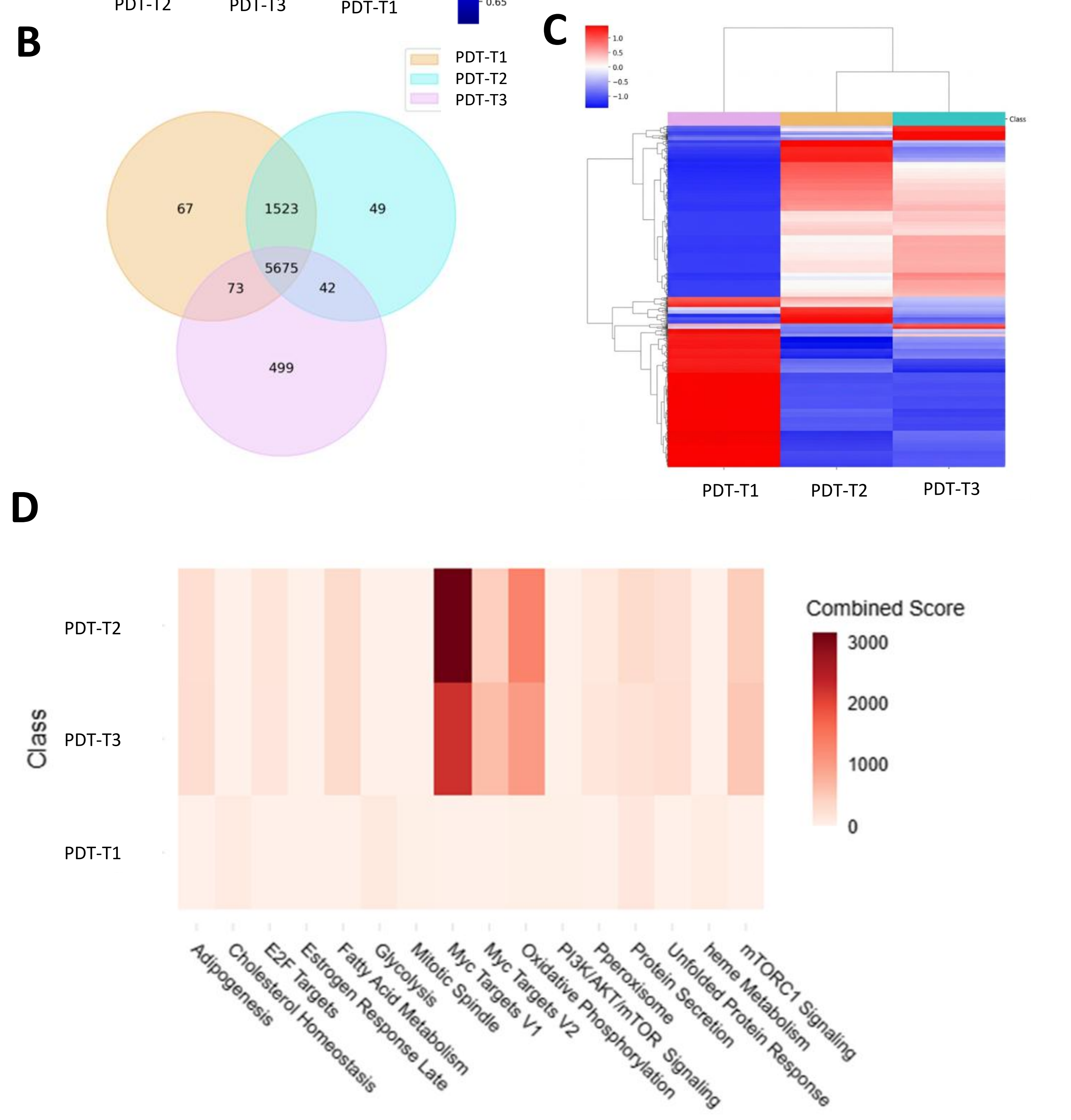

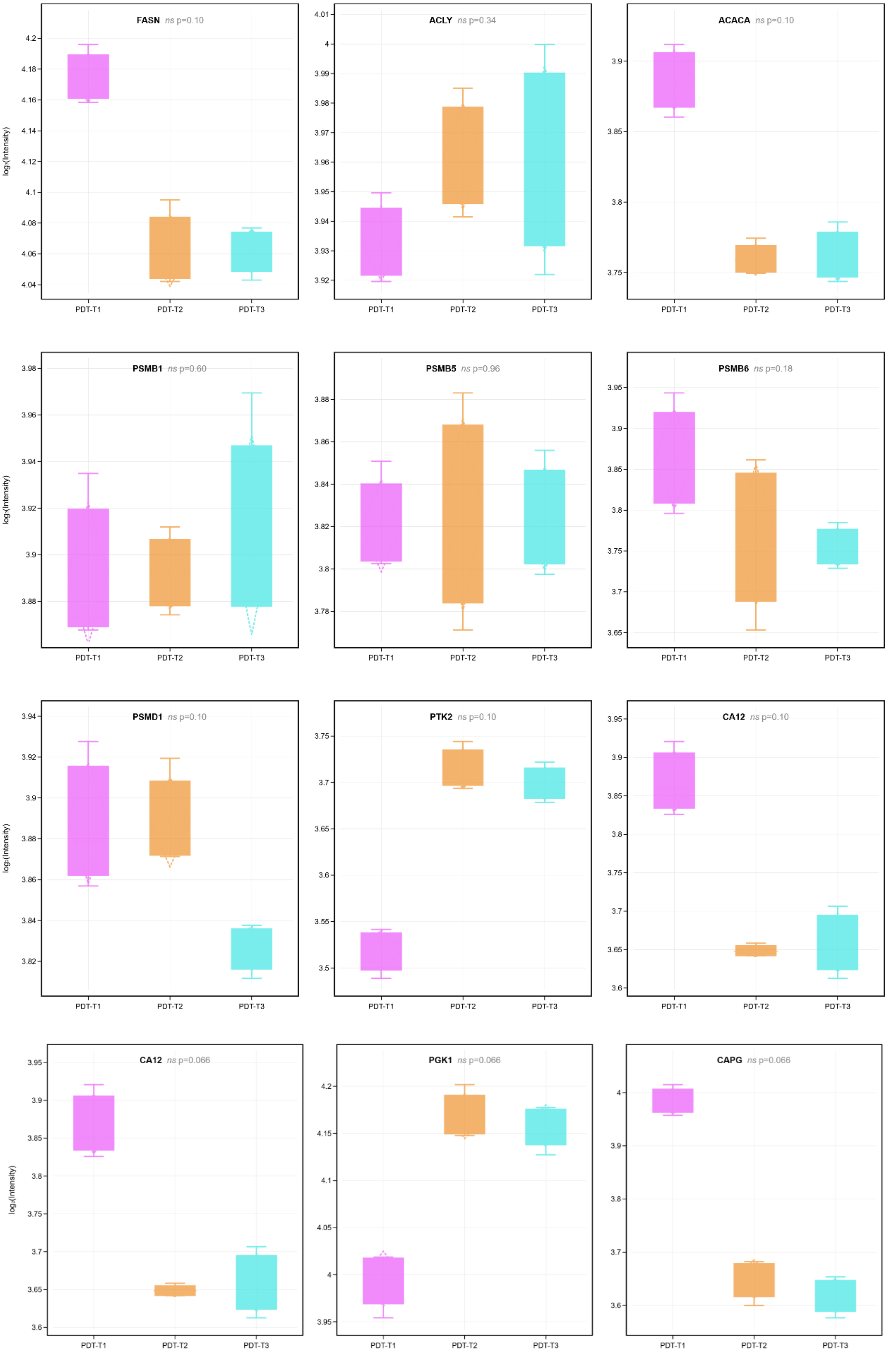

FASN ns p=0.10
log₂(Intensity)
4.2
4.18
4.16
4.14
4.12
4.1
4.08
4.06
4.04
PDT-T1
PDT-T2
PDT-T3
ACLY ns p=0.34
4.01
4
3.99
3.98
3.97
3.96
3.95
3.94
3.93
3.92
PDT-T1
PDT-T2
PDT-T3
ACACA ns p=0.10
3.9
3.85
3.8
3.75
PDT-T1
PDT-T2
PDT-T3
PSMB1 ns p=0.60
log₂(Intensity)
3.98
3.96
3.94
3.92
3.9
3.88
PDT-T1
PDT-T2
PDT-T3
PSMB5 ns p=0.96
3.88
3.86
3.84
3.82
3.8
3.78
PDT-T1
PDT-T2
PDT-T3
PSMB6 ns p=0.18
3.95
3.9
3.85
3.8
3.75
3.7
3.65
PDT-T1
PDT-T2
PDT-T3
PSMD1 ns p=0.10
log₂(Intensity)
3.94
3.92
3.9
3.88
3.86
3.84
3.82
PDT-T1
PDT-T2
PDT-T3
PTK2 ns p=0.10
3.75
3.7
3.65
3.6
3.55
3.5
PDT-T1
PDT-T2
PDT-T3
CA12 ns p=0.10
3.95
3.9
3.85
3.8
3.75
3.7
3.65
3.6
PDT-T1
PDT-T2
PDT-T3
CA12 ns p=0.066
log₂(Intensity)
3.95
3.9
3.85
3.8
3.75
3.7
3.65
3.6
PDT-T1
PDT-T2
PDT-T3
PGK1 ns p=0.066
4.2
4.15
4.1
4.05
4
3.95
PDT-T1
PDT-T2
PDT-T3
CAPG ns p=0.066
4
3.9
3.8
3.7
3.6
PDT-T1
PDT-T2
PDT-T3

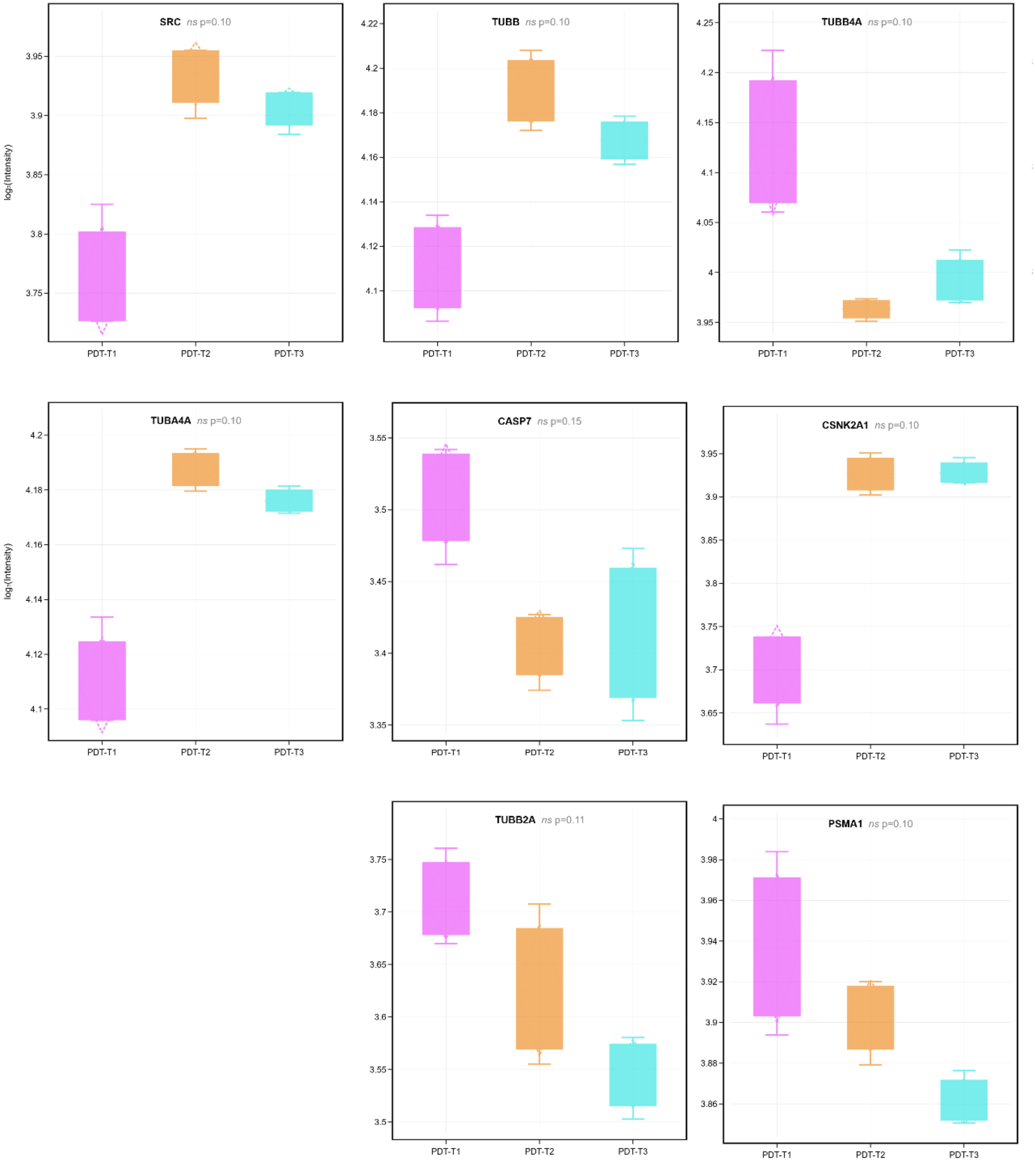


**Figure S13: Protein target enrichment in PDT derived from tumor 1, 2 or 3.**

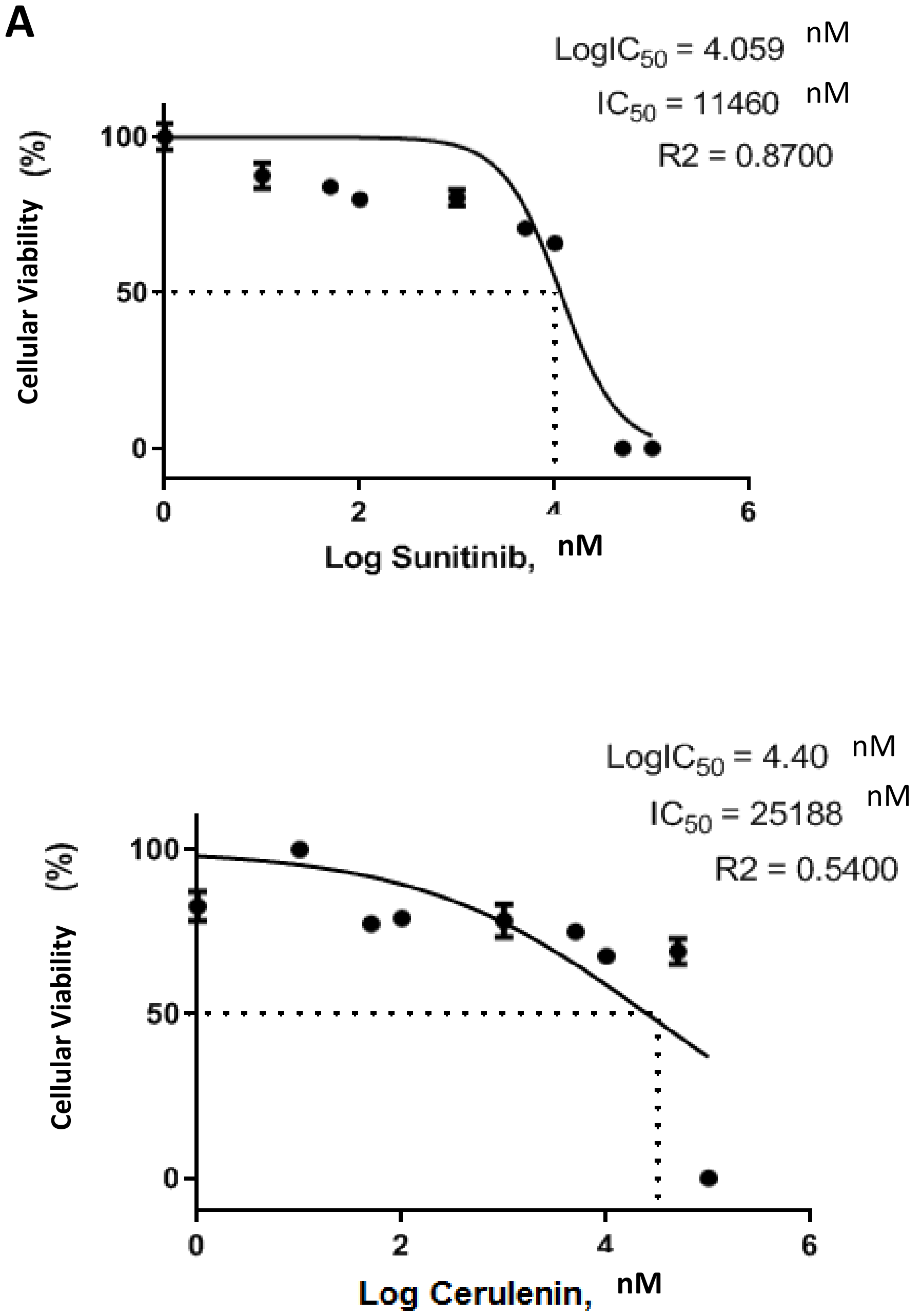
A
LogIC50 = 4.059 nM
IC50 = 11460 nM
R2 = 0.8700
Cellular Viability (%)
Log Sunitinib, nM
LogIC50 = 4.40 nM
IC50 = 25188 nM
R2 = 0.5400
Cellular Viability (%)
Log Cerulenin, nM

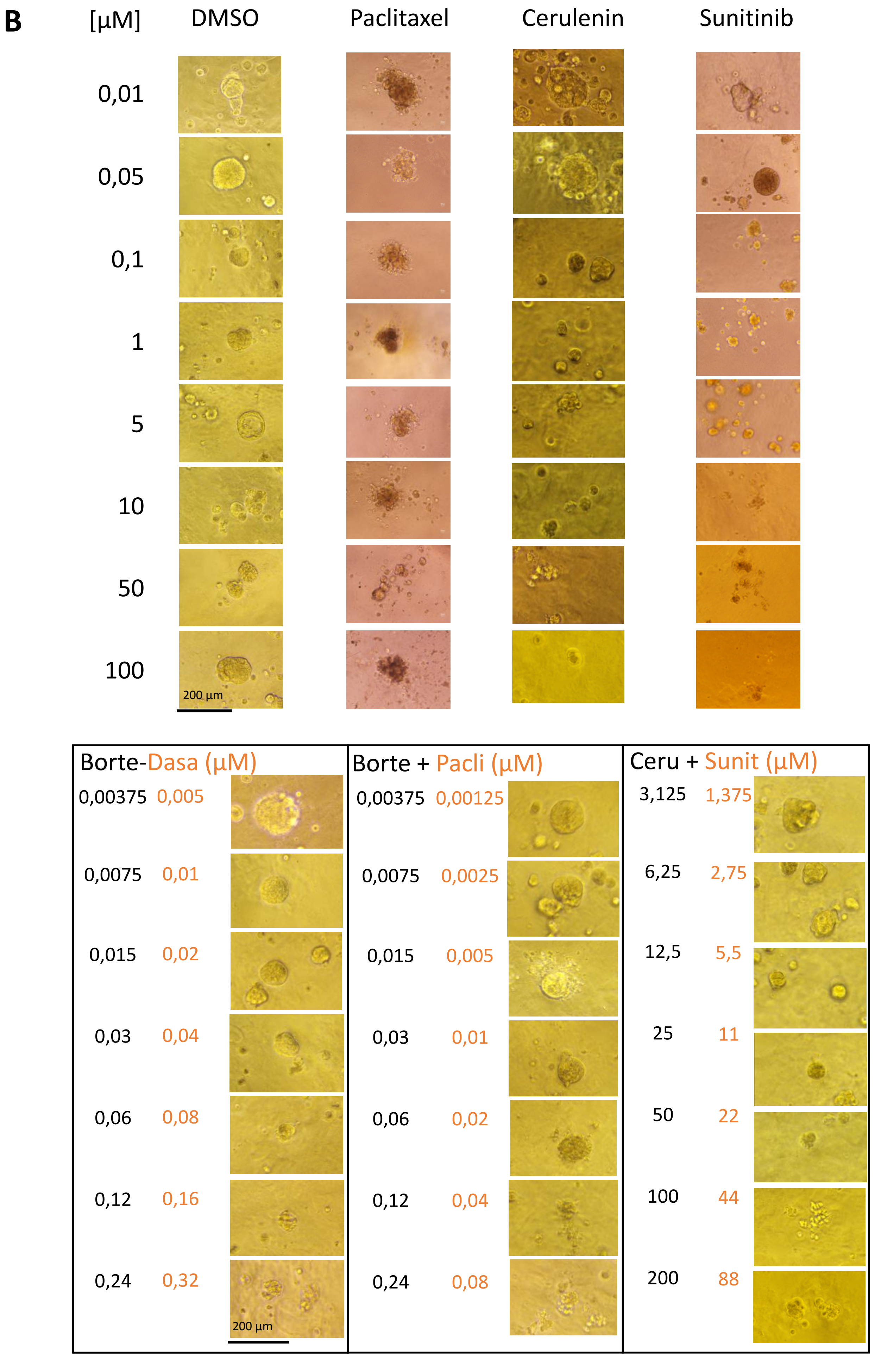


**Figure S14: Tumoroid tumor 1 (A) Viability curves and (B) photos after treatments.**

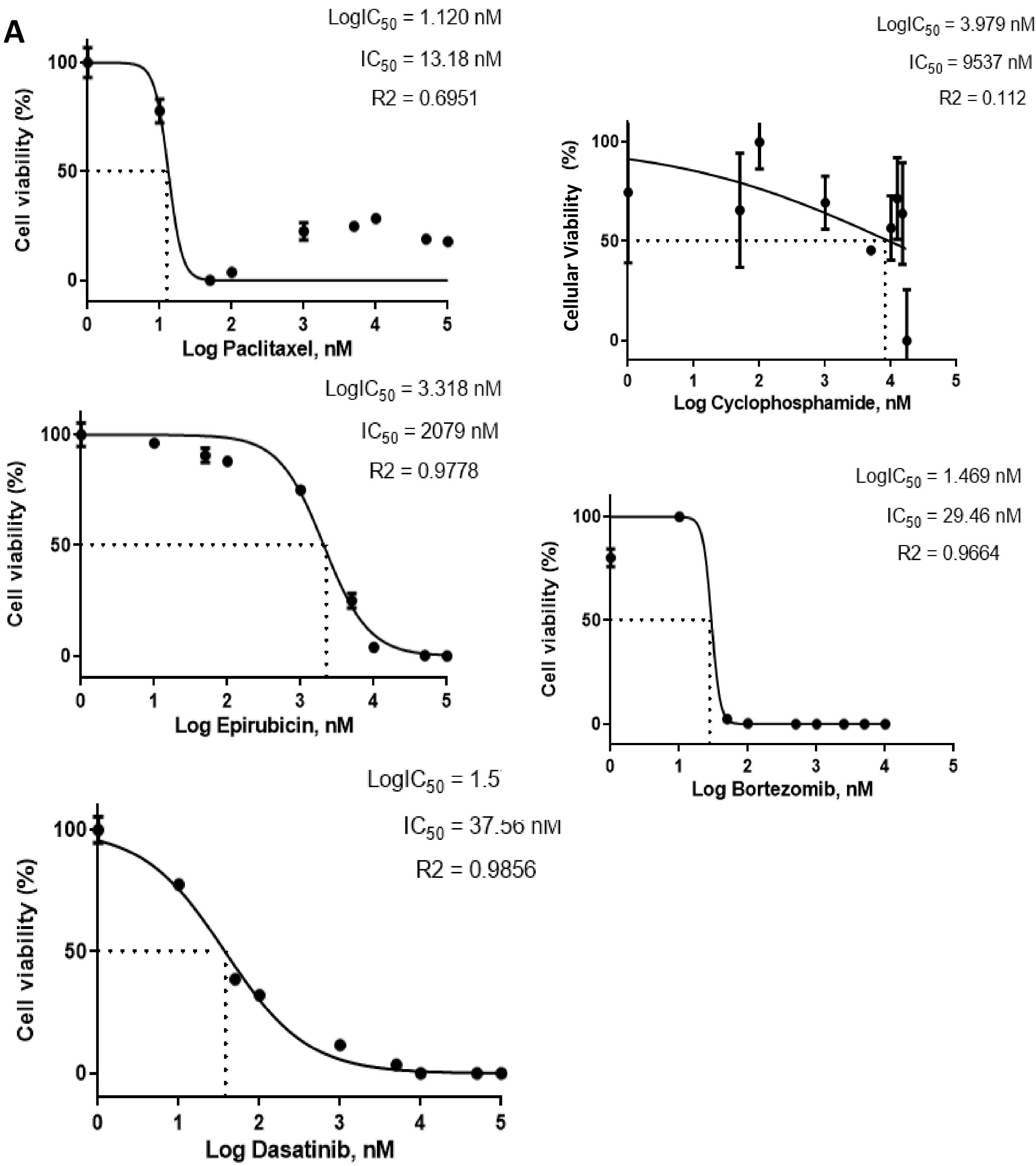
A
LogIC50 = 1.120 nM
IC50 = 13.18 nM
R2 = 0.6951
Cell viability (%)
Log Paclitaxel, nM
LogIC50 = 3.979 nM
IC50 = 9537 nM
R2 = 0.112
Cellular Viability (%)
Log Cyclophosphamide, nM
LogIC50 = 3.318 nM
IC50 = 2079 nM
R2 = 0.9778
Cell viability (%)
Log Epirubicin, nM
LogIC50 = 1.469 nM
IC50 = 29.46 nM
R2 = 0.9664
Cell viability (%)
Log Bortezomib, nM
LogIC50 = 1.5
IC50 = 37.56 nM
R2 = 0.9856
Cell viability (%)
Log Dasatinib, nM

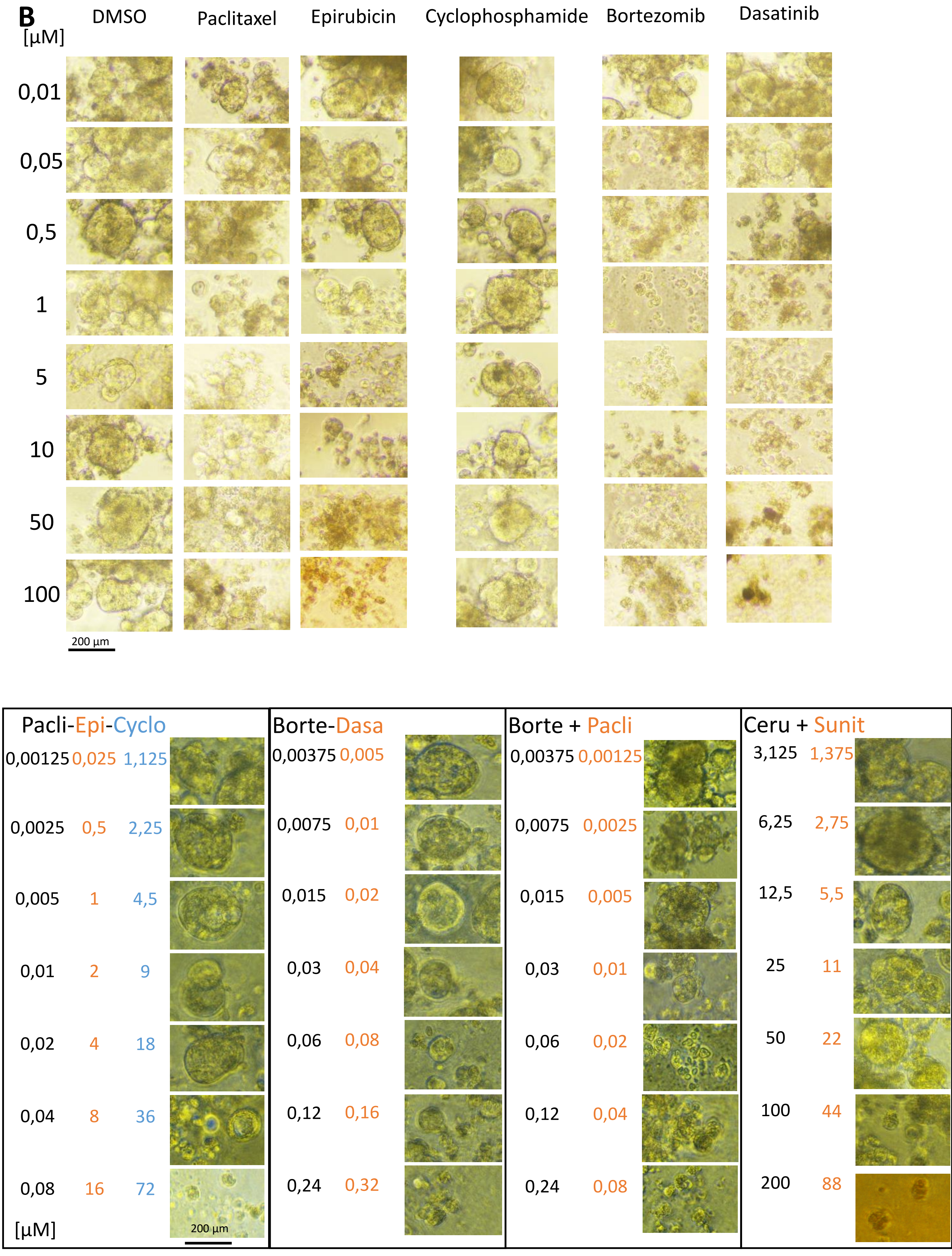


**Figure S15: Tumoroid tumor 2 (A) Viability curves and (B) photos after treatments.**

**A**

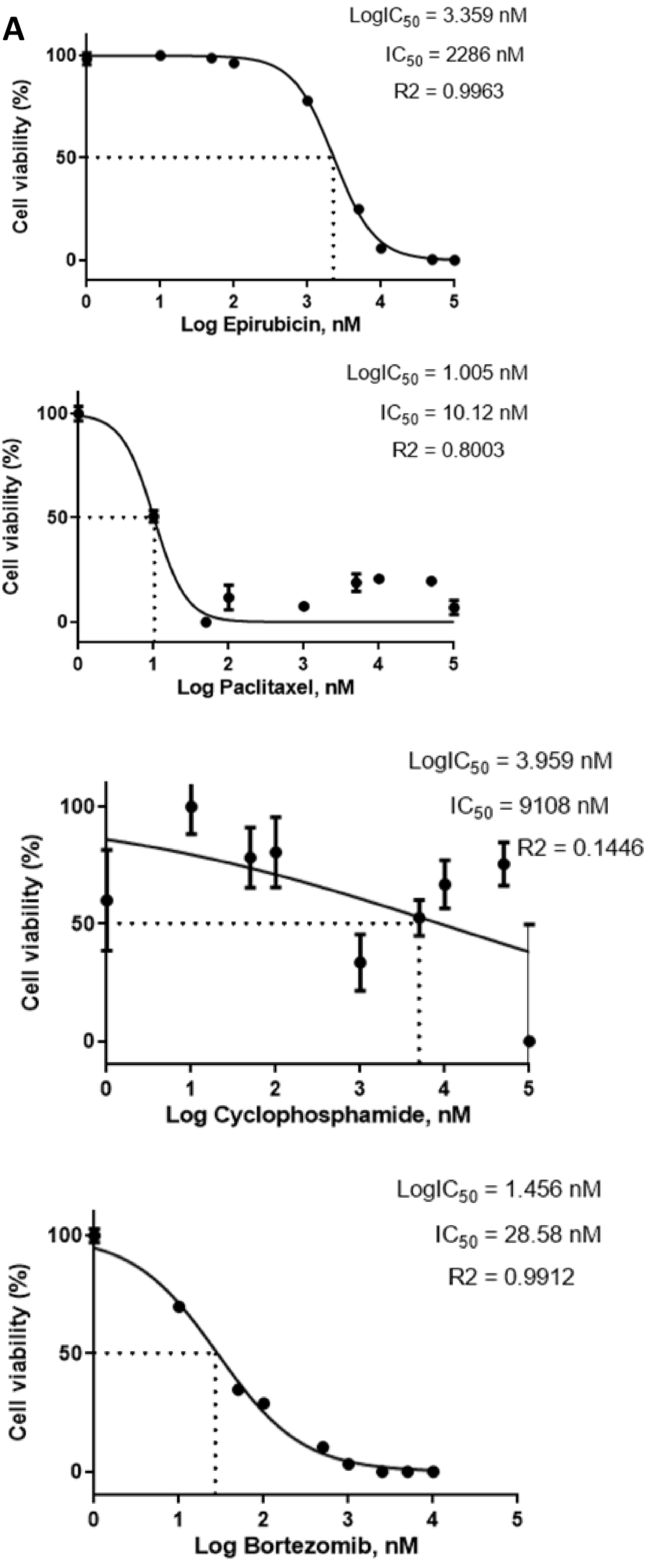
LogIC50 = 3.359 nM
IC50 = 2286 nM
R2 = 0.9963
Cell viability (%)
Log Epirubicin, nM
LogIC50 = 1.005 nM
IC50 = 10.12 nM
R2 = 0.8003
Cell viability (%)
Log Paclitaxel, nM
LogIC50 = 3.959 nM
IC50 = 9108 nM
R2 = 0.1446
Cell viability (%)
Log Cyclophosphamide, nM
LogIC50 = 1.456 nM
IC50 = 28.58 nM
R2 = 0.9912
Cell viability (%)
Log Bortezomib, nM

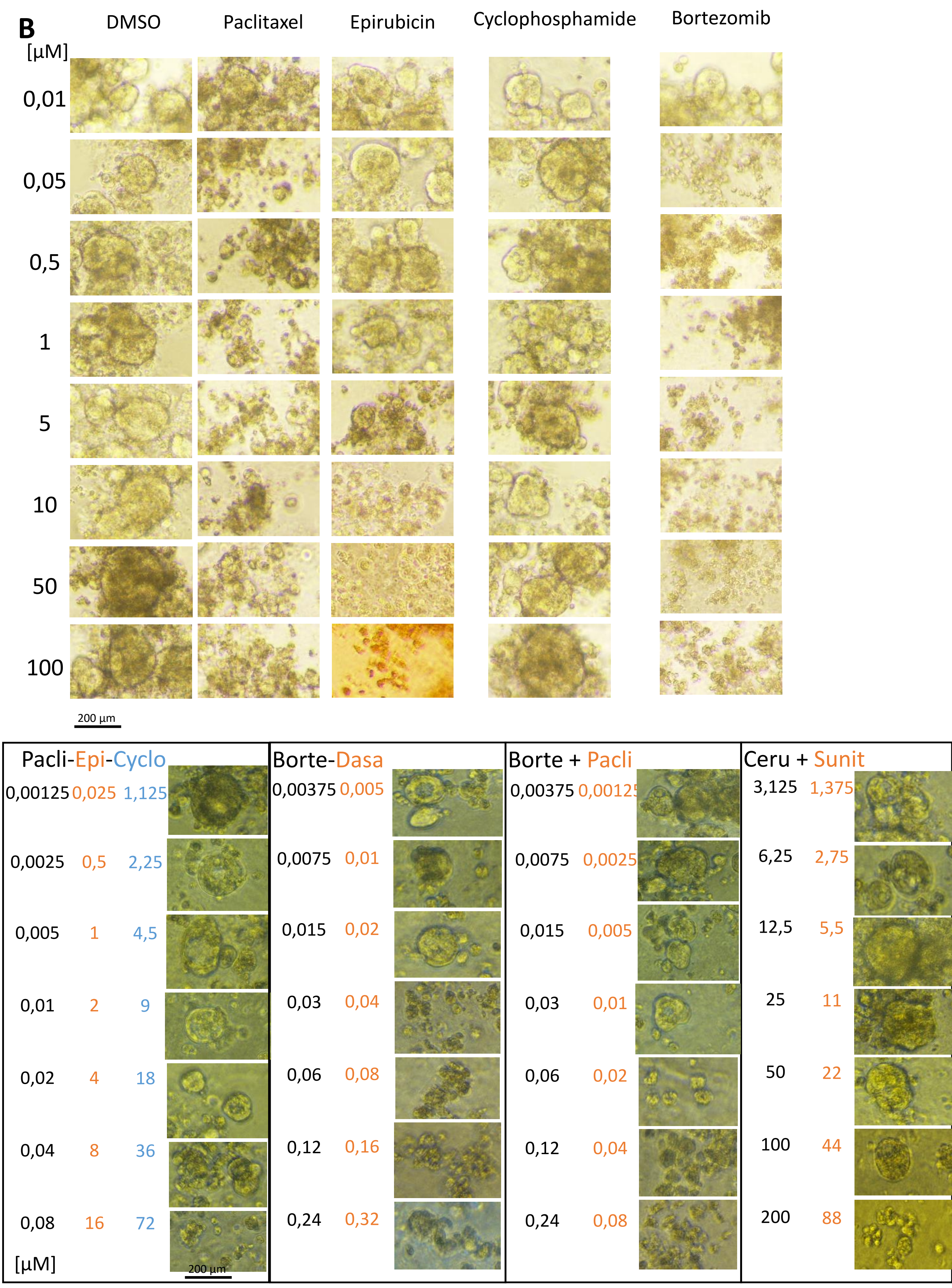


**Figure S16: Tumoroid tumor 3 (A) Viability curves and (B) photos after treatments.**

# Cerulenin + Sunitinib on Tumor1

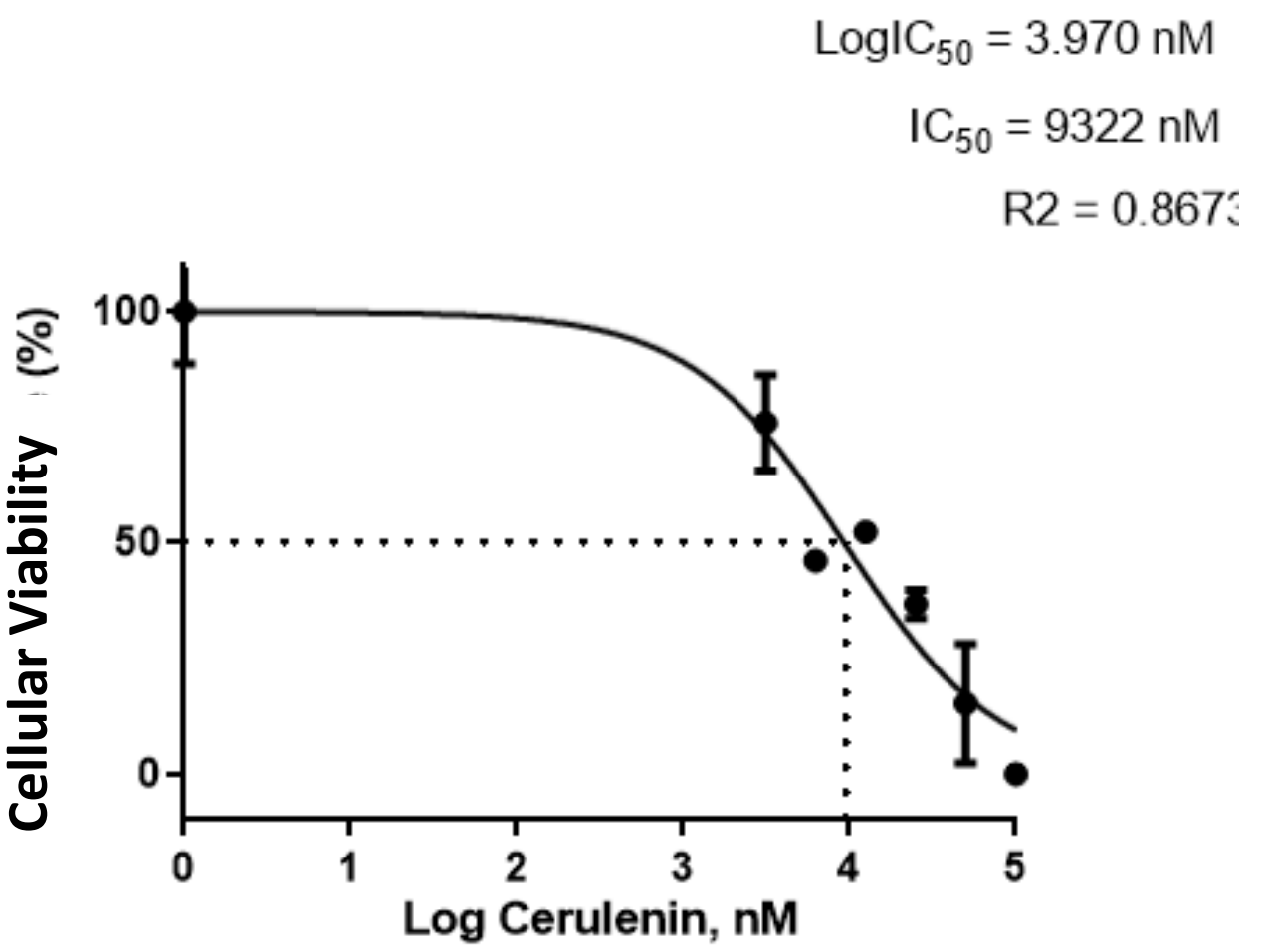


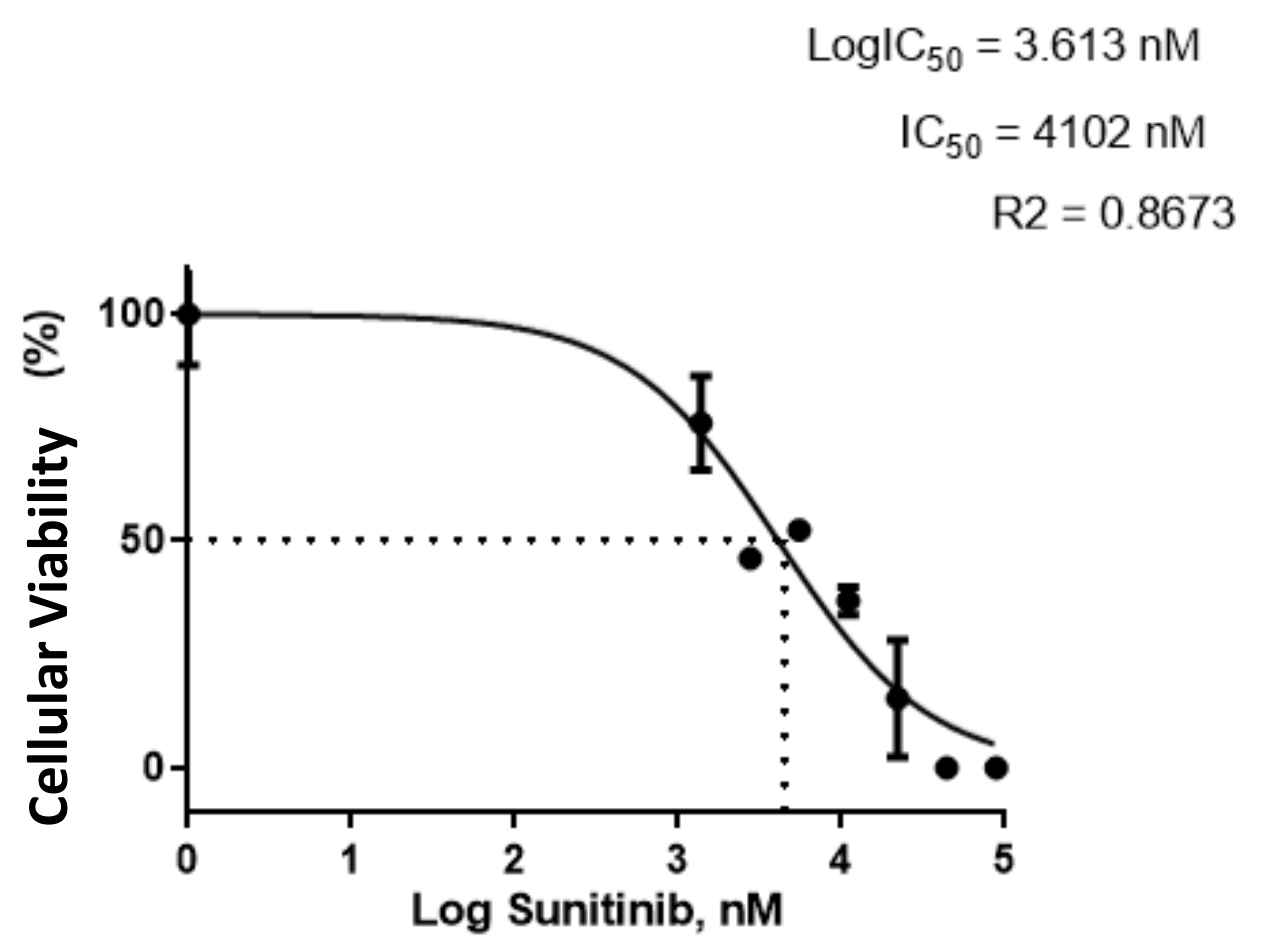


# Cerulenin + Sunitinib on Tumor2

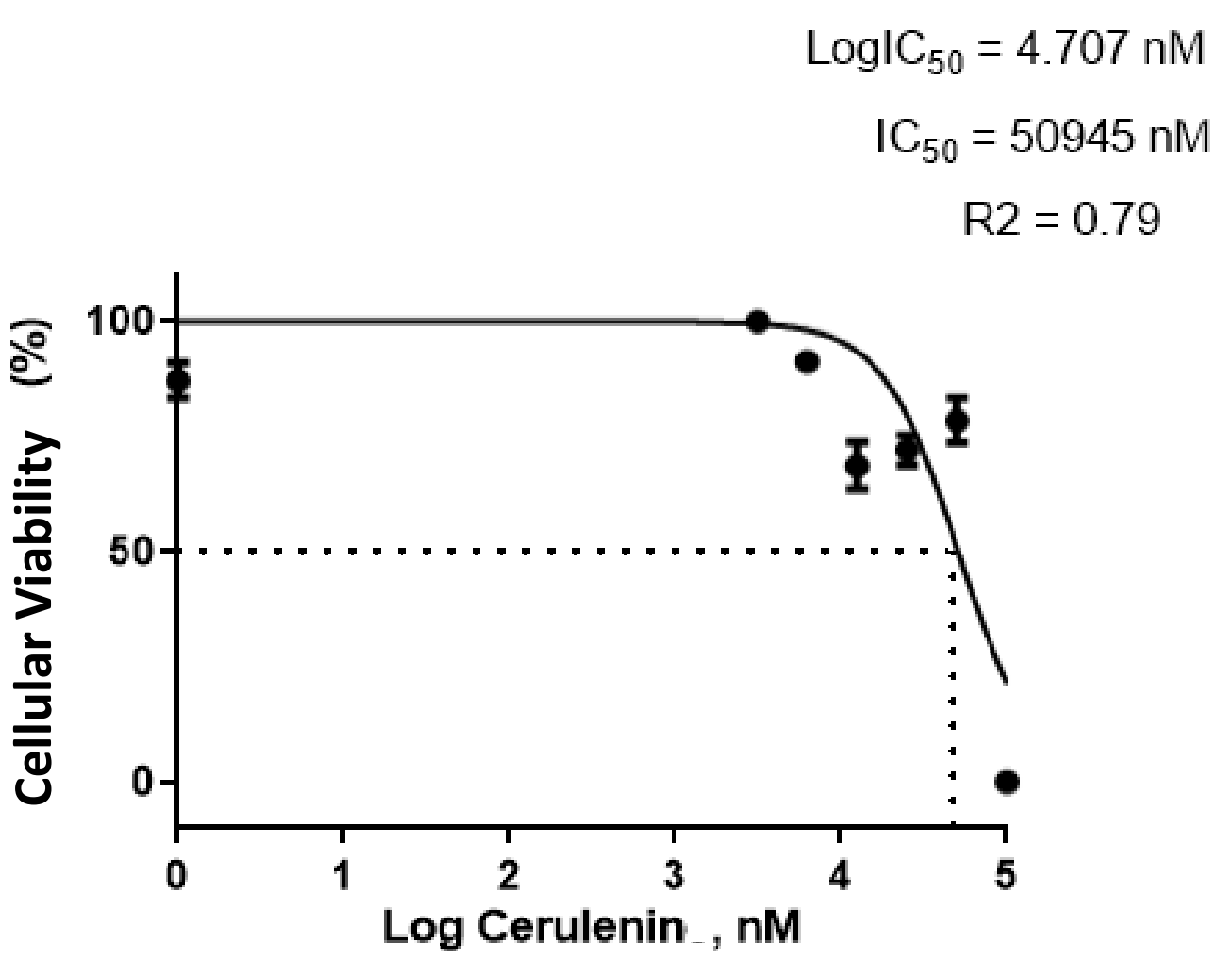


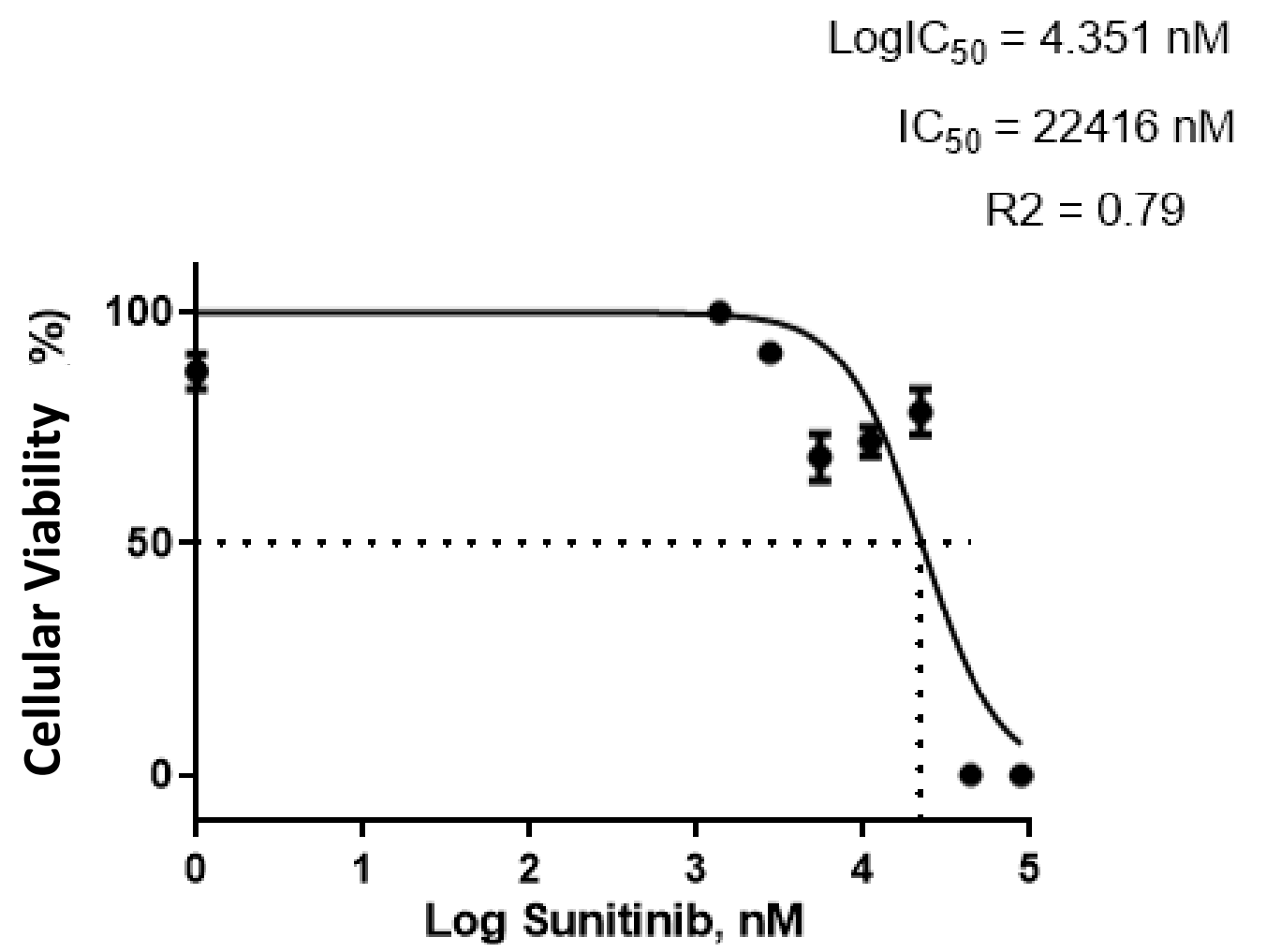


# Cerulenin + Sunitinib on Tumor3

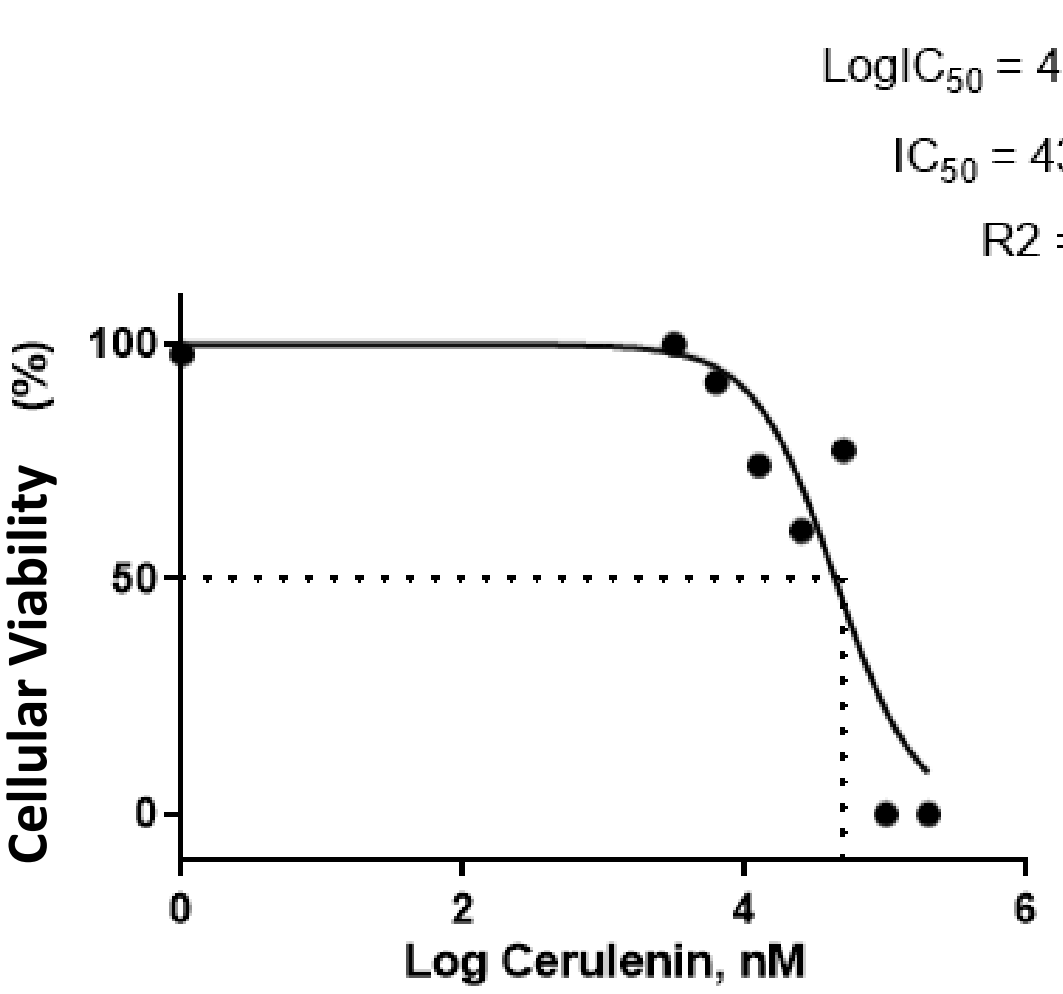


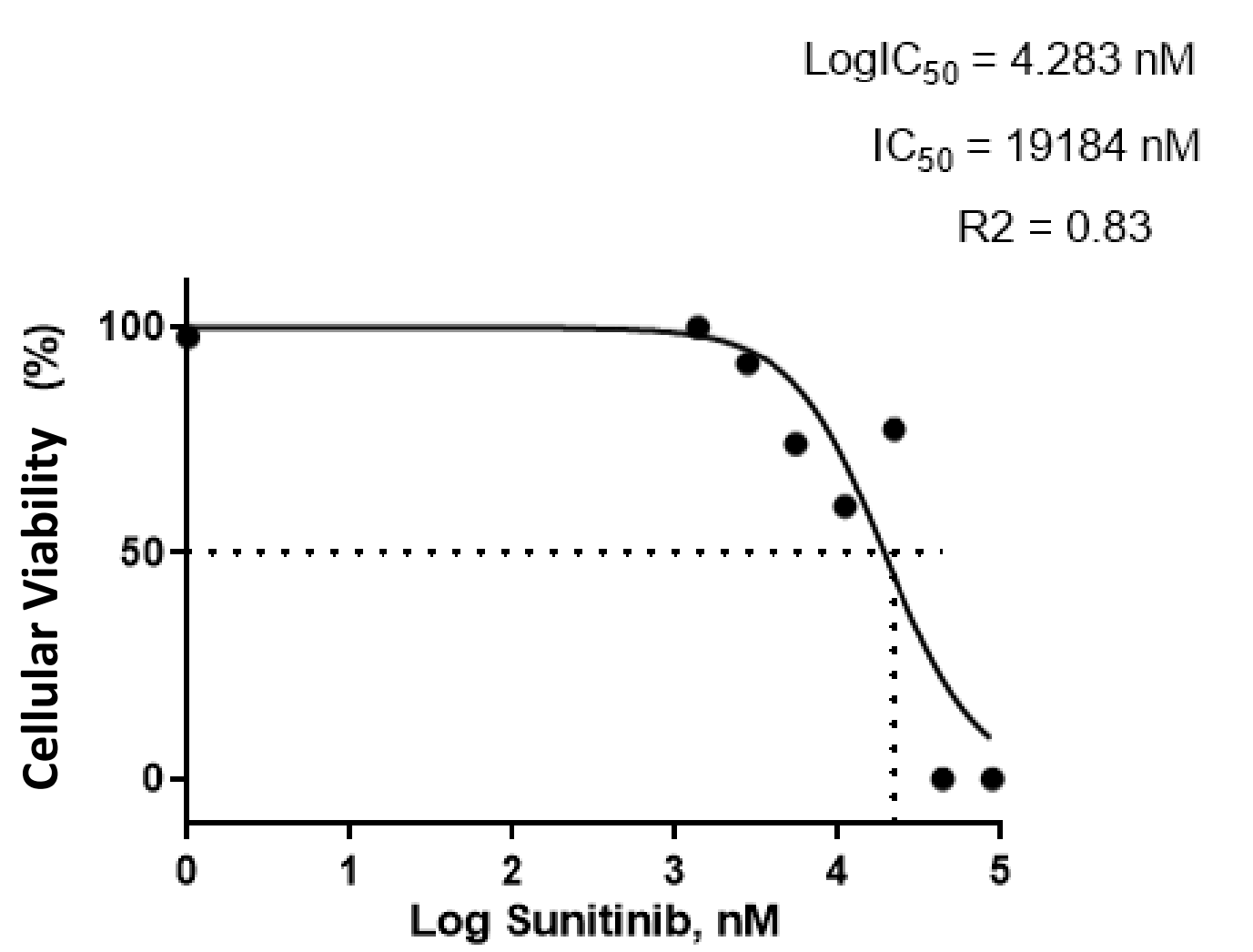


**Figure S17: Cerulenin and sunitinib combinaison (Tumor 1 derived) treatment on tumor 1, 2 and 3,**

## Dasatinib + Bortezomib on Tumor1

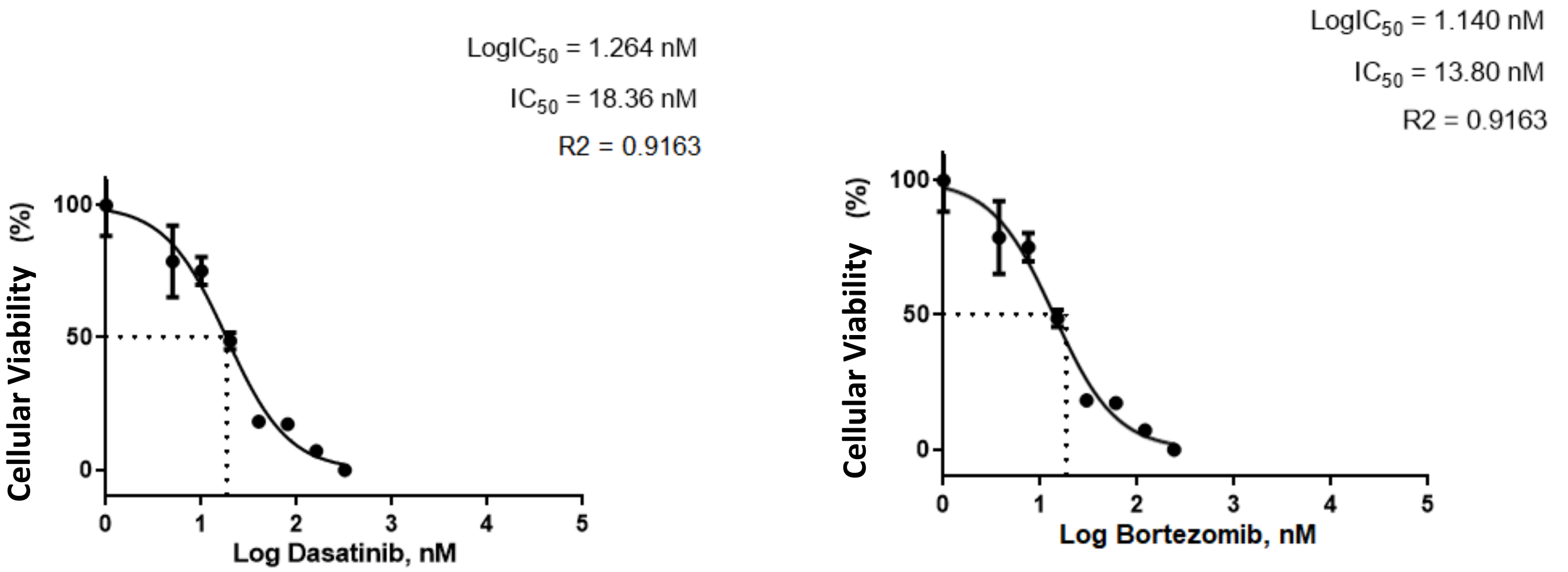


## Dasatinib + Bortezomib on Tumor2

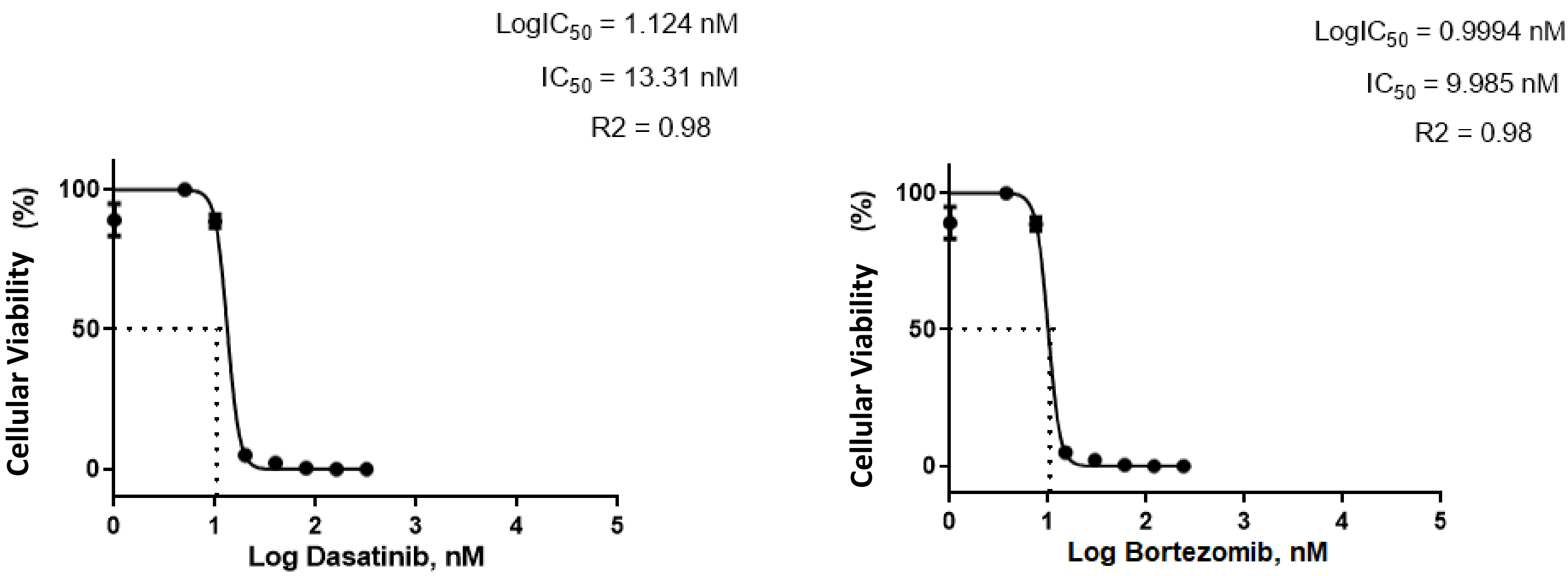


## Dasatinib + Bortezomib on Tumor3

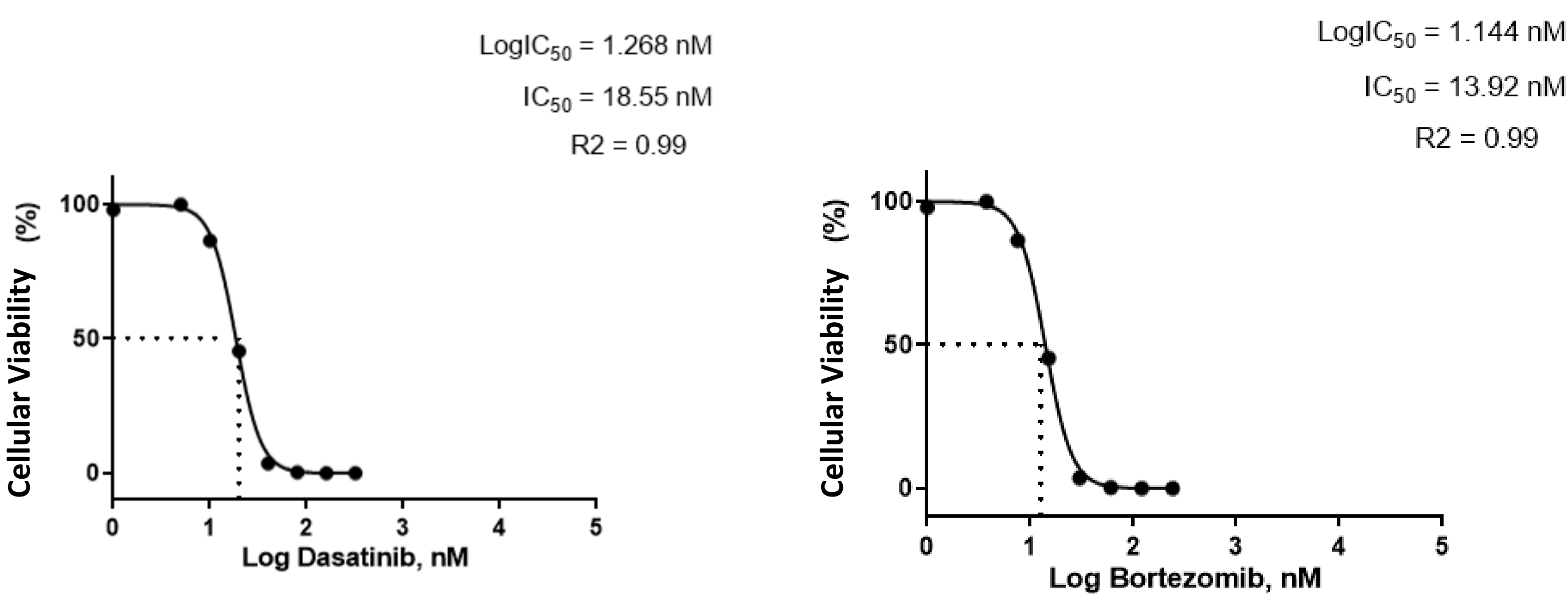


**Figure S18: Dasatinib and Bortezomib combinaison (Tumor 2 derived) treatment on tumor 1, 2 and 3,**

# Paclitaxel+ Bortezomib on Tumor1

LogIC$_{50}$ = 0.7879 nM
IC$_{50}$ = 6.137 nM
R2 = 0.8738

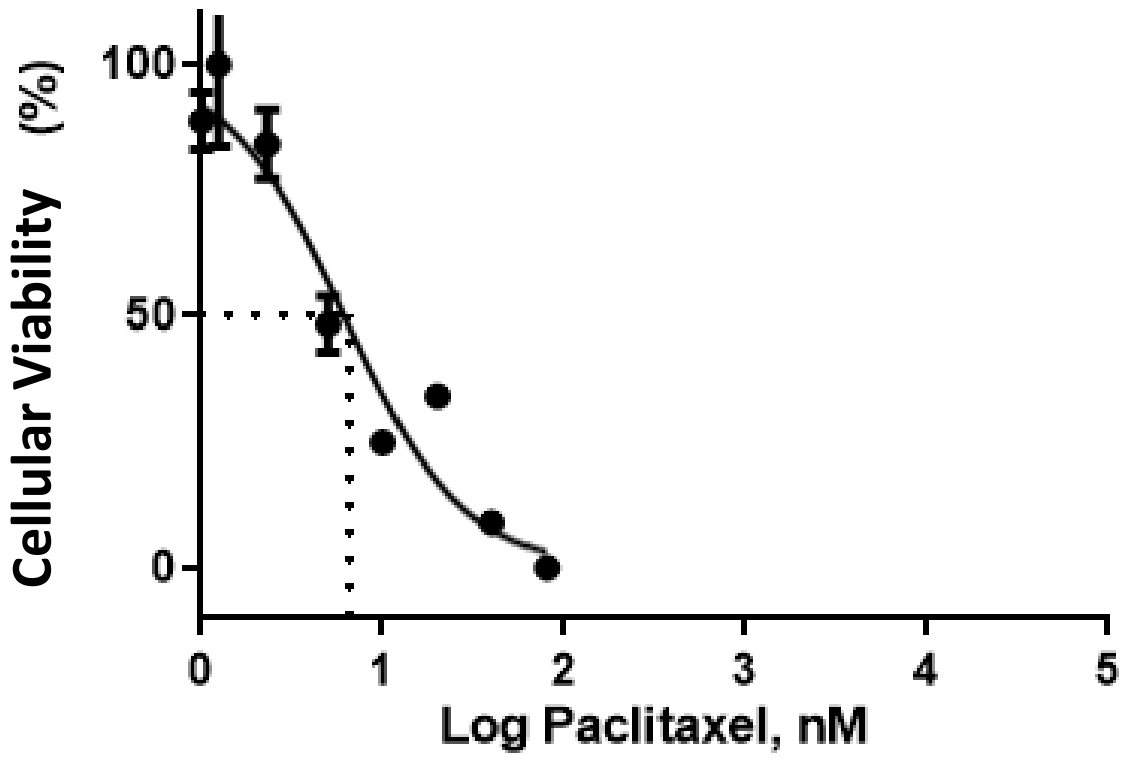


LogIC$_{50}$ = 1.273 nM
IC$_{50}$ = 18.74 nM
R2 = 0.8648

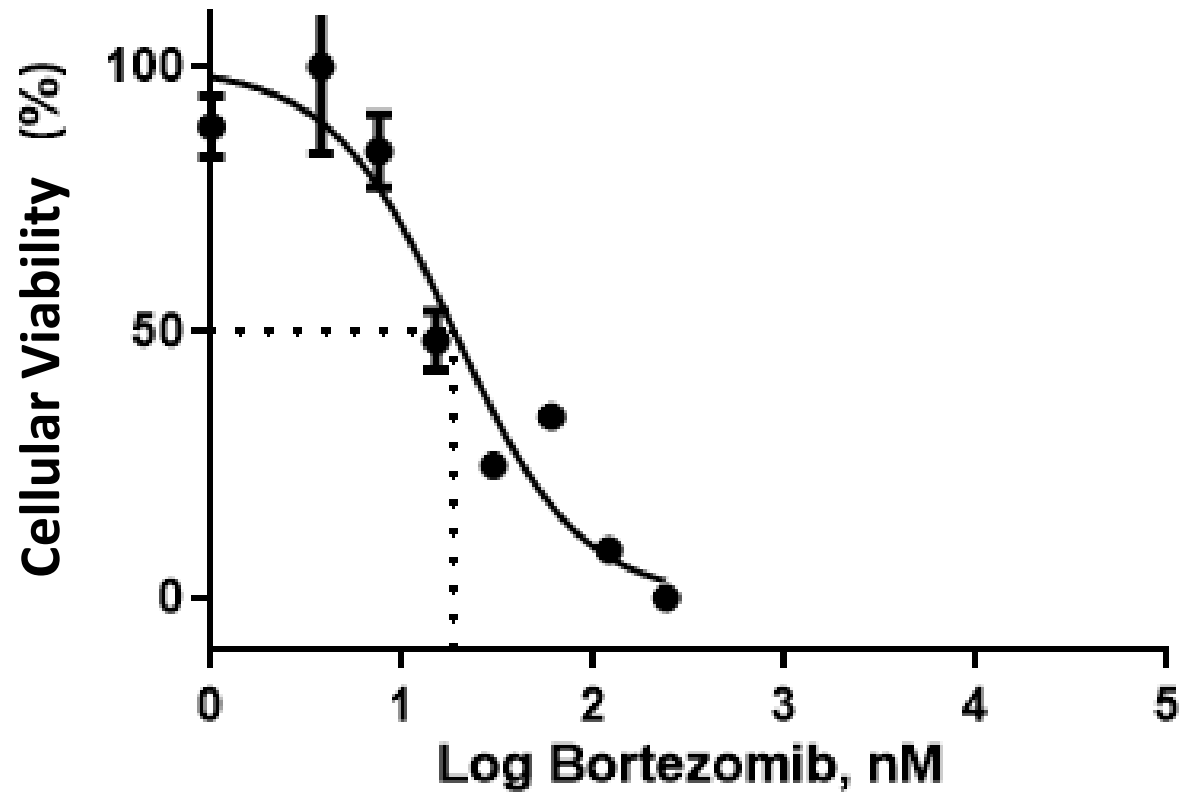


# Paclitaxel+ Bortezomib on Tumor2

LogIC$_{50}$ = 0.6473 nM
IC$_{50}$ = 4.439 nM
R2 = 0.98

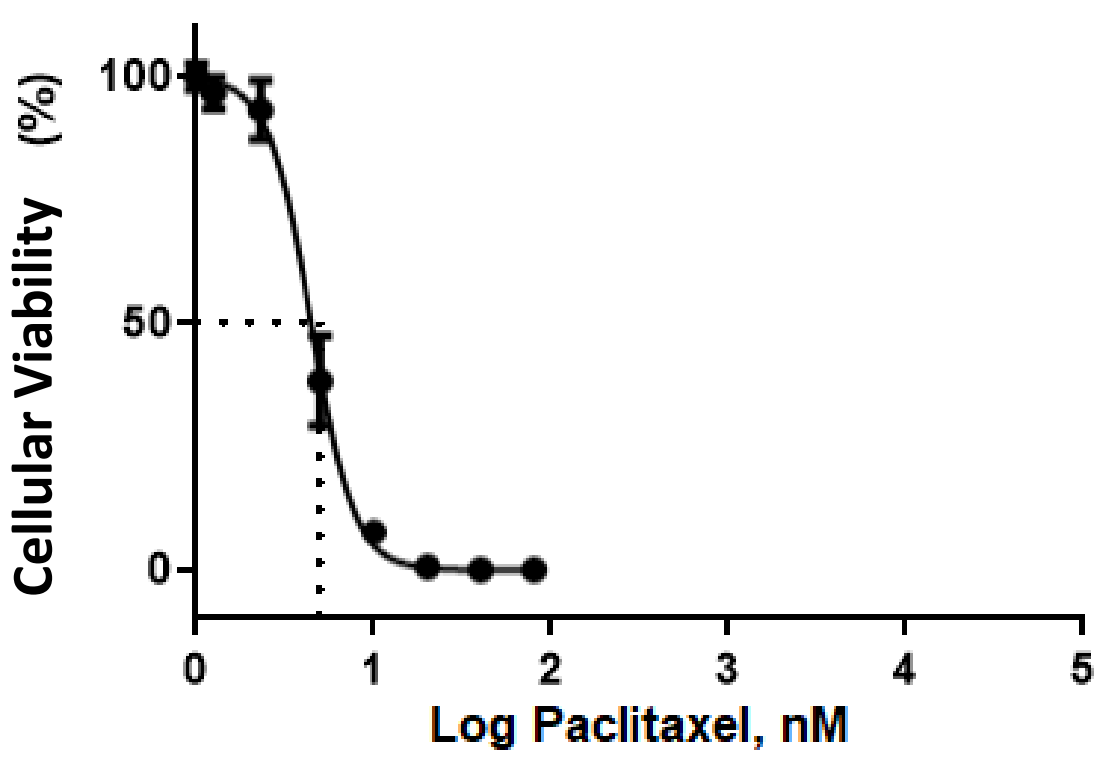


LogIC$_{50}$ = 1.130 nM
IC$_{50}$ = 13.49 nM
R2 = 0.98

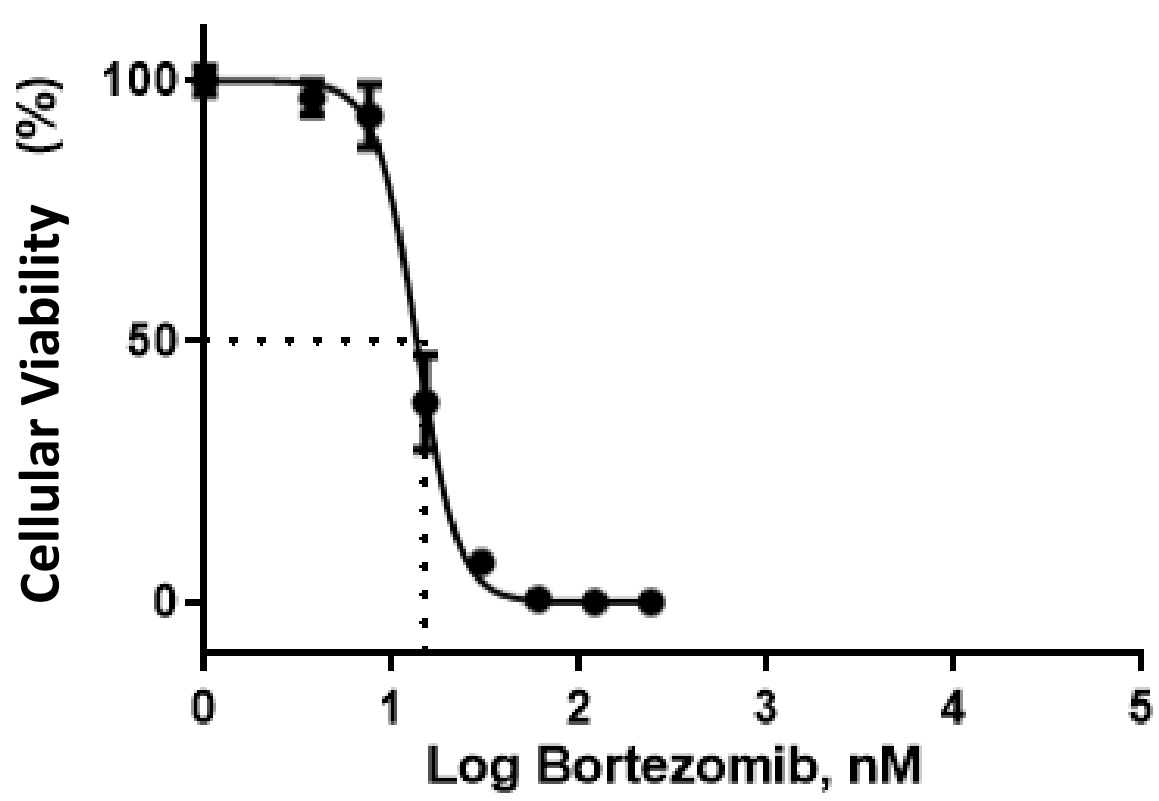


# Paclitaxel+ Bortezomib on Tumor3

LogIC$_{50}$ = 0.9389 nM
IC$_{50}$ = 8.687 nM
R2 = 0.99

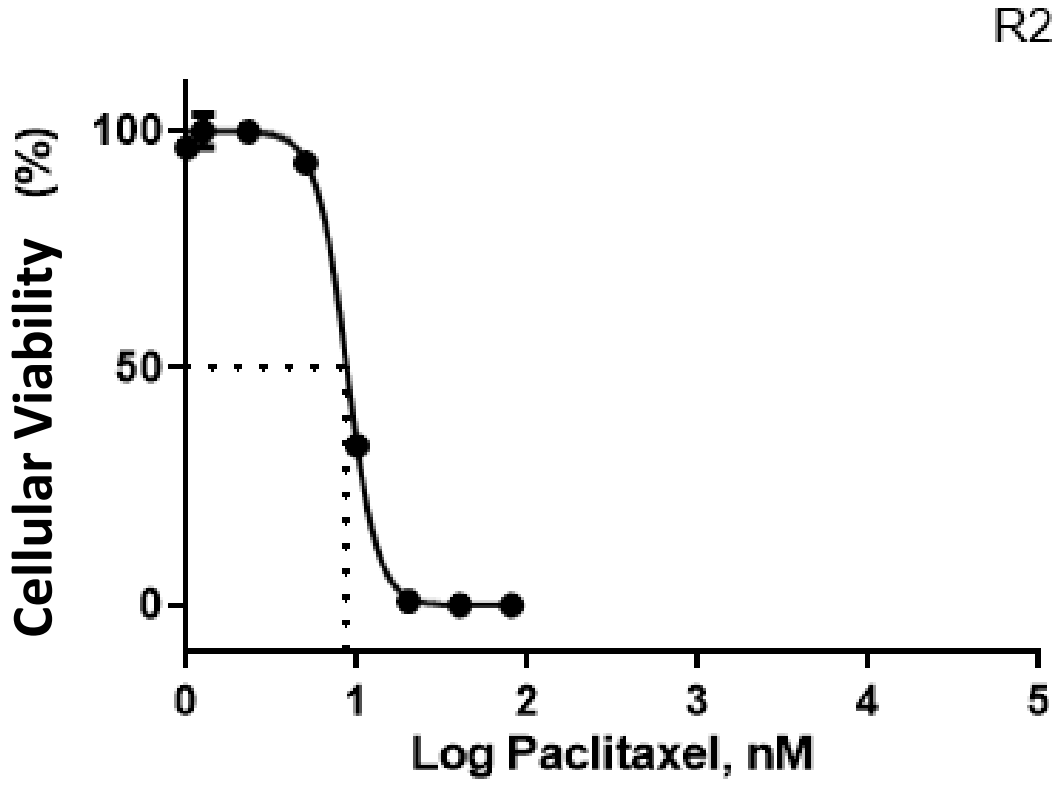


LogIC$_{50}$ = 1.416 nM
IC$_{50}$ = 26.06 nM
R2 = 0.99

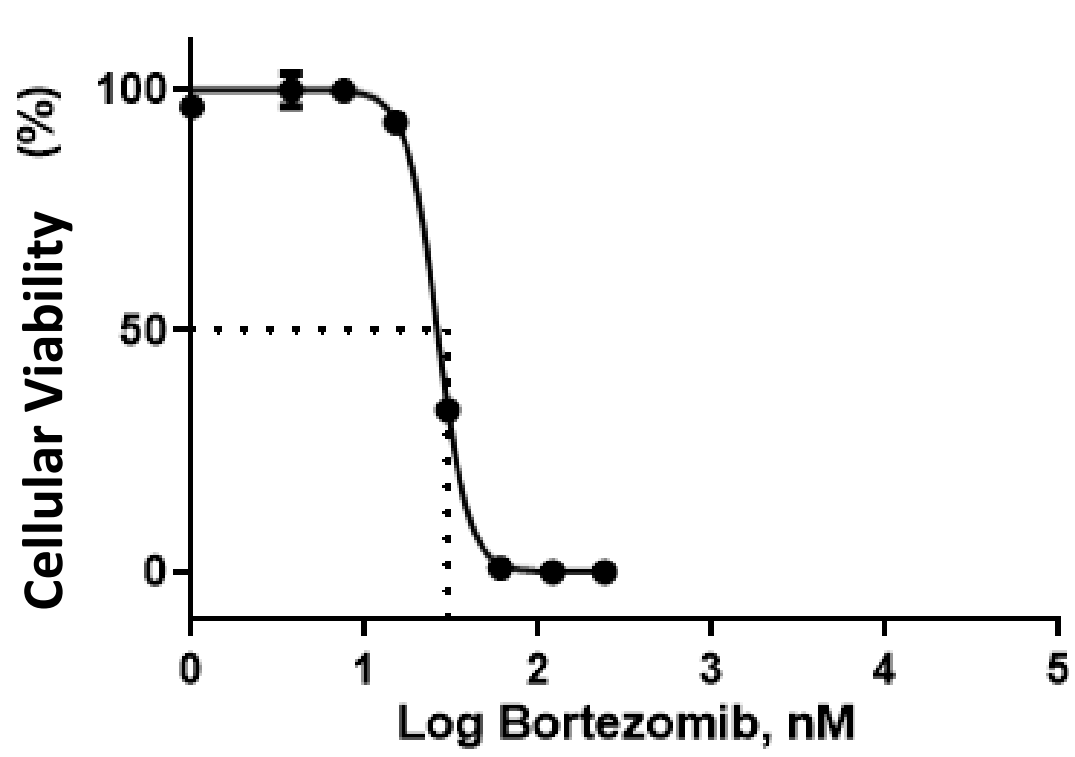


**Figure S19: Paclitaxel and Bortezomib (Tumor 3 derived) treatment on tumor 1, 2 and combinaison 3,**
