# Supplementary material for: Clone-By-Clone Therapy Guidance in Luminal Breast Cancer Via Spatial Proteomics and Patient-Derived Tumoroid
Source: Mol Cell Proteomics. 2026 Aug 12;25(9):101639. doi: 10.1016/j.mcpro.2026.101639 (PMC13579033; doi:10.1016/j.mcpro.2026.101639)
Supplement: Supplementary file 2 [file mmc15.docx]

Clone-by-Clone Therapy Guidance in Luminal Breast Cancer via Spatial Proteomics and Patient-Derived Tumoroid

Laurine Lagache^1^, Antonella Raffo-Romero^1^, Marie Duhamel^1^, Yanis Zirem^1^, Isabelle Fournier^1,3,4*^, Nawale Hajjaji^1,2*^, Michel Salzet^1,3,4*Φ^

^1^ Univ. Lille, Inserm, CHU Lille, U1192, Protéomique Réponse Inflammatoire Spectrométrie de Masse (PRISM), F-59000 Lille, France

^2^ Breast Cancer Unit, Oscar Lambret Center, Lille, France

^3^ Institut Universitaire de France, Ministère de l’Enseignement Supérieur, de la Recherche et de l’Innovation, 1 rue Descartes, 75231 PARIS CEDEX 05

^4^ Equipe Labellisée Ligue Contre le Cancer, Lille, France.

# Supplementary Figure Legends

**Figure S1** : t-SNE visualization of the clusters obtained after segmentation of MSI from A) tumour 1, B) tumour 2 and C) tumour 3.

**Figure S2** : *m/z* comparison spectra between each tumour MSI cluster comprising upset plot and cluster mean spectra for A) Tumour 1, B) Tumour 3 and C) Tumour 2. Upset plot supplementary data available as Supplementary MSI data.

**Figure S3** : Molecular Characterization of Tumour 1 Heterogeneity. Panel (A) Pearson correlation. Panel (B) protein target enrichment across clones.

**Figure S4** : Stromal, immune, and vascular marker proteins across all Tumour 1 clusters. Protein of interest involvement across protein pathways.

**Figure S5** : Molecular Characterization of Tumour 2 Heterogeneity. Panel (A) Pearson correlation. Panel (B) protein target enrichment across clones.

**Figure S6** : Stromal, immune, and vascular marker proteins across all Tumour 2 clusters. Protein of interest involvement across protein pathways.

**Figure S7** : Molecular Characterization of Tumour 3 Heterogeneity. Panel (A) Pearson correlation. Panel (B) protein target enrichment across clones.

**Figure S8** : Stromal, immune, and vascular marker proteins across all Tumour 3clusters. Protein of interest involvement across protein pathways.

**Figure S9** : Spatial and Molecular Characterization of Inter-tumor Heterogeneity. Panel (A) Pearson correlation. Panel (B) Gene count per enriched pathway. Panel (C) pathway enrichment across regions selected from all tumors.

**Figure S10** : Protein target enrichment in the tissues from tumors 1, 2 and 3.

**Figure S11** : Proteomic similarity between tumors and paired tumoroids. Cosine similarity between tumoroids and their matched tumor protein extraction areas for each tumor case, calculated after data preprocessing.

**Figure S12** : Spatial and Molecular Characterization of Inter-tumoroid Heterogeneity. (A) Pearson correlation. (B) Overlap and uniqueness of molecular features across four classes (Venn diagram). (C) Gene expression patterns through ANOVA (p < 0.01). (D) Pathway enrichment scores across overexpressed proteins.

**Figure S13** : Protein target enrichment in PDT derived from tumor 1, 2 or 3.

**Figure S14** : Tumoroid tumor 1 (A) Viability curves and (B) photos after treatments.

**Figure S15** : Tumoroid tumor 2 (A) Viability curves and (B) photos after treatments.

**Figure S16** : Tumoroid tumor 3 (A) Viability curves and (B) photos after treatments.

**Figure S17** : Cerulenin and sunitinib combination (Tumor 1 derived) treatment on tumor 1, 2 and 3.

**Figure S18** : Dasatinib and Bortezomib combination (Tumor 2 derived) treatment on tumor 1, 2 and 3.

**Figure S19** : Paclitaxel and Bortezomib combination (Tumor 3 derived) treatment on tumor 1, 2 and 3.
