# Supplementary material for: Clone-By-Clone Therapy Guidance in Luminal Breast Cancer Via Spatial Proteomics and Patient-Derived Tumoroid
Source: Mol Cell Proteomics. 2026 Aug 12;25(9):101639. doi: 10.1016/j.mcpro.2026.101639 (PMC13579033; doi:10.1016/j.mcpro.2026.101639)
Supplement: Supplementary file 3 [file mmc16.docx]

Clone-by-Clone Therapy Guidance in Luminal Breast Cancer via Spatial Proteomics and Patient-Derived Tumoroid

Laurine Lagache^1^, Antonella Raffo-Romero^1^, Marie Duhamel^1^, Yanis Zirem^1^, Isabelle Fournier^1,3,4*^, Nawale Hajjaji^1,2*^, Michel Salzet^1,3,4*Φ^

^1^ Univ. Lille, Inserm, CHU Lille, U1192, Protéomique Réponse Inflammatoire Spectrométrie de Masse (PRISM), F-59000 Lille, France

^2^ Breast Cancer Unit, Oscar Lambret Center, Lille, France

^3^ Institut Universitaire de France, Ministère de l’Enseignement Supérieur, de la Recherche et de l’Innovation, 1 rue Descartes, 75231 PARIS CEDEX 05

^4^ Equipe Labellisée Ligue Contre le Cancer, Lille, France.

# Supplementary Spreadsheet Legends

**Spreadsheet 1:** **Protein Identification and Quantification Data.** Table of all proteins identified and quantified in this study, including accession numbers, number of unique peptides per protein, and relevant quantification data.

**Spreadsheet 2**: **Proteins Identified in Tumour 1 (Regions A–D).** List of 3,810 proteins significantly identified across regions A, B, C, and D of Tumour 1.

**Spreadsheet 3:** **Differentially Expressed Proteins in Tumour 1 (Regions A, B, D).** List of 1,128 proteins showing significant differential expression in regions A, B, and D of Tumour 1 (ANOVA, p < 0.01).

**Spreadsheet 4:** **Proteins Identified in Tumour 2 (Regions A–E).** List of 4,221 proteins significantly identified across regions A, B, C, D, and E of Tumour 2.

**Spreadsheet 5:** **Differentially Expressed Proteins in Tumour 2 (Regions A–E).** List of 2,031 proteins showing significant differential expression in regions A, B, C, D, and E of Tumour 2 (ANOVA, p < 0.01).

**Spreadsheet 6:** **Proteins Identified in Tumour 3 (Regions A–E).** List of 2,600 proteins significantly identified across regions A, B, C, D, and E of Tumour 3.

**Spreadsheet 7:** **Differentially Expressed Proteins in Tumour 3 (Regions A–E).** List of 976 proteins showing significant differential expression in regions A, B, C, D, and E of Tumour 3 (ANOVA, p < 0.01).

**Spreadsheet 8:** **Combined Proteins Identified Across Tumours 1, 2 and 3.** List of 5,300 proteins significantly identified by integrating proteomic data from all regions of Tumours 1, 2, and 3.

**Spreadsheet 9:** **Combined Differentially Expressed Proteins Across Tumours 1, 2 and 3.** List of 2,748 proteins showing significant differential expression across Tumours 1, 2, and 3 (ANOVA, p < 0.01).

**Spreadsheet 10:** **Proteins Identified in Bulk PDT Tumour Samples 1–3.** List of 5,000 proteins significantly identified by combining bulk protein data from PDT Tumours 1, 2, and 3.

**Spreadsheet 11:** **Overexpressed Proteins in PDT Tumours 1–3.** List of 2,800 proteins significantly overexpressed in PDT Tumours 1, 2, and 3 (ANOVA, p < 0.01).

**Spreadsheet 12:** **PDT Drugs and Their Mechanisms.** Table of drugs used in PDT for this study, including drug category, targeted pathway, mechanism of action, primary protein target, and relevance to breast cancer.

**Supplementary MSI data:** list of *m/z* ions exclusive and shared between MSI clusters in tumor 1, 2 and 3 according upset plot (Figure S2).
